# Do bromine and surface-active substances influence the coastal atmospheric particle growth?

**DOI:** 10.1016/j.heliyon.2024.e31632

**Published:** 2024-05-21

**Authors:** Kristijan Vidović, Samo Hočevar, Irena Grgić, Dino Metarapi, Iva Dominović, Boris Mifka, Asta Gregorič, Balint Alfoldy, Irena Ciglenečki

**Affiliations:** a National Institute of Chemistry, Department of Analytical Chemistry, Hajdrihova 19, 1000, Ljubljana, Slovenia; b Ruđer Bošković Institute, Division for Marine and Environmental Research, Laboratory for Physical Oceanography Chemistry of Aquatic Systems, Bijenička cesta 54, 10000, Zagreb, Croatia; c University of Nova Gorica, Center for Atmospheric Research, Vipavska 11c, 5270 Ajdovščina, Slovenia; d Aerosol d.o.o., Kamniška 39A, 1000, Ljubljana, Slovenia; e Faculty of Physics University of Rijeka, Radmile Matejčić 2, 51000, Rijeka, Croatia

**Keywords:** New particle formation, Particle growth, Apparent formation rate, Instantaneous growth rate, Surface-active substances, Bromine, Organic carbon

## Abstract

New particle formation (NPF) is considered a major source of aerosol particles and cloud condensation nuclei (CCN); however, our understanding of NPF and the subsequent particle growth mechanisms in coastal areas remains limited. This study provides evidence of frequent NPF events followed by particle growth in the middle Adriatic Sea during the summer months at the coastal station of Rogoznica in Croatia. To our knowledge, this is the first study to report such events in this region. Our research aims to improve the understanding of NPF by investigating particle growth through detailed physicochemical characterization and event classification. We used a combination of online measurements and offline particle collection, followed by a thorough chemical analysis. Our results suggest the role of bromine in the particle growth process and provide evidence for its involvement in combination with organic compounds. In addition, we demonstrated the significant influence of surface-active substances (SAS) on particle growth. NPF and particle growth events have been observed in air masses originating from the Adriatic Sea, which can serve as an important source of volatile organic compounds (VOC). Our study shows an intricate interplay between bromine, organic carbon (OC), and SAS in atmospheric particle growth, contributing to a better understanding of coastal NPF processes. In this context, we also introduced a new approach using the semi-empirical 1^st^ derivative method to determine the growth rate for each time point that is not sensitive to the nonlinear behavior of the particle growth over time. We observed that during NPF and particle growth event days, the OC concentration measured in the ultrafine mode particle fraction was higher compared to non-event days. Moreover, in contrast to non-event days, bromine compounds were detected in the ultrafine mode atmospheric particle fraction on nearly all NPF and particle growth event days. Regarding sulfuric acid, the measured sulfate concentration in the ultrafine mode atmospheric particle fraction on both NPF event and non-event days showed no significant differences. This suggests that sulfuric acid may not be the primary factor influencing the appearance of NPF and the particle growth process in the coastal region of Rogoznica.

## Highlights

• For the first time, new particle formation events were associated with the Adriatic Sea.
• Bromine species, in combination with oxidized organic molecules, may contribute to particle growth.
• Surface-active substances have an impact on the growth of atmospheric particles.
• The combination of instantaneous growth rate, apparent formation rate, condensation sink, and chemical analysis reveals intriguing possibilities regarding particle growth.

## 1 Introduction

According to the IPCC (2022) [1], the impact of atmospheric aerosol particles on climate is currently the most significant source of uncertainty in understanding and predicting Earth's climate changes. New particle formation (NPF) has been recognized as the dominant source of atmospheric aerosol particles worldwide, involving the formation of molecular clusters from gases and their subsequent growth to larger sizes [[2], [3], [4], [5], [6], [7], [8]]. The size at which new particles can act as cloud condensation nuclei (CCN) and thus influence climate directly or indirectly [9] depends on both, their nuclei growth rate (GR) and their scavenging by various removal processes.

During the past few decades, atmospheric NPF and particle growth have been observed all over the world [8,10]. NPF events in marine environments are particularly interesting due to their potential impact on the global aerosol particle number populations through gas-to-particle conversion processes and negative feedback on climate [5,[11], [12], [13]]. However, despite extensive research efforts, marine NPF's underlying mechanisms remain incompletely understood [6,7,14].

On the other hand, coastal zones display a distinct type of local NPF, with the most well-studied coastal zone being Mace Head [12,15] on the western coast of Ireland. The extreme NPF and growth events in this region were observed during low tide and in the presence of solar radiation [[16], [17], [18]]. According to Ehn et al. (2010) [19], it has been demonstrated that the extensive coastal NPF followed by the growth can be attributed to the release of precursor gases from exposed seaweed during low tide.

Yet, mechanisms that govern coastal NPF and their growth are still largely unclear. One of the proposed theories in the past was that sulfuric acid (H_2_SO_4_), derived from the oxidation of dimethyl sulfide (DMS) emitted by planktonic algae in seawater, is responsible for coastal NPF and the production of CCN [11,12,20]. In contrast, more recent chamber experiments have shown that the condensable iodine-containing vapors produced by the photolysis of biogenic iodohydrocarbons emitted by algae are the main nucleation agents in coastal aerosol particle formation [16,21]. Furthermore, iodine (I) emissions were found to be associated with coastal NPF in Cap Grim in Tasmania [22], Roscoff in the northwest of France [23], and O'Grove on the northwest coast of Spain [24]. It was observed that NPF was also favored by low tide conditions at these locations. Recent research has shown that condensation of H_2_SO_4_ formed from DMS has less impact on the GR of particles larger than 3 nm, suggesting that other species, such as extremely low and low volatile organic compounds (ELVOCs and LVOCs), may contribute to the growth of newly formed particles [25]. Given that marine and coastal areas serve as a significant source of NPF [6,11,14], it is imperative to comprehend the mechanisms and conditions governing both NPF and subsequent particle growth. Nevertheless, there is still a shortage of relevant observational data from large continental areas in Africa, Southern America, Asia, Australia, and most coastal regions. Moreover, it is worth noting that there is no available observational data on NPF and particle growth around the Adriatic Sea [4].

This study presents evidence of NPF events and their subsequent growth during the summer months in the middle Adriatic Sea at the Rogoznica station in Croatia for the first time. The study aims to contribute to understanding the appearance of NPF and the mechanisms of particle growth by conducting a comprehensive characterization, classification, and identification of the potential primary components involved in this phenomenon. We conducted online measurements, offline particle collection, and thorough chemical analysis. Our results show that the involvement of bromine (Br) and organic compounds can be an important factors in atmospheric particle growth. Moreover, we found that surface-active substances (SAS) affect particle growth and can induce shrinkage of the newly formed particles.

## 2 Materials and methods

### 2.1 Location sampling field site

Sampling was carried out in the area of Rogoznica Lake (43°32″ N, 15°58″ E) as a representative coastal location of the middle Adriatic for monitoring marine and atmospheric processes (Croatia) [26]. Characterized as a remote coastal area, Rogoznica has low annual averages of PM_2.5_ and particle number concentrations of 10 μg m^−3^ and 1000 cm^−3^, respectively. BC concentrations are also low, i.e., below 100 ng m^−3^. Rogoznica is adjacent to Punta Planka, the most prominent point of the Croatian Adriatic coast, where marine and continental air masses mix due to bora and south winds colliding. The open sea currents exhibit maximum strength in this region, with the open sea towards Italy situated in front (Supporting Information Fig. 1). These characteristics exert a significant influence on the NPF process. The topography of this suburban region is characterized by a broad belt of hinterland with fields in the karst landscape and Mediterranean vegetation, along with the low anthropogenic influence.

**Fig. 1:**
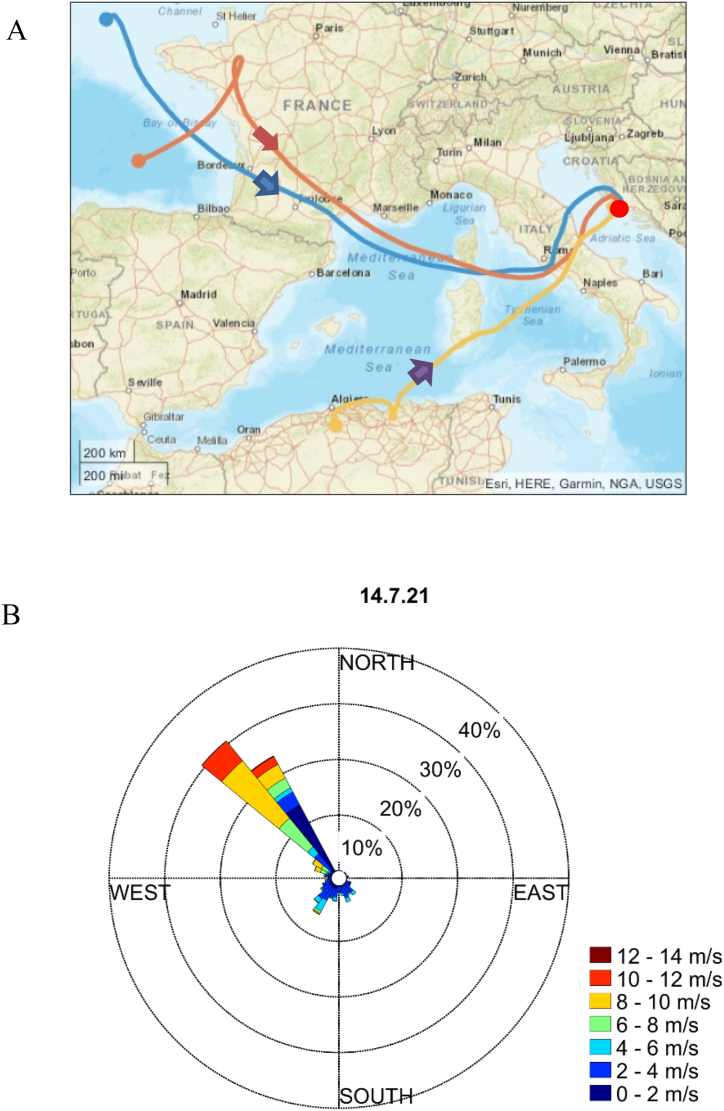
A) Position of Rogoznica with the introduction of NOAA HYSPLIT air-mass backward trajectories on July 14, 2021 (for three different altitudes: blue-100, orange-500, and yellow-1000 m, and 72 h duration), and B) wind rose diagram for the same day observed at Rogoznica. In the wind rose diagram, the colors represent the wind speed, while the percentage shows the frequency of the wind. In all cases, air mass comes from the sea direction when NPF occurs (Supporting Information Fig. 2F and Supporting Information Fig. 25). (For interpretation of the references to color in this figure legend, the reader is referred to the Web version of this article.)

### 2.2 Instruments and measurements

Atmospheric aerosol particles were sampled both, online and offline. Real-time particle size distribution information was obtained through online sampling using an extended differential mobility analyzer (L3081) with the SMPS model 3936 (TSI). The size distribution data were collected continuously every 5 min and sorted into 64 size classes per decade, covering a size range of 14–736 nm.

During the field campaign in the Rogoznica coastal area, real-time equivalent BC mass concentrations were measured using an Aethalometer model AE33 (Aerosol Magee Scientific). The Aethalometer was installed to measure aerosol light-absorption coefficients at seven wavelengths (370–950 nm) with a time resolution of 1 min. The instrument was placed near the SMPS, with a PM_2.5_ size-selective inlet.

The offline sampling was conducted for 24 h using a Berner low-pressure cascade impactor (HAUKE, LPI 25/0.015/2, 25.8 L min^−1^) and a particulate matter (PM_2.5_) sampler (SEQ 47/50-CD-RV; 2.3 m^3^ h^−1^, Sven Leckel Ingenieuburo GmbH, Germany). Various sampling times were tested using a Berner impactor to optimize the collection of sufficient material. The Berner impactor operates with ten stages, each with a different aerodynamic diameter (the lowest being 38 nm). Particles were collected on pre-combusted aluminum foils at each stage of the Berner impactor, while with a PM sampler, they were collected on a pre-combusted glass fiber filter (Whatman, Grade GF/F, d = 47 mm). The Berner impactor ran from 00:00 h to approximately 23:59. At ca. midnight, samples were collected daily, and new aluminum foils were placed in the impactor. The gravimetric analysis was carried out using Sartorius M3P microbalance (reading precision of 1 μg) by weighing the filters before and after the sampling under constant conditions (T = 20 ± 1 °C and relative humidity (RH) of 50 % ± 5 %). After the gravimetric analysis of the collected particles, the samples were stored at −20 °C until further analysis.

The chemical analysis was focused on the first three aerosol size fractions of the Berner impactor, ranging from 38 nm to 160 nm (ultrafine mode atmospheric particles). Before analysis ¾ of the aerosol fractions were extracted in 25 mL of Mili-Q water for 24 h, ultrasonicated, and filtered through a GFF/0.7 μm pr-combusted filter to obtain the water-soluble fraction for further chemical analyses (SAS, WSOC, inorganic ions, organic ions, and trace elements).

The quantification of twelve anions (both organic and inorganic) was performed by a Dionex ICS 3000 ion chromatography system equipped with a conductivity detector. Anion separation was accomplished on an analytical column (Dionex IonPac AS11-HC, 4 × 250 mm) and a guard column (Dionex IonPac AG11-HC, 4 × 50 mm) at a flow rate of 1.0 mL min^−1^. The elution program consisted of an isocratic elution with 1 mM KOH for 0–15 min, followed by a gradient: 15–29 min to 15 mM KOH, 29–42 min to 30 mM KOH, 42–50 min to 60 mM KOH, and finally an isocratic elution of 50–55 min with 60 mM KOH. The injection volume was 50 μL. Under these conditions, 12 anions were quantified: fluoride (F^−^), lactate (C_3_H_5_O_3_^−^), acetate (C_2_H_3_O_2_^−^), formate (CHO_2_^−^), methanesulfonate (CH_3_SO_3_^−^, MS^−^), chloride (Cl^−^), nitrate (NO_3_^−^), maleate (C_4_H_2_O_4_^2−^), sulfate (SO_4_^2−^), oxalate (C_2_O_4_^2−^) and phosphate (PO_4_^3−^).

Elemental analysis was performed using an Agilent 7500ce Series ICP-MS equipped with an octupole collision cell (Agilent, USA). The plasma conditions used were: RF power of 1559 W, sample depth of 10 mm, carrier gas flow of 0.90 L min^−1^, makeup gas flow of 0.15 L min^−1^, nebulizer pump rate of 0.1 rps, and sample pump rate of 0.1 rps. Each sample was measured in triplicate, and the blank value from aluminum foils was subtracted. Five significant elements were detected, i.e., Na, Mg, K, Ga, and Br.

The extracted water samples' surface activity was determined using the electrochemical alternating current (AC) voltammetry method in the out-of-phase mode [27]. This method has been successfully employed to determine SAS in numerous environmental aquatic samples and aerosol extracts. The principle of the method is based on measuring the change of the total capacitive current of the electrical double-layer due to the adsorption of organic material onto a mercury electrode surface. A decrease in the capacitive current at a selected potential in the presence of surface-active organic material relative to the value of pure electrolyte indicates the amount of material adsorbed onto the electrode surface. A calibration plot of the nonionic surfactant Triton-X-100 was used to quantify the surface activity. The surface activity is expressed as the equivalent adsorption effect of a given amount (ng m^−3^) of Triton-X-100.

The concentrations of WSOC were determined by the sensitive high-temperature catalytic oxidation (HTCO) method at 680 °C, employing a Shimadzu total organic carbon analyzer (model TOC-V cph) with a Pt/Si catalyst and calibrated with potassium hydrogen phthalate. This method complies with HRN EN ISO/IEC 17025:2017 (Accreditation Certificate number: 1577) for analyzing waters and sediments.

Normalization of surfactant activity to WSOC content (NSA = SAS/WSOC) provides a framework to assist in monitoring slight changes in organic matter reactivity at spatial and temporal scales in different systems [27]. Furthermore, a comparison of WSOC-normalized surfactant activity (NSA) values for aerosol samples with selected model substances provides a rough estimate of the dominant hydrophobic/hydrophilic organic matter present in the samples.

Further details on the methods and theoretical calculations can be found in the Supporting Information.

## 3 Results and discussion

### 3.1 Observation of NPF in remote coastal area

**Fig. 2:**
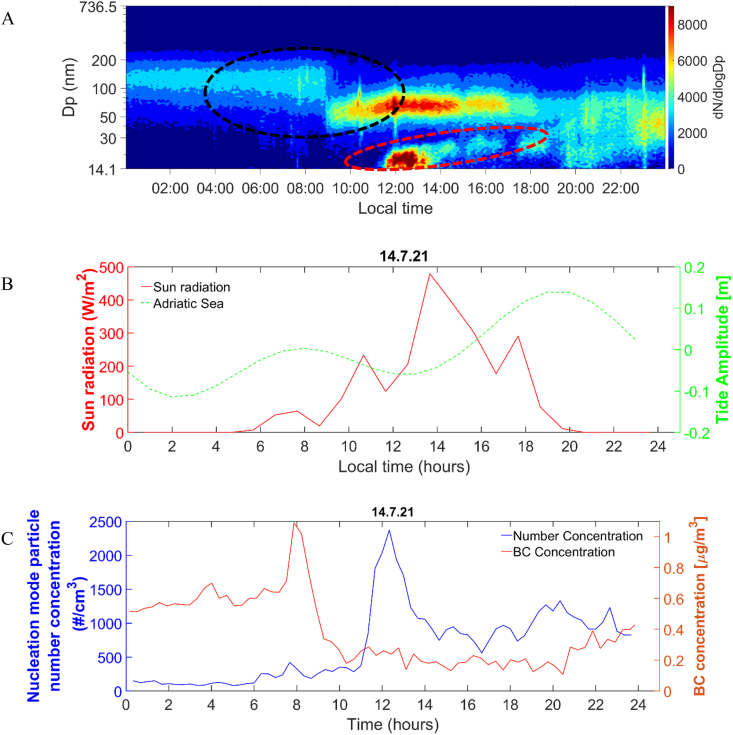
A) Contour plot of particle number size distribution (a black dashed circle indicates the transition between different air masses, while a red dashed circle represents the NPF event characterized by particle growth), B) tidal amplitude and solar radiation, C) nucleation mode particle number concentration and BC concentration (μg m^−3^), observed at Rogoznica on July 14, 2021. (For interpretation of the references to color in this figure legend, the reader is referred to the Web version of this article.)

**Fig. 3:**
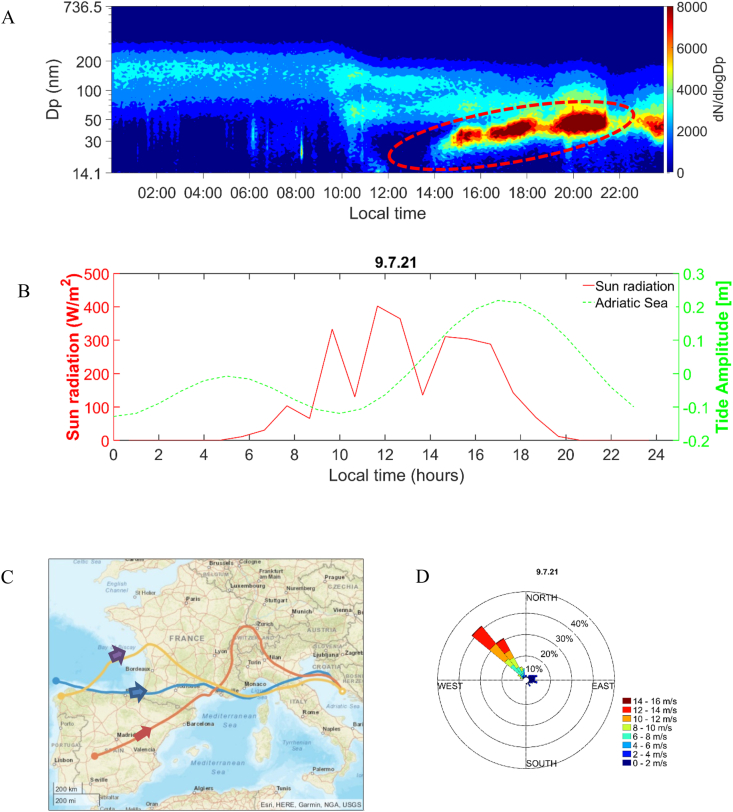
A) Contour plot of particle number size distribution (a red dashed circle represents the NPF event characterized by particle growth), B) tidal amplitude and solar radiation, C) HYSPLIT backward trajectories (for three different altitudes: blue-100, orange-500, and yellow-1000 m, and 72 h duration), and D) the wind roses observed at Rogoznica, on July 9, 2021. The colors in the wind rose diagram represent the wind velocity. The orange trajectory passed through the Po Valley at an altitude of 500 m. (For interpretation of the references to color in this figure legend, the reader is referred to the Web version of this article.)

**Fig. 4:**
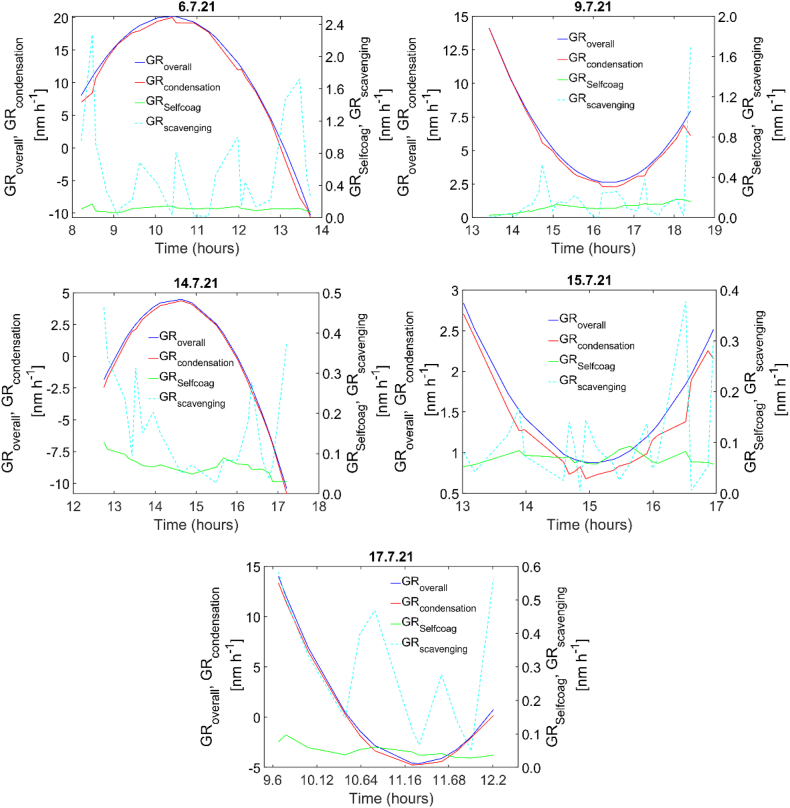
Overall (GR_overall_), condensation (GR_condensation_), self-coagulation (GR_self-coagulation_), and scavenging (GR_scavening_) growth rates observed during each identified NPF event day (characterized by growth) in Rogoznica.

Frequent formation bursts and growth events of atmospheric particles were observed during summer 2021 in the middle Adriatic coastal area near the tourist settlement of Rogoznica, Croatia; the campaign was conducted for a total of 13 days, from July 5 to July 17, 2021 (see Supporting Information Fig. 2). To assess the frequency of NPF events in Rogoznica, we classified all days according to the criteria derived by Dal Maso et al. (2005) [28] and the updated classification by Buenrostro et al. (2009) [29]. We identified Class I events as defined by Dal Maso et al. (2005) or referred to as "NPF event days" according to Buenrostro et al. (2009) [29]. Fig. 2, Fig. 3A (including Supporting Information Figs. 4, 5, 6, 7, and 8) display the contour plots for the days characterized by NPF events (red dashed circles), for which we have obtained information about particle apparent formation and GR (Table 1). It has been noted that NPF event days accounted for 38 % of the observed days (five of the thirteen days). Supporting Information Figs. 9,10, 11, 12, 15,18, 19, and 20 shows events where we could not determine the particle apparent formation and GR, and according to the classification scheme from Dal Maso et al. (2005) [28], they are classified as undefined days. However, by considering the classification of undefined days reported by Buenrostro et al. (2009) [29], we could identify failed events, including quasi (Supporting Information Figs. 9, 10, and 11) and tailed events (Supporting Information Fig. 12) and the pollution-related concentration peaks (Supporting Information Fig. 15). It's worth noting that failed events occurred in 31 %, i.e.,4 days, while the pollution-related event occurred only during one day (8 %) of all investigated days. Altogether, distinct events were observed on 77 %, i.e.,10 days of all days during the measuring campaign. It is important to highlight that three of the thirteen days were classified as non-event days (Supporting Information Figs. 18, 19, and 20) (for more details about the classification, please refer to the Supporting Information). Out of the ten distinct events, the GR of particles in the nucleation mode could be calculated for five events (NPF event days exhibiting particle growth). Note that in some cases, particles in the nucleation mode grew and shrank (Fig. 4). The forthcoming discussion will primarily focus on the occurrence of NPF events and the subsequent particle growth observed during the campaign, as listed in Table 1. Notably, for each NPF event exhibiting particle growth, the particles were transported from the Adriatic Sea area to their location as indicated by the modeled backward trajectories (for three altitudes: blue-100, orange-500, and yellow-1000 m, and 72 h duration) and the wind rose diagrams (Fig. 1A and. 3C, Supporting Information Fig. 25, Supporting Information Fig. 28 and Supporting Information Fig. 2 F). However, the background influence of the eutrophic marine system of the nearby Rogoznica Lake cannot be ignored [26].

**Table 1:** Apparent formation rates (*J*_15_), average GR, and time of the air-mass backward trajectories spent over the sea.

NPF event days	Apparent formation Rate, *J*_15_ [cm^−3^s^−1^]	Average GR [nm h^−1^]	Time spent over the Adriatic Sea area [%]
July 6, 2021	0.039	14.67	4
July 9, 2021	0.010	5.51	5
July 14, 2021	0.042	2.90	5
July 15, 2021	0.024	1.47	2
July 17, 2021	0.009	6.85	12

To differentiate between land-influenced and marine-influenced air-mass backward trajectories, we calculated the time fraction each trajectory spent over the sea surface and the land in 72 h, with a time resolution of 1 h (see Supporting Information: *Air mass back trajectories*). Moreover, using a mask defined by the coordinates of the Adriatic region, we could also calculate how much time the trajectories spend inside the boundaries of the Adriatic Sea. Our analysis revealed that backward trajectories that spent more time above the Adriatic Sea surface (we consider only the lowest height of the trajectories) were associated with higher particle average GR (Table 1). Notably, the modeled backward trajectories indicated that land sources predominantly influenced the air masses during non-events (Supporting Information Fig. 27). This is also supported by the elemental analysis of ultrafine mode particles during the event and non-event days. (Supporting Information, Table S4).

The data presented in Table 1 shows that the particle apparent formation rate (*J*_15_) and average GR fall within the reported range of NPF events observed in remote [11] and boreal forest sites [30,31]. The observed NPF and growth events exhibit variability across a range of conditions, a well-documented finding in the existing literature [4,11,14,32].

Fig. 2 B and Supporting Information Fig. 2 E demonstrate that NPF and growth events occur during low tide periods and maximum solar radiation. Moreover, during the NPF characterized by particle growth, there is a noticeable decrease in black carbon (BC) concentration (Fig. 2C and Supporting Information Fig. 2 B). The decrease in BC concentration lowers the background particles' condensation sink (CS), thereby influencing the condensation of existing vapors. This alteration allows for greater availability of existing vapors to participate in NPF, rather than primarily condensing onto preexisting particles [11]. Additionally, Table S2 (Supporting information) reveals that the CS is lower during the event days characterized by particle growth, indicating favorable conditions for NPF to occur.

Coastal NPF characterized by particle growth is associated with low tides and high solar radiation [12,15,16,18,33]. Furthermore, the modeled backward trajectories terminating at the Rogoznica and the wind roses (Supporting Information Fig. 25, Supporting Information Fig. 28, and Supporting Information Fig. 2 F) indicate that air masses originate from the Adriatic Sea during low tide periods accompanied by increased solar intensity. Considering the occurrence of NPF events characterized by particle growth during low tide and maximum solar radiation and the necessity of adequate vapor presence for NPF and particle growth to occur, it is reasonable to conclude that the Adriatic Sea might act as a potential origin of newly formed particles. This contribution could be attributed to various VOCs acting as precursors for NPF or for initiating particle growth, mainly when other potential sources are ruled out. Four of the five NPF events followed by particle growth showed a consistent pattern of occurrence during low tide and high solar radiation conditions, resulting in more local NPF. However, on July 9, 2021 (Supporting Information Fig. 2 E), the event did not follow this pattern, indicating a different origin of the observed NPF.

The July 9, 2021, event stands out distinctly from other events. This event exhibits greater intensity and persists for more than 8 h. The absence of a nucleation mode in Fig. 3A and the late evening growth pattern indicates that the new particles were formed at a different location and transported to the Rogoznica [29]. All these characteristics indicate that the specific event is associated with a broader regional phenomenon occurring within a larger geographical area. Furthermore, the modeled backward trajectories (the orange trajectory at 500 m in Fig. 3C) reveal that the air masses traveled across the Po Valley, renowned as a significant European pollution hotspot. Notably, the influence of emissions from the Po Valley extends up to approximately 500 km in the southeastern direction within the lower levels of the atmosphere [34]. The coastal-originating NPF followed by particle growth (local; Fig. 2A and Supporting Information Figs. 4A, 7A and 8A) and the regional NPF (Fig. 3A) were visually distinguishable and exhibited fundamental differences in their growth characteristics, as revealed by chemical and physical characterization.

### 3.2 Physical characterization of NPF process exhibiting particle growth at Rogoznica

#### 3.2.1 Apparent formation rate (J_15_)

Table 1 presents the average *J*_*15*_ and GR for each event. The observed *J*_15_ in the studied Adriatic Sea area is significantly lower than in open ocean coastal areas like Mace Head. The *J*_15_ values observed in our study were similar to those found in southern Finland's boreal forests and the Antarctic peninsula [11,31], but significantly lower than *J*_15_ observed in urban areas [35]. Notably, the apparent formation rate under steady-state conditions is consistently lower than the actual nucleation rate, as small clusters tend to coagulate very effectively with pre-existing particles [36]. The apparent formation rate represents the rate at which nuclei appear at larger particle sizes due to their growth by condensation. Therefore, it is always smaller than the real nucleation rate. The apparent formation rate in our study, derived from particles with a diameter of 15 nm, raises a discussion about its comparability with formation rates determined for smaller particle diameters. However, the apparent formation rate of new atmospheric particles (nucleation mode particles) ultimately determines the number concentration of particles that enter the atmosphere through particle formation. The apparent formation rate is of crucial interest because many effects of atmospheric aerosol particles depend on the particle number concentration.

#### 3.2.2 Growth rate (GR)

We calculated the average GR for each NPF event day using the log-normal fitting mode method [3] in combination with the introduced semi-empirical first derivative method. We also used the introduced semi-empirical first derivative method to calculate the instantaneous overall GR and identify evaporation occurrences. The condensation GR accounted for nearly 99 % of the overall GR, indicating its dominance as the primary growth process in the Rogoznica during summer (Fig. 4). Self-coagulation and scavenging rates were negligible due to the low background particle number concentration (Supporting Information, Table S1); this suggests that condensation is the main controlling factor for particle growth in the Rogoznica area (Fig. 4, Supporting Information, Table S2).

The instantaneous condensation GR varies with the particle size, as depicted in Supporting Information Fig. 29; a vital feature of the instantaneous condensation GR can be observed, i.e., the presence of inflection points and sudden changes in the condensation GR as a function of particle diameter. This suggests that particle growth is affected by different particle sizes and species. In almost all cases, the condensation GR increases or decreases until ca. 25–35 nm. Previous studies have suggested that smaller particles' GR differs from larger particles' GR due to the involvement of different species [25].

Furthermore, as shown in Fig. 4, the GR varies over time, with some negative GR. This can be explained in physical terms as the occurrence of the reverse process of condensation, which is known as evaporation within the context of NPF. The shrinkage events have also been observed in the urban typology [37,38]. This phenomenon is primarily attributed to the evaporation of condensed species from the particles. This evaporation is driven by a decrease in precursor vapor concentration, which is influenced by changes in atmospheric conditions such as the mixing layer height [39]. As the mixing layer height fluctuates and interacts with the cleaner layer, it plays a role in diminishing precursor vapor concentration. Consequently, condensed species undergo evaporation, redistributing back to the gas phase from the particles. The shift of the HNO_3_/NH_4_NO_3_ equilibrium is also an essential parameter for evaporation [38]. More about the shrinkage process can be found in the Supporting Information.

#### 3.2.3 Condensation sink (CS)

The CS is a crucial parameter for characterizing particle size distribution's effect upon removing condensable vapor from the atmosphere. Moreover, its magnitude is notably influenced by the shape of particle size distribution [11]. Additionally, the CS impacts the mass balance of atmospheric condensable vapors and can influence the number of condensable vapor molecules that form molecular clusters or participate in the growth process [11,40]. Given that the molecular properties defined by the CS of condensing vapors relevant to the atmosphere remain largely unknown, our study explores the relationship between GR and the molecular properties of potential condensing vapor candidates. These candidates are selected based on chemical analysis of the ultrafine mode particles. By employing this approach, we aim to identify and trace back the specific condensable vapors potentially responsible for the observed growth of nucleation mode particles, thus providing valuable insights into their potential role in this process. Notably, based on the chemical analysis, it was observed that Br^−^ is exclusively present during the growth event days in the ultrafine mode particles. On event days, the concentration of organic carbon (OC) in the ultrafine mode particle fraction was higher compared to non-event days (see Table S5 in the Supporting Information). Consequently, we calculated CS values for a range of marine-coastal bromine-related vapors, such as CHBr_3_ and BrO^−^, as well as for condensable oxidized organic molecules (OOM) for each daily observed particle size distribution (see Supporting Information Fig. 2C). The latter (OOM) represents an averaged surrogate for condensable oxidized organic molecules that may contribute to the measured OC [19,40]. The properties of OOM were selected based on the work of Ehn et al., 2014 [19]. Table S2 in the Supporting Information presents the computed average CS values for selected vapors (H_2_SO_4_, OOM, CHBr_3_, and BrO^−^) corresponding to each daily observed particle size distribution. Remarkably, the average CS values for all vapors consistently exhibit lower values during nearly all NPF event days with particle growth compared to non-event days without particle growth, a phenomenon already observed in the literature [41]. This phenomenon can be attributed to the fact that, with lower CS values, a more significant fraction of condensable vapor becomes available for clustering and the growth of newly formed particles rather than being predominantly used for condensation on the preexisting particles [35,40]. The lower CS value, especially for the condensable vapors (OOM, H_2_SO_4,_ and BrO^−^) during NPF event days exhibiting particle growth, can be partially explained by the concurrent decreases in total particle number concentration, BC concentration and PM_2.5_ concentration (see Supporting Information Fig. 2 and Table S1) during the same days, which typically represent the particle background (preexisting particles) concentration. Although CHBr_3_ is a volatile compound and the CS values should be interpreted cautiously, it is noteworthy that CHBr_3_ is also a water-soluble compound with a solubility in water of 3 g L^−1^. In the case of CHBr_3_, its lower CS compared to H_2_SO_4_ does not necessarily imply its preference to contribute to particle formation or growth over H_2_SO_4_, as it is a volatile compound. Instead, it suggests that CHBr_3_ will be consumed by the particle slower than H_2_SO_4_.

Contrary to that, the higher CS of CHBr_3_ compared to OOM shows that the particles will consume it faster in the loss process. CHBr_3_ is a volatile compound, and the probability of its direct condensation onto the particles is negligible. However, it can be lost through dissolution in the particle liquid layer. Once dissolved, CHBr_3_ can undergo hydrolysis [42], forming low-volatile Br compounds capable of participating in particle growth. The BrO^−^ exhibits the highest CS value compared to OOM, H_2_SO_4_, and CHBr_3_. As this substance is condensable, the highest CS value indicates that it will be lost more rapidly through condensation, making it also capable of participating in particle growth. Since none of these vapors were measured during the campaign, it is challenging to determine their individual contribution to particle growth based on CS comparison alone. Despite the difficulty in identifying the specific dominant Br vapors or OC species, chemical analysis of ultrafine mode atmospheric particles suggests that both Br and OC species likely contribute to particle growth in the Rogoznica area.

### 3.3 Chemical characterization of NPF process exhibiting particle growth at Rogoznica

#### 3.3.1 Ionic analysis of ultrafine mode atmospheric particles

Table 2 highlights variations in the ionic composition of ultrafine mode atmospheric particles between NPF events characterized by particle growth and non-event days without particle growth. It is vital to emphasize that Br^−^ was exclusively detected in the ultrafine atmospheric particle mode (30–100 nm), specifically during NPF event days exhibiting particle growth. It is also noteworthy that Br^−^ has been previously detected in PM_2.5_ samples collected from the Rogoznica area [43]. While CHBr_3_ and its reactive Br species have been demonstrated to impact O_3_ chemistry substantially, their importance in NPF (either particle formation or growth) has remained unexplored. A general understanding of the abundance and sources of reactive Br species outside polar regions is limited due to a lack of observations in various geographical areas. However, a recent study by Men Xia et al. (2022) [44] reported significant levels of Br_2_ at a coastal site in Hong Kong with an average noontime mixing ratio of 5 ppt. They showed that Br_2_ reaction with photolyzed NO_3_^−^ was the most significant primary source of reactive Br species (Br^●^, BrO^−^). Besides, it is also recognized that CHBr_3_ is the primary marine-derived Br organic compound [45], which has also been identified as a significant source of reactive Br species, such as BrO^−^ or Br^●^ [46,47]. Several field and modeling studies have indicated that CHBr_3_ can substantially contribute, i.e., from 30 % to 60 %, to inorganic Br levels (Br^−^, BrO_3_^−^) in the troposphere [47,48].

**Table 2:** Analysis of the major ions (μg m^−3^) in the ultrafine mode atmospheric particle (30–100 nm) collected during summer in Rogoznica. The first three particle fractions from the Berner impactor were extracted and analyzed.

NPF event days	Acetate	Format	Lactate	Maleate	Oxalate	${SO}_{4}^{2-}$	${PO}_{4}^{3-}$	${NO}_{3}^{-}$	${Cl}^{-}$	${Br}^{-}$	$F^{-}$
July 6, 2021	n.d	n.d	n.d	n.d	0.03	0.34	0.002	0.42	n.d	0.55	n.d
July 9, 2021	0.03	0.02	n.d	n.d	0.06	0.61	n.d	0.21	0.001	n.d	0.13
July 14, 2021	0.09	0.07	n.d	0.003	0.04	0.44	n.d	0.16	n.d	0.14	0.15
July 15, 2021	n.d	0.04	0.10	0.45	0.23	n.d	n.d	0.40	0.03	0.12	0.09
**Non-event days**											
July 8, 2021	0.05	0.02	n.d	n.d	0.06	0.6	0.04	0.05	0.008	n.d	0.014
**Tailed event**											
July 11, 2021	0.03	0.02	n.d	n.d	0.05	0.14	0.005	0.09	0.005	n.d	0.139

n.d-not detected.

The concentration of NO_3_^−^ in ultrafine mode atmospheric particles increased during the event days characterized by particle growth. Still, the measured average NO_3_^−^ concentrations were 1.5 times lower than those of SO_4_^2−^ in ultrafine mode atmospheric particles during the event day characterized by particle growth. Interestingly, no significant distinction was observed in the average SO_4_^2−^ concentration in ultrafine mode atmospheric particles between event and non-event days. Moreover, the absence of methanesulfonic acid (MSA) in the ultrafine atmospheric particles, which is a characteristic precursor for non-sea salt sulfate originating from phytoplanktonic emission of dimethylsulfide (DMS), throughout the entire measurement campaign suggests that the observed SO_4_^2−^ is more likely a result of anthropogenic influence rather than being primarily biogenic or sea salt-derived. Additionally, a prior study conducted in the Rogoznica area revealed that sea salt SO_4_^2−^ makes a modest contribution of just 19 % to the overall SO_4_^2−^ content, while biogenic non-sea salt SO_4_^2−^ constitutes a mere 9 % of the total SO_4_^2−^ content [43]. Given the lack of significant differences in SO_4_^2−^ concentrations in ultrafine mode atmospheric particles between NPF events characterized by particle growth and non-event days without particle growth, it is conceivable to speculate that H_2_SO_4_ may not act as a primary component influencing nucleation mode particle growth in Rogoznica. This observation is consistent with previous findings suggesting that H_2_SO_4_ has a lesser impact on the growth of particles larger than 3 nm [49].

The concentration of Cl^−^ remains notably low during both NPF events exhibiting particle growth and non-event days without particle growth. One contributing factor is Cl depletions resulting from acidic displacement reactions, likely accounting for the substantial reduction in Cl^−^ levels. The existing literature has already observed Cl^−^ depletions ranging from 28 % to 93 % in PM_2.5_ samples from Rogoznica [43] due to the acid displacement reaction. Another potential reason could be the size of the particles sampled. Since Cl^−^ is primarily emitted through bubble-bursting mechanisms, it tends to be more concentrated in the accumulation particle mode (specific results are not presented here).

Similar concentrations of PO_4_^3−^ and F^−^ in ultrafine mode atmospheric particles during both NPF event days characterized by particle growth and non-event days without particle growth suggest their insignificant contribution to particle growth compared to other elements. The average concentration of organic acids in ultrafine mode atmospheric particles increased during the NPF event days characterized by particle growth, followed by the same trend of OC (see Table S6). Among the organic acid ions, oxalate was detected on all observed days. It is understood that marine emissions of ethane, isoprene, and other biogenic precursors, in conjunction with subsequent photochemical aqueous phase reactions, contribute to the formation of oxalate in the marine atmosphere [50]. This finding supports the assumption that the Adriatic Sea could serve as a source of VOCs, potentially contributing to the growth process of newly formed particles.

Maleic acid is an unsaturated dicarboxylic acid that can also originate from isoprene emissions and unsaturated fatty acids at the sea surface [51]. It is already recognized that maleic acid can be linked to the NPF and particle growth process in marine-coastal environments [52]. Maleic acid was detected on only two days, whereas lactic acid was observed only on a single NPF and growth event day. Theoretical studies have indicated that lactic acid could significantly affect the clustering process of NPF and support particle growth [53]. Maleic and lactic acid were not detected in the ultrafine mode atmospheric particles during the non-event days when particles did not exhibit growth. Formic and acetic acids are also associated with biogenic emissions from the sea, primarily formed through the ozonolysis of biogenic precursors. From Table 2 it can be seen that their concentrations are slightly increased during the event days when the particles exhibited growth. Kumar et al. (2019) [54] experimentally demonstrated that adding simple organic acids and amines to water can significantly enhance particle formation. These simple acids have also been confirmed to play a significant role in particle growth in Tecamac, Mexico [55].

Given that the smallest observed particle size, sampled by the Berner impactor, is 30 nm, which corresponds to the upper threshold of the nucleation mode particles, a substantial change in chemical composition between events with particle growth and non-event days without particle growth indicates the potential involvement of specific compounds in nucleation mode particle growth during NPF.

#### 3.3.2 Surface-active substances (SAS) analysis

Organic surfactant molecules are frequently found in atmospheric particles, present at the surface as monolayers, patchy islands, or thicker films and coatings [56]. Their role in CCN is known, as they can decrease the surface tension of the liquid layer and influence the Kelvin effect, or they can migrate from the bulk liquid layer to the surface and affect the Raoult phenomenon [57]. However, their role in particle growth during NPF processes is ambiguous, and little is known about them.

The surface activity of ultrafine mode atmospheric particles (30–100 nm) was measured using an established electrochemical method [27] during both events exhibiting particle growth and non-event days without particle growth in Rogoznica. The results in Table 3 demonstrate that the surface activity of ultrafine mode atmospheric particles is higher during the event days characterized by particle growth compared to non-event days without particle growth. Moreover, the highest surface activity values were observed in conjunction with the highest condensation GRs, showing the potential importance of SAS in the growth of atmospheric particles. The observed relationship between surface activity and condensation GRs (*R*^2^ = 0.71) suggests that SAS may play a crucial role in promoting the condensation of organic vapors and the uptake of organic gases onto particle surfaces, influencing particle growth.

**Table 3:** SAS, water-soluble organic carbon (WSOC), and the corresponding WSOC normalized surface activity (NSA = SAS/WSOC) in ultrafine mode atmospheric particles (30–100 nm) during events exhibiting particle growth and non-event days without particle growth in Rogoznica. The first three particle fractions from the Berner impactor were extracted and analyzed.

NPF event days	SAS [ng m^−3^ eq. Triton-X-100]	WSOC [μg m^−3^]	NSA
July 6, 2021	118.77	0.796	0.149
July 9, 2021	18.94	0.275	0.068
July 14, 2021	51.88	0.084	0.612
July 15, 2021	40.79	0.128	0.318
**Non-event days**			
July 8, 2021	28.64	0.347	0.082
**Tailed event**			
July 11, 2021	26.61	0.217	0.122

Carboxylic acids in atmospheric particles are known to exhibit surface activity [56]. They can promote the transport and condensation of hydrophobic VOCs or semi-volatile organic compounds (SVOCs - not completely oxidized gases) [58]. To evaluate the hydrophobicity/hydrophilicity of the sampled ultrafine mode atmospheric particles (30–100 nm), we used the WSOC normalized surface activity (NSA) parameter (Table 3), which is directly related to the hydrophobic/hydrophilic nature of the surface-active molecules [27]; a higher NSA value indicates a higher hydrophobicity.

Our results showed higher hydrophobicity of the ultrafine mode atmospheric particles during the NPF events characterized by particle growth compared to the non-event days without particle growth, suggesting the presence of more partially oxidized molecules [59]. In the presence and increased concentration of carboxylic acids, particularly on July 14 and 15, when maleic and lactic acids were identified, and a higher concentration of overall carboxylic acid was measured (as shown in Table 2), their surface-activity nature can facilitate the condensation of additional SVOCs and/or VOCs onto particle surfaces. This process can lead to an elevated hydrophobicity of the WSOC fraction. In contrast, during periods of absence or reduced concentration of carboxylic acids (specifically on July 6, 9, 8, and 11), when maleic and lactic acids were not detected, and the overall carboxylic acid concentration was 2–8 times lower, (as shown in Table 2), the surface activity of the WSOC fraction within particles changed, leading to a lower hydrophobicity.

## 4 Atmospheric implications

### 4.1 Mechanistic interpretation of ultrafine mode atmospheric particle growth

Based on the chemical analyses of ultrafine atmospheric particles (30–100 nm), their corresponding physical properties (derived by SMPS), and the properties of potential candidate vapors, we can develop a plausible interpretation of the components contributing to the growth of atmospheric particles. The core arguments are derived from the combination of chemical and physical properties observed in atmospheric particles collected on NPF events characterized by particle growth and non-event days when particle growth was absent. It is essential to recognize that while we cannot sample and analyze particles in the lower nucleation mode (1 nm–3 nm), the observed differences between NPF events with particle growth and non-event days without growth in the physical and chemical properties of ultrafine mode atmospheric particles sampled online (from 14.1 nm) and particles analyzed offline (from 30 nm) can still provide valuable insights into the particle growth process. As depicted in Table 2, the presence of Br^−^ ion is observed in ultrafine mode atmospheric particles during nearly all NPF event days characterized by particle growth. In addition, it was observed that days with elevated Br^−^ concentrations are associated with increased particle apparent formation and growth rates, as seen in Table 1 and Fig. 4. Interestingly, when Br^−^ was absent, the NPF event on July 9, 2021, exhibited the lowest particle apparent formation rate (see Table 1). This observation suggests that Br contributes to NPF; it affects the occurrence of NPF events exhibiting growth and influences the variations in particle apparent formation and growth rates. Although no direct measurements of Br vapors were performed, we identified CHBr_3_ and BrO^−^ as potential precursors responsible for the measured Br^−^ ion in the particles and as possible components affecting the particle growth process. As the CS for CHBr_3_ and BrO^−^ increases, the GR also increases (Supporting Information Fig. 24). This observation and the increased Br^−^ concentration in the ultrafine mode atmospheric particles with higher GR suggest that these vapors may play a role in the particle growth process. However, further investigation is required to substantiate these conclusions and to determine the dominant influential vapor.

The correlation between particle growth during the NPF process and Br^−^ concentration can also be attributed to reactive Br species' involvement (Br^●^ and BrO^−^). Recent studies have shown that reactive halogens, including Br, can alter the concentrations of conventional oxidants (such as OH, O_3_, and NO_3_), increasing their reaction rate with gas-phase pollutants, including VOCs [44,60]. This indirectly influences the production of condensable vapors. In addition, reactive Br species can also directly oxidize volatile gasses, forming condensable vapors that contribute to the particle growth process [60]. Thus, when various reactive Br species undergo a series of reactions with VOCs and alkenes, Br^−^ and oxidized low-volatility products may be formed, which can be consumed in the particle growth process [60].

Furthermore, it is recognized that the photolysis of NO_3_^−^ in the presence of Br species significantly contributes to forming reactive Br species. In connection with this, it is interesting to note that in Rogoznica, the concentration of NO_3_^−^ in ultrafine mode particles was higher during NPF event days exhibiting particle growth than non-event days without particle growth. Given that a measurable amount of total Br was detected even on non-event days in the absence of particle growth (see Table S4), while the concentration of NO_3_^−^ was nearly ten times lower, and no Br^−^ ions were detected, it can be inferred that various Br species are present in Rogoznica. Nevertheless, it appears that a critical amount of NO_3_^−^ is necessary to generate reactive Br species, which can further contribute to particle growth and consequently to the presence of Br^−^ ions in ultrafine mode atmospheric particles.

Based on these concepts, where reactive Br species can directly or indirectly oxidize VOCs and produce condensable LVOCs, it can be assumed that such processes are also responsible for the OC increase in ultrafine mode atmospheric particles during the NPF event days characterized by particle growth. Table S5 shows a 50 % increase in OC content of ultrafine mode particles during NPF event days with particle growth when Br^−^ ion is present, as opposed to non-event days without particle growth when Br^−^ was not detected. However, because the concentration of vapors was not measured, we chose the possible precursor that could explain the OC content in the ultrafine mode atmospheric particle fraction, following the work of Tuovinen et al. (2021) [40] and Ehn et al. (2014) [19], who proposed that the OOM could represent an average atmospheric condensable oxidized organic molecule. Additionally, as the concentration of Br^−^ in the particles rises during the growth process, the CS for OOM decreases (as shown in Table 2 and Table S2). If more Br^−^ ion is present in the particles, and assuming that Br^−^ is formed by the reduction of reactive Br species, this indicates that more reactive Br species were likely present in the atmosphere, leading to the oxidation of more VOCs, thus providing more OOM available for particle growth. The GR does not correlate with the amount of OC. This lack of correlation may be due to the influence of other species that also affect the GR.

Reactive Br species or Br^−^ alone are unlikely solely responsible for particle growth. Instead, reactive Br species could play a role in the oxidation of VOCs, producing LVOCs. In line with the observations and the existing literature [61], we can presume that LVOC (or OOM) can have a role in particle growth in Rogoznica. It should be noted that particle growth may be influenced by various organic vapors containing Br, as indicated by the CS analysis and the correlation of GR with the CS for CHBr_3_ and BrO^−^. On the other hand, SO_4_^2−^ does not directly correlate with particle growth rates or CS, and there was no substantial difference in concentration between NPF events exhibiting particle growth and non-event days without particle growth. Therefore, it is reasonable to assume that SO_4_^2−^ is not the primary driving factor for the growth of ultrafine mode atmospheric particles but can still play a role in this process.

### 4.2 SAS involvement in the ultrafine mode atmospheric particle growth

Table 1, Table 3, and Fig. 4 illustrate a positive correlation, showing that as the surface activity of the ultrafine mode atmospheric particles increases, the growth rate also increases. This relationship suggests the involvement of SAS in the growth of newly formed particles. When adsorbed onto the particle surface, SAS can promote gas transport to the particle's surface and potentially contribute to the growth process. Depending on the nature of the SAS, they can also enhance the condensation of incompletely oxidized organics (such as SVOCs or VOCs), resulting in the apparent growth of particles. It has been reported that surfactants possess the inherent ability to control the crystal growth of nanomaterials [62]; their impact on particle growth is determined by the specific functional groups present and the carbon atom chain length within these SAS [63,64]. Based on the NSA parameter, we estimate the nature of the SAS in the ultrafine mode atmospheric particles (see Supporting Information Fig. 22). Table 3 demonstrates that particle growth event days with higher particle hydrophobicity exhibit a lower GR than those with lower particle hydrophobicity (see Table 1, Fig. 4, and Supporting Information: *Interpretation of July 6 and 9 events*).

Additionally, the data in Table 1, Table 3, and Fig. 4 collectively illustrate the correlation between particle growth, the relative concentration of SAS, and their chemical nature. On the growth event days with lower particle hydrophobicity, particle growth reaches up to 100 nm (see Fig. 3A, Supporting Information Fig. 2 A, 4 A and 5 A). In contrast, on days with higher particle hydrophobicity, particle growth is limited to around 30 nm (see Fig. 2A, Supporting Information Fig. 2 A, 6 A, 7 A, 8 A, and Supporting Information: *Interpretation of July 6 and 9 event*). It can be inferred that particles grow up to 30 nm during days of higher particle hydrophobicity due to the presence of specific SAS. Additionally, Table 2 shows that the carboxylic acid content is higher on days with higher particle hydrophobicity than on days with lower particle hydrophobicity. Smaller carboxylic acids are more effective in transporting SVOC or VOC from the gas phase to the particle surface, resulting in increased hydrophobicity. On the other hand, longer carbon chains of SAS produce more compact layers due to stronger hydrophobic interactions among the chains, thus leaving little room for SVOCs or VOCs penetration [56,63]. It should also be mentioned that as the hydrophobic chain length increases, the interaction with the gas becomes less favorable due to the increased entropy of the system [65]. Therefore, in the presence of specific SAS (short-chain carboxylic acids), partially oxidized organics, e.g., SVOCs or VOCs, can condense onto the particle surface *via* hydrophobic-hydrophobic interactions, leading to apparent particle growth [66]. Such interactions have a specific capacity that mainly depends on the size of the hydrophobic chain and the environmental conditions [65], which can be why particles grow up to 30 nm during growth events with higher particle hydrophobicity. Moreover, partially oxidized molecules on the particle surface may increase the barrier for further condensation and inhibit particle growth [67]. In addition, the contour plots in Fig. 2A and Supporting Information Fig. 6 A, 7 A, and 8 A show that evaporation occurred in almost all cases where the particles grew up to 30 nm in size. It can be noticed that the evaporation rate increases (see Fig. 4 and Table 3) as the hydrophobicity increases. Therefore, the evaporation effect can be attributed to weak hydrophobic-hydrophobic interactions, which can degrade upon an increased ambient temperature. In support of this, Supporting Information Fig. 30 illustrates the impact of temperature on condensation growth rates. As depicted, temperature influenced the growth rate during the July 9, 2021 event (characterized by lower hydrophobicity), favoring condensation at lower temperatures, while no evaporation occurred. In contrast, during the July 14, 2021 growth event (characterized by higher hydrophobicity), evaporation occurred as the temperature increased. It can be speculated that during the increased temperature, the weak hydrophobic-hydrophobic bonds between SVOC or VOC and SAS degraded, resulting in the evaporation of SVOCs and VOCs.

The evaporation can also result from acid/base equilibrium shifts. Previous studies [38] have shown that shifting the HNO_3_/NH_4_NO_3_ equilibrium towards HNO_3_ can lead to particle shrinkage due to evaporation. During the July 14, 2021, growth event, where evaporation was observed (see Fig. 4), the concentration of NO_3_^−^ was lower (see Table 2). The decrease in NO_3_^−^ concentration can be attributed to the equilibrium shift, which, along with the degradation of hydrophobic-hydrophobic bonds, can contribute to the evaporation effect. There are also other possible mechanisms causing evaporation, as described in the Supporting Information.

## 5 Conclusion

Frequent growth events were observed in the coastal region of Rogoznica, Croatia, using the evolved size spectra of atmospheric particles in the range of 14.1–736.5 nm measured with the SMPS. A correlation was observed between all growth events, maximum solar radiation, and the tidal effect, which is characteristic of NPF in coastal areas. During each growth event, modeled backward trajectories and wind directions indicated the arrival of air masses from the Adriatic Sea. This suggests that the Adriatic Sea is a source of precursors facilitating particle growth. In addition, as the trajectories spent more time over the Adriatic Sea, the growth rate was higher. The semi-empirical first derivative method introduced to calculate instantaneous growth rates revealed that condensation primarily drove particle growth. Coagulation was considered a minor factor due to the low particle number concentration. The chemical analysis of offline sampled particles during particle growth events and non-events reveals a clear pattern. In particular, Br was present in the ultrafine mode particles during almost all growth events, while it was absent during the non-event days. The Br concentration was accompanied by an increase in the OC concentration compared to non-event days. Furthermore, the increase in Br concentration in ultrafine mode particles was followed by an increase in the average growth rate, indicating a relationship between the two. The formation of reactive bromine species from CHBr_3_ has been suggested as a possible source of Br and OC in ultrafine mode particles during the growth events. Reactive bromine species can oxidize VOCs to form Br and LVOCs, contributing to particle growth. A portion of the LVOC fraction, which exhibits surface activity (SAS), has been shown to contribute to the growth of the particles. The particles exhibited different growth mechanisms depending on the nature of the surface active LVOC (hydrophilic or hydrophobic). More hydrophobic SAS led to particle growth up to 30 nm, whereas more hydrophilic SAS led to particle growth up to 100 nm. In the case of hydrophilic SAS, they form more compressible films with lower barriers to gas-particle transport, resulting in higher particle condensation growth rates. Due to the attractive forces between the hydrophobic tails, hydrophobic SAS forms a dense, incompressible film. This film can act as a barrier to gas-to-particle mass transfer, reducing particle condensation growth rate.

## Data availability

Measurement data for the analyses and figures in this study are archived in the institute's repository and are available upon request.

## Code availability

The codes for data processing are available upon request.

## CRediT authorship contribution statement

**Kristijan Vidović:** Writing – review & editing, Writing – original draft, Visualization, Methodology, Investigation, Formal analysis, Data curation, Conceptualization. **Samo Hočevar:** Writing – review & editing, Funding acquisition. **Irena Grgić:** Writing – review & editing. **Dino Metarapi:** Formal analysis. **Iva Dominović:** Formal analysis. **Boris Mifka:** Writing – review & editing, Formal analysis. **Asta Gregorič:** Writing – review & editing. **Balint Alfoldy:** Writing – review & editing. **Irena Ciglenečki:** Writing – review & editing, Funding acquisition.

## Declaration of competing interest

The authors declare that they have no known competing financial interests or personal relationships that could have appeared to influence the work reported in this paper.
