# Supplementary material for: Do bromine and surface-active substances influence the coastal atmospheric particle growth?
Source: Heliyon. 2024 May 21;10(11):e31632. doi: 10.1016/j.heliyon.2024.e31632 (PMC11140702; doi:10.1016/j.heliyon.2024.e31632)
Supplement: Multimedia component 1 [file mmc1.docx]

**Supporting information for**

**Do Bromine and Surface-Active Substances Influence the Coastal Atmospheric Particle Growth?**

Kristijan Vidović^*^, Samo Hočevar^**^, Irena Grgić, Dino Metarapi, Iva Dominović, Boris Mifka, Asta Gregorič, Balint Alfoldy, Irena Ciglenečki

**^*^Correspondence to**: [kristijan.vidovic@ki.si](mailto:kristijan.vidovic@ki.si)

**Co-correspondence to: [samo.hocevar@ki.si](mailto:samo.hocevar@ki.si)


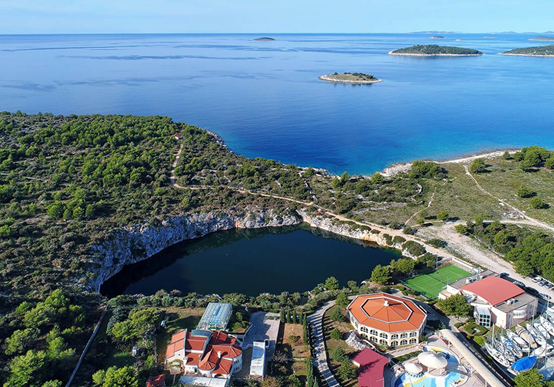


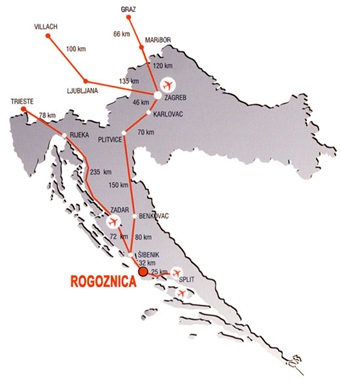


Supporting Information Figure 1. Rogoznica Lake in the middle Adriatic orientated towards Italy [[1](#_ENREF_1)].
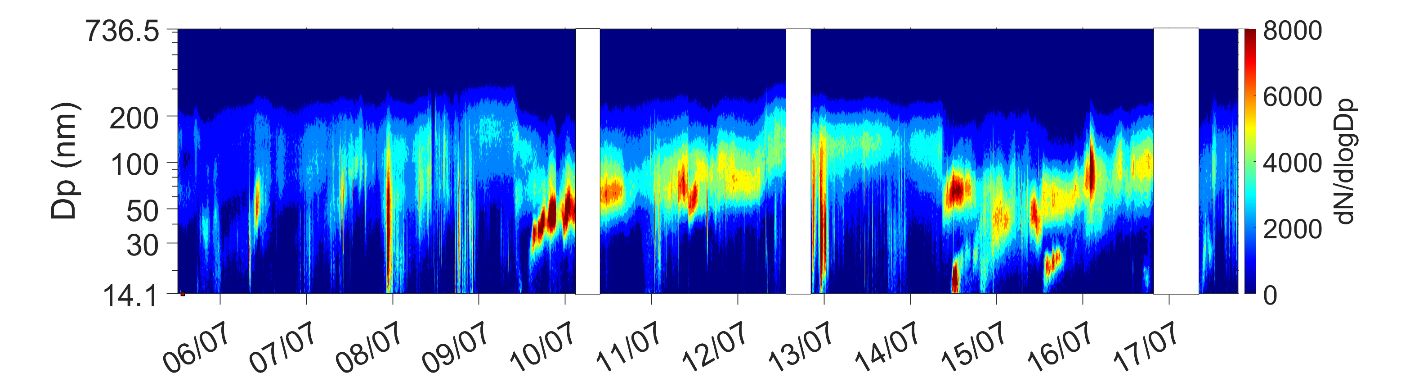

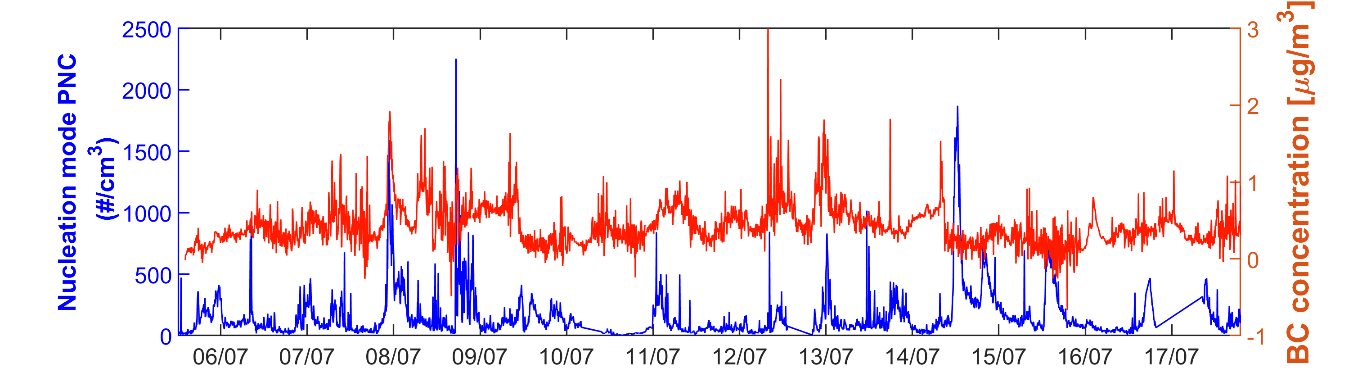


A

B


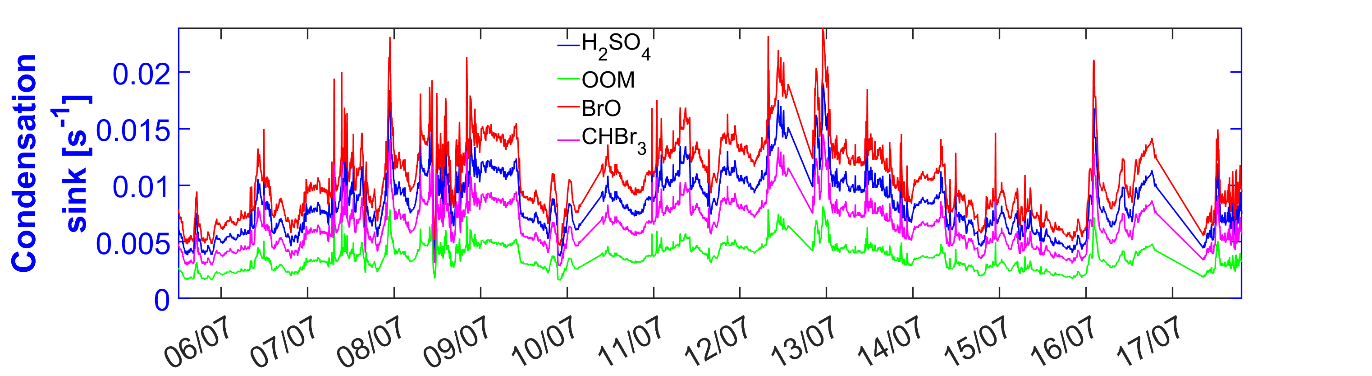


C


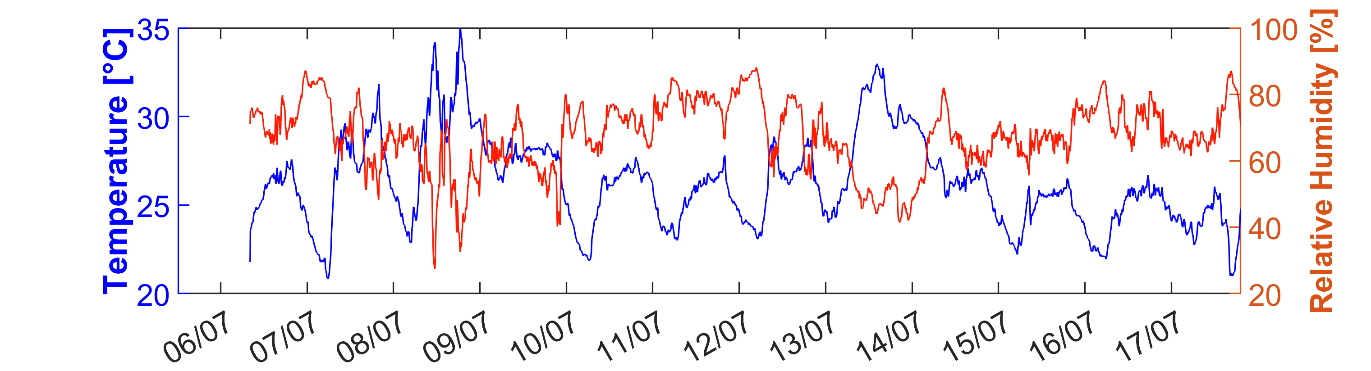


D


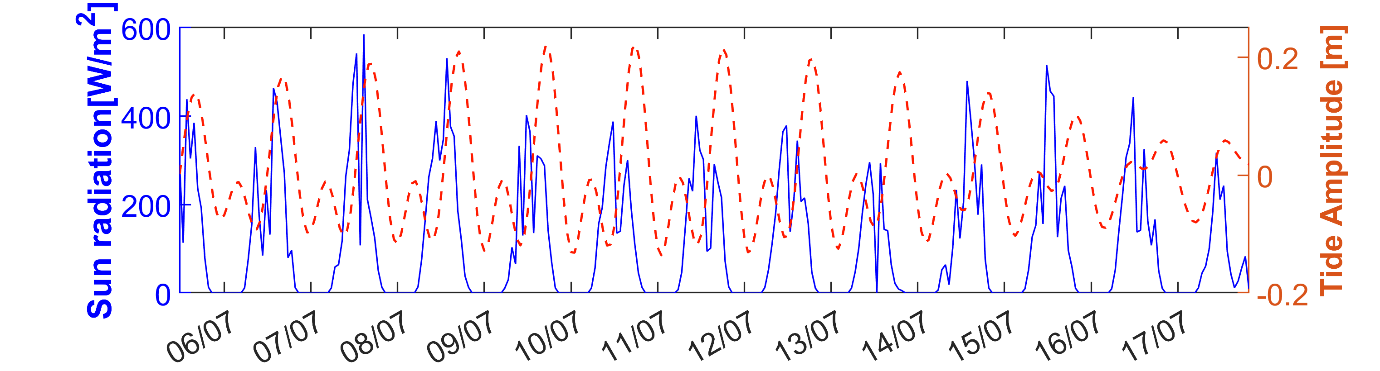


E


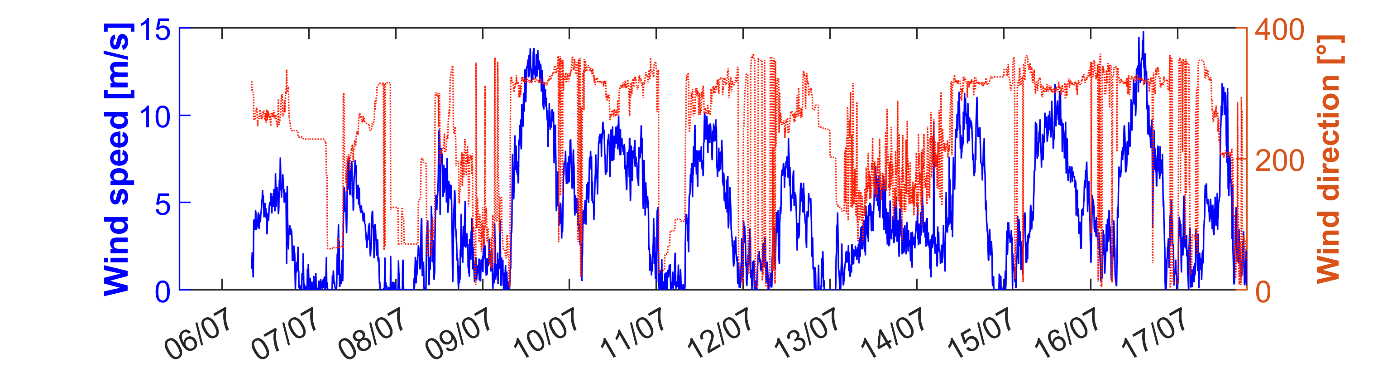


F

Supporting Information Figure 2. A) Contour plot of particle number size distribution for the 13-day run of SMPS, B) nucleation mode particle number concentration and BC concentration for the 13-day run of SMPS and Aethalometer, C) Condensation sink for different vapor candidates ( sulfuric acid- H_2_SO_4_, bromoform – CHBr_3_, hypobromite – BrO^-^ and model oxidized organic molecules-OOM), D) ambient temperature and relative humidity (RH) recorded throughout the measurement campaign, E) sun radiation and tidal amplitude recorded and calculated throughout the measurement campaign and F) the measured wind speed and wind direction. The shaded rectangle represents the time frame of NPF event days occurrence.

Supporting Information Figure 2 illustrates that nearly all NPF event days occurred during a decrease in BC concentration, a necessary precondition reducing the CS of the background particles and enabling vapors to participate in particle formation rather than condensing onto preexisting particles. Supporting Information Figure 2 confirms that the CS for all vapor candidates decreases as the BC concentration decreases on almost all NPF event days. Moreover, it is essential to note that almost all NPF events happened during the low tidal amplitude, a known characteristic of coastal NPF events [[2](#_ENREF_2)]. Each NPF event day occurred when the wind direction shifted to the west and northwest. The link between wind direction and the tides' influence on NPF events suggests that almost all NPF event days observed in Rogoznica are connected to the Adriatic Sea.

Exceptions to this pattern are the NPF events on July 6 and July 9. Despite the event on July 6 occurring during low tide with the wind direction from the west-northwest, the CS exhibited an increasing trend during NPF. Also, the BC concentration showed an increasing trend during the NPF event. The behavior of CS and the measured BC concentration on the NPF event day, July 6, strongly suggest a significant anthropogenic influence. Additionally, the high coagulation sinks and coagulation growth rate (Table S2 and Figure 4), particularly notable on July 6, indicate an increased interaction of smaller particles with the background particles. On the other hand, the wind direction from the west-northwest and the link to the tidal amplitude indicate that biogenic emissions could also influence the event on July 6. Analysis of the contour diagram in Supporting Information Figure 2 suggests that the smallest particles are formed elsewhere or emitted directly (indicated by the prevailing wind direction, possibly originating from the Adriatic Sea area). However, their growth appears to be primarily initiated by anthropogenic emissions, complemented by biogenic emissions, as evidenced by the growth occurring during periods of low tide amplitude.

The July 9 event occurred during decreasing BC concentration and decreasing CS. The sudden change in wind direction from east to northwest could explain the absence of lower end of the nucleation mode particles. A growing particle population appeared after the wind shifted from east to northwest. Although the wind direction indicated that the growing particle population probably originated from the Adriatic Sea area, no relation to the tidal amplitude was observed. It should be noted that the main difference between July 6 and July 9 is the duration of the NPF event. The July 6 NPF event spans over 4 to 5 hours, while the NPF event on July 9 lasts more than 10 hours. This suggests that the July 6 event is more localized, influenced by local emissions (biogenic or anthropogenic), while the NPF event on July 9 is part of a regional event that occurs over a larger geographical area [[3](#_ENREF_3)]. These two events also exhibit different chemical and physical characteristics compared to the other NPF event days, as shown later.

**Observation Data- Classification of the NPF events**

All events were classified using the schemes derived by Dal Maso et al. (2005) [[4](#_ENREF_4)] and the upgraded and adapted scheme by Buenrostro et al. (2009) [[5](#_ENREF_5)], as illustrated in Supporting Information Figure 3.


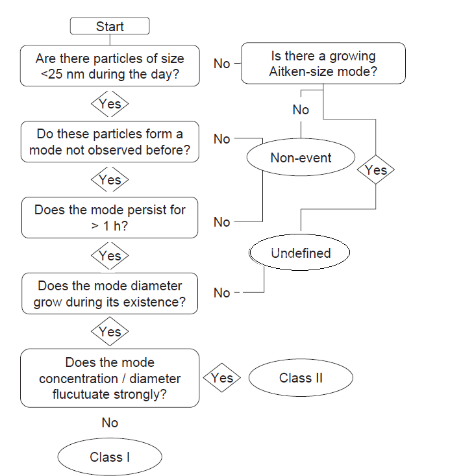

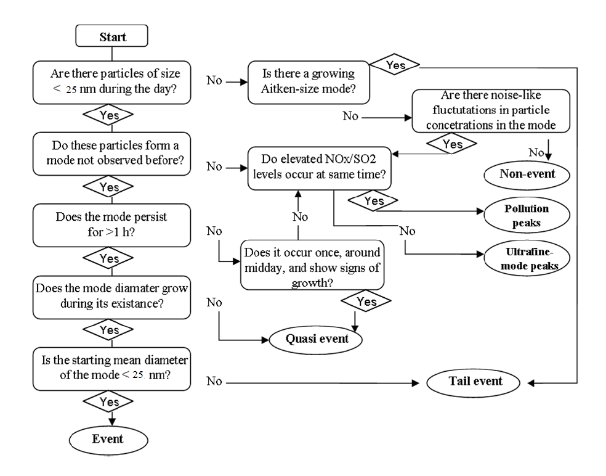


A

B

Supporting Information Figure 3. A) the Classification scheme derived by Dal Maso et la. (2005) [[4](#_ENREF_4)] and B) the upgraded and adapted classification of the undefined days defined by Buenrostro et al. (2009) [[5](#_ENREF_5)].

**NPF event days**

Dal Maso et al. (2005) [[4](#_ENREF_4)], Buenrostro et al. (2009) [[5](#_ENREF_5)], and other authors [[6](#_ENREF_6), [7](#_ENREF_8)] agree that NPF events are characterized by a new mode of particles in the nucleation size range showing persistent growth over hours.

*July 6, 2021*

On July 6, 2021, we did not observe a burst of particles at the smallest measurable diameter. Instead, particles in the nucleation mode (ca. 20 nm) appeared and exhibited signs of growth. In Supporting Information, Figure 4 A (white circle), a slight increase in the number concentration of nucleation mode particles (ca. 20 nm) at around 5:30 (from 65 to 174 particles/cm³, see Supporting Information Figure 4 D-black circle) can be noticed. Their diameter gradually increases (see Supporting Information Figure 3C-black circle) until 8 a.m. These particles are likely formed elsewhere and transported to the measurement station or are primarily emitted by the sea (bubble bursting). At around 8 a.m., two prominent red spikes corresponding to BC emissions appeared (see Supporting Information Figure 3B- blue circle). Simultaneously (around 8 a.m.), the growth rate of the particle mode up to 20 nm increases, leading to the growth of particles to larger diameters (see Supporting Information Figure 3C-red circle). The primary emissions interact with the nucleation mode particles, as evidenced by the coagulation sink and growth rate (see Table S2), which are notably higher than those in other NPF events.

*
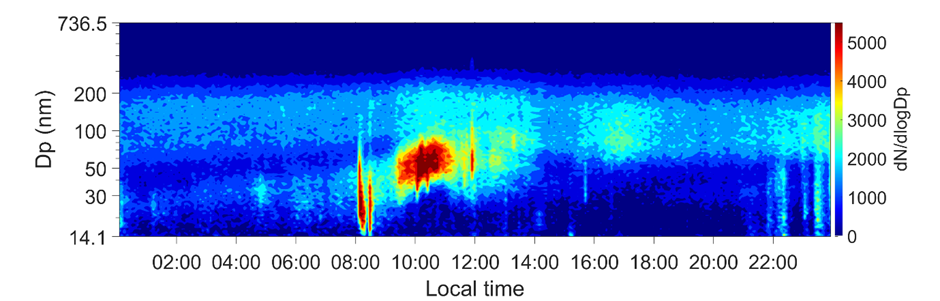
*

A

*
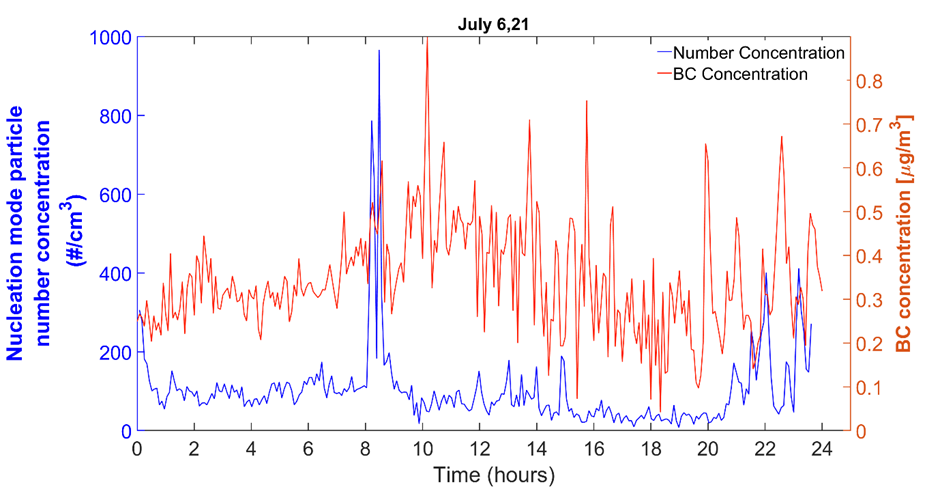
*

B

*
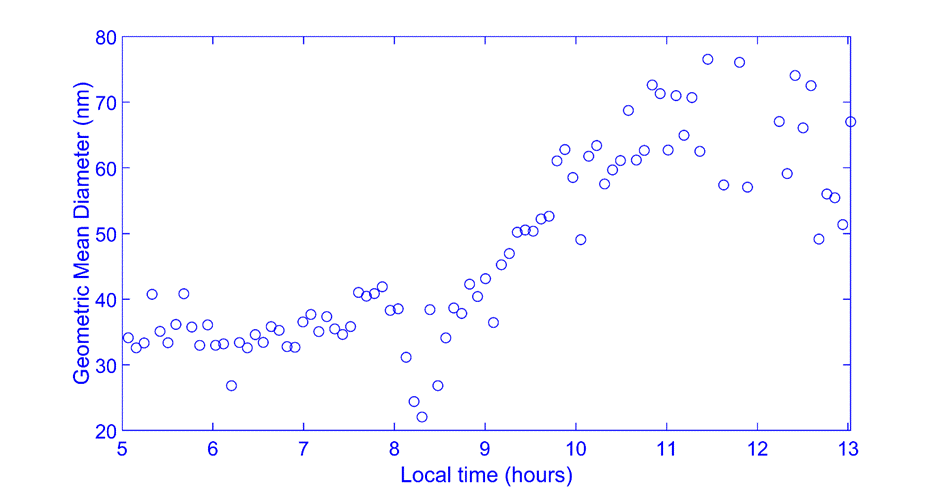
*

C

*
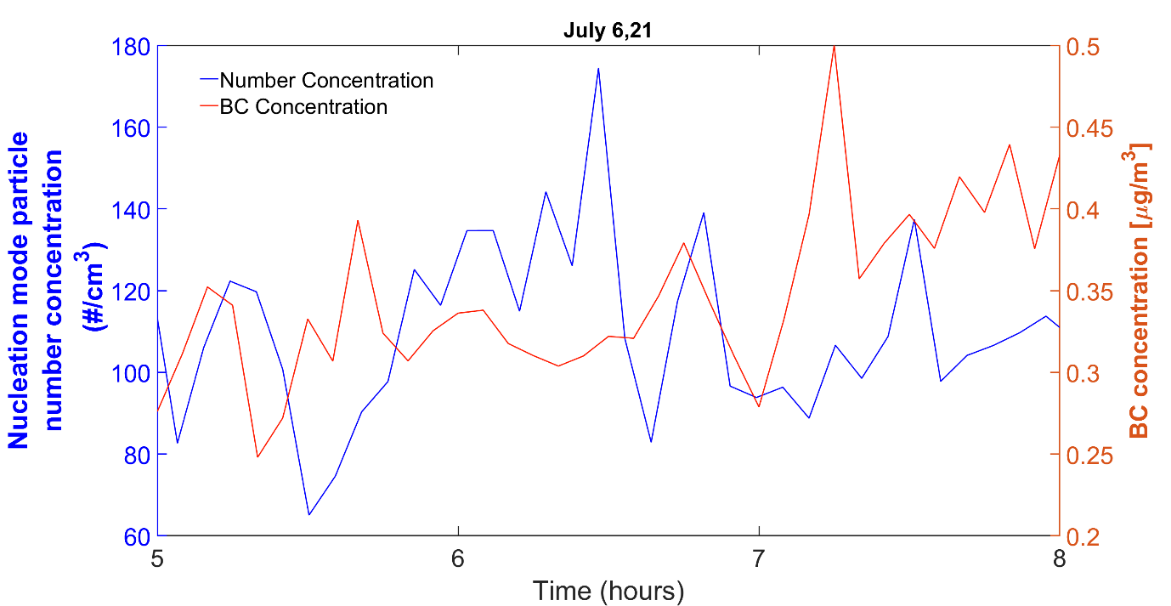
*

D

Supporting Information Figure 4. A) Contour plot of particle number size distribution, B) nucleation mode particle number concentration and BC concentration, C) Geometrical Mean diameter vs. time for the NPF event day on July 6, 2021, and D) nucleation mode particle number concentration and BC carbon from 5 to 8 h on July 6, 2021. The black circle shows the slight increase in particle number concentration. The white circle in A shows a slight increase in nucleation mode particle number concentration. The blue circle in B show the spikes corresponding to BC emission. The black circle in C shows a slight increase in GMD between 5 h and eight h. The red circle in C shows the GMD increase from 8 h to 12 h.

Here, it is debatable whether the appearance of nucleation mode particles at 8 a.m. results from their formation (at the location) or if the mode observed from 5:30 a.m. further increases in diameter by 8 a.m. due to anthropogenic influence. However, in either case, particles in the nucleation mode are growing (primarily by condensation; see the manuscript), resulting in new particles (different in chemical and physical characteristics from the original ones). It is more likely that the presence of particles in the 15 – 20 nm size range (observed from 5:30 h) indicates primary emission or recent formation at various locations and their subsequent transportation to the measurement site. Due to the limitations of our instruments, we cannot directly measure critical cluster formation, preventing us from discussing the nucleation of freshly formed particles. Instead, we can estimate the apparent formation rate (as defined by Kulmala et al., 2004 [[8](#_ENREF_9)]; see also C-H. Jeong et al., al 20102 [[9](#_ENREF_10)]) of particles appearing at larger diameters.

Whether the absence of a particle burst at smaller diameters is due to air mass inhomogeneity, coagulation with primary emitted particles, or particle transport from other locations, it's important to note that particles at 20 nm still fall within the nucleation mode and exhibit growth (see Supporting Information Figure 4C). As a result, this event satisfies the criteria for NPF as defined by Dal Maso et al. 2005 [[4](#_ENREF_4)], where it is explicitly emphasized that NPF events always entail particle growth.

*July 9, 2021*

The July 9, 2021, event shows differences from other events in all characteristics.


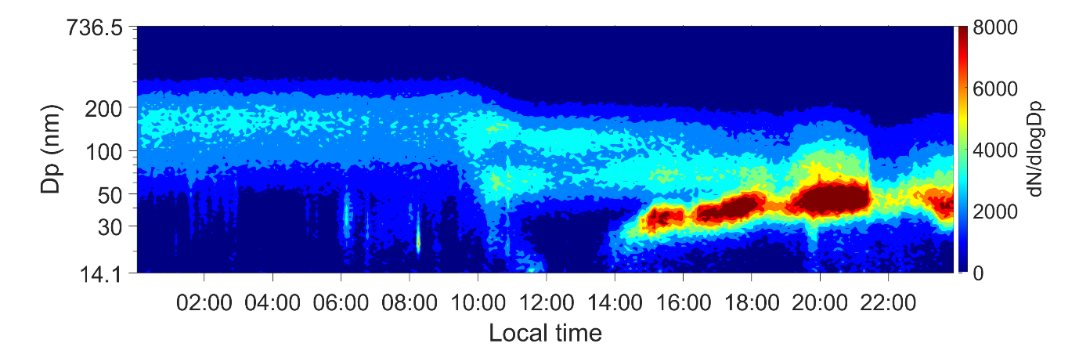


A


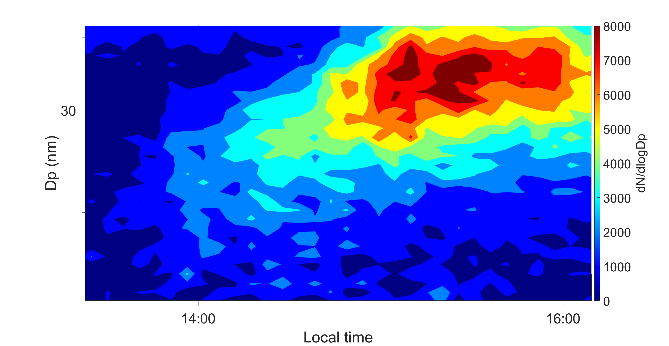


B


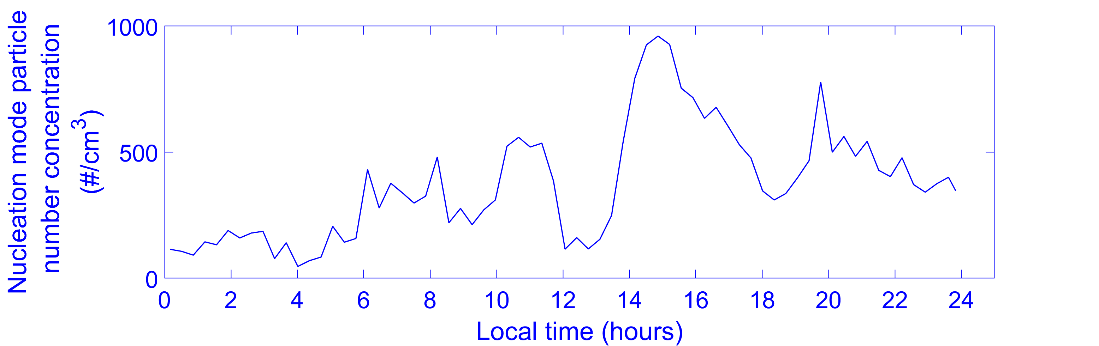


C


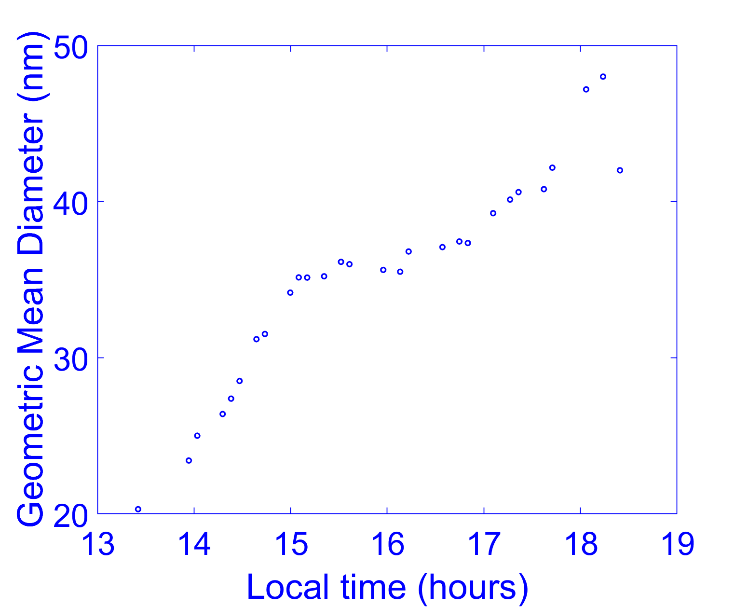


D

Supporting Information Figure 5. A) Contour plot of particle number size distribution, B) nucleation mode particle number concentration, C) Zoomed contour plot from 14 to 16 h, and D) the geometrical mean diameter of the nucleation mode particles for July 9, 2021. The red circle represents the NPF event, showing the appearance and growth of nucleation mode particles. The black circle shows the increase in nucleation mode particle number concentration.

First of all, it can be noticed that this is a robust event whose duration is longer compared to other events. The absence of higher concentrations of the smallest particles (burst of particles) in the size distribution may result from air mass inhomogeneity or different initiation locations of the NPF process. In the case of July 9, 2021, it can be seen from the contour plots that high particle concentration appears at a diameter of around 20 nm and that the mode exhibits strong growth (red circle). However, Supporting Information Figures 5 C and D show that around 14 h, nucleation mode particles appear (black circle) and grow in concentration and size. These characteristics align with the classification scheme, confirming this as an NPF event on July 9, 2021. It should be pointed out that the July 9, 2021, event has very similar characteristics to the tail event. However, the smallest detectable particle sizes are observed in the discrete particle size data set but in low concentrations (see Supporting Information Figure 5B), probably because of the air mass inhomogeneity. Supporting Information Figure 5B (see also Supporting Information Figure 2) shows the zoomed contour plot from July 9, 2021, from 14 to 16 h. It can be noticed that there are particles smaller than 20 nm present which exhibits growth. Based on this and the event occurring around noon, we believe it is more plausible to classify it as an NPF event rather than a tail event.

*July 14, 2021*


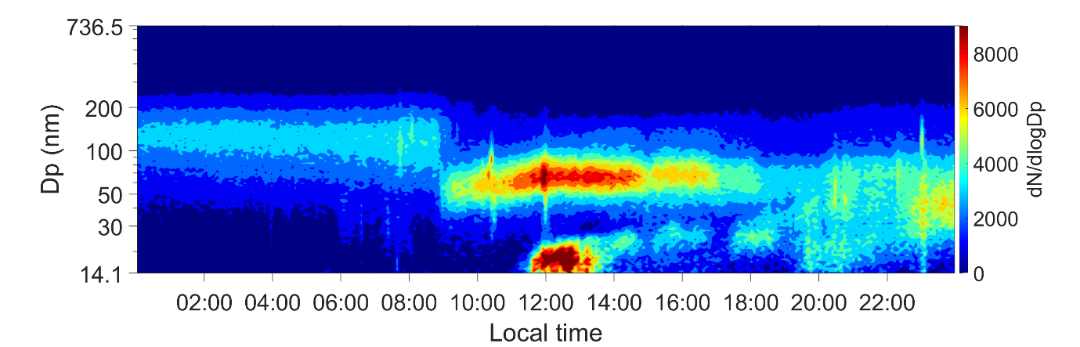


A

**
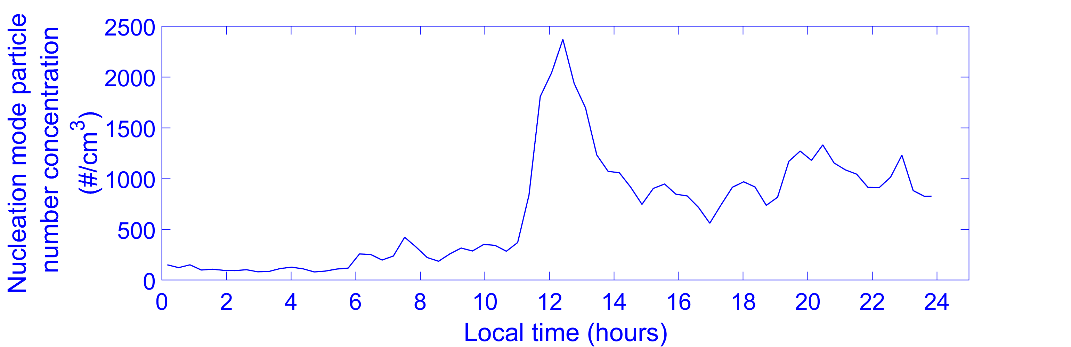
**

B

**
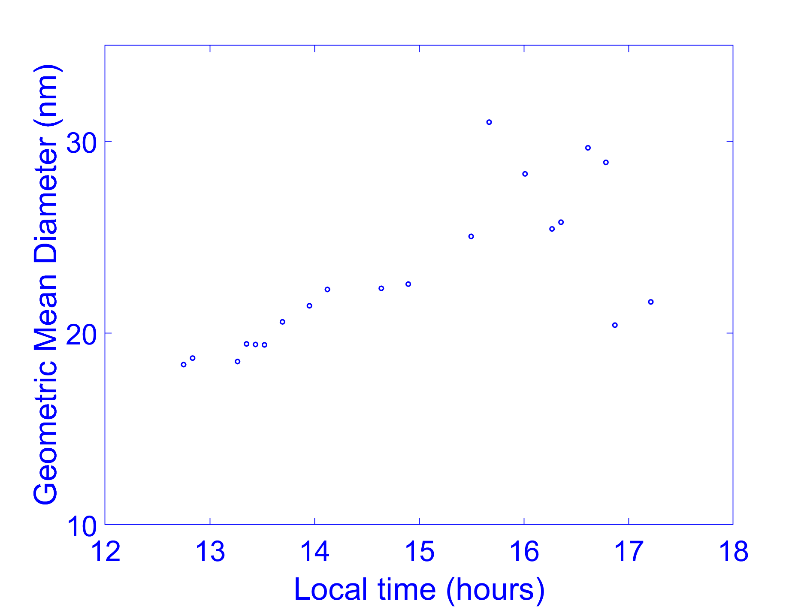
**

C

Supporting Information Figure 6. A) Contour plot of particle number size distribution, B) nucleation mode particle number concentration, and C) the geometrical mean diameter of the nucleation mode particles for July 14, 2021. The black circle represents the appearance and increase of nucleation mode particle number concentration, while the red circle shows the particle burst and the subsequent growth.

Supporting Information Figure 6 clearly shows the appearance of particle burst at the lowest measured particle diameter (black circle) and the subsequent growth of the formed particles (red circle). Therefore, the July 14, 2021, event undoubtedly aligns with the classification scheme in Supporting Information Figure 3 as NPF events.

*July 15, 2021*

*
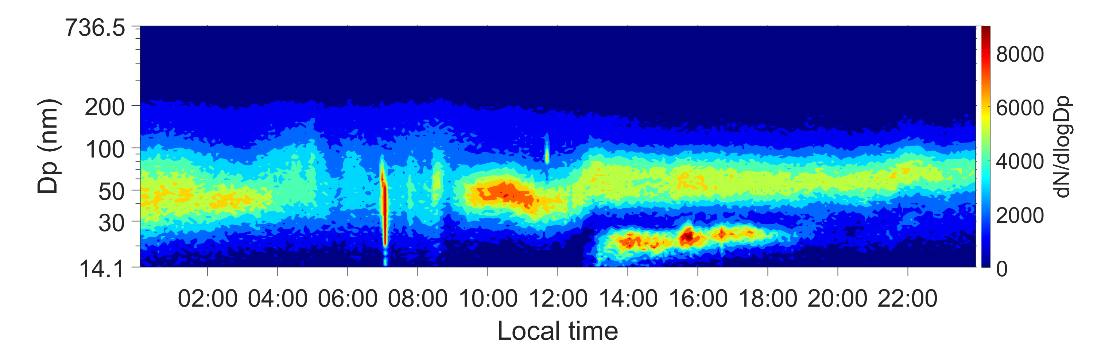
*

A


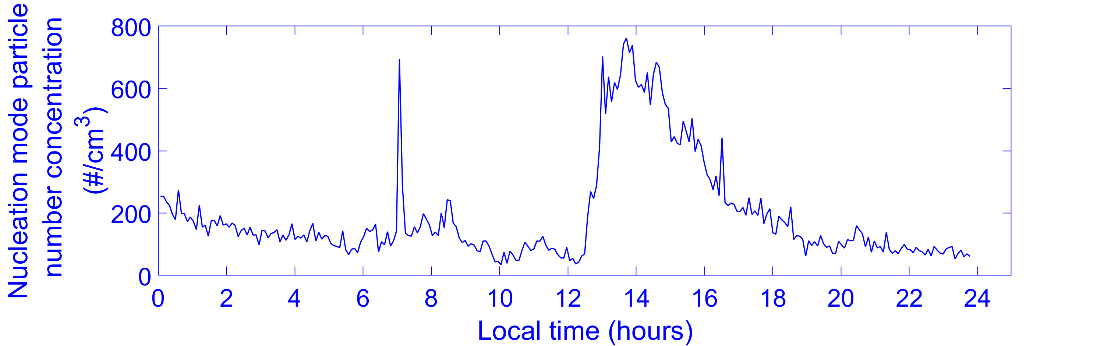


B


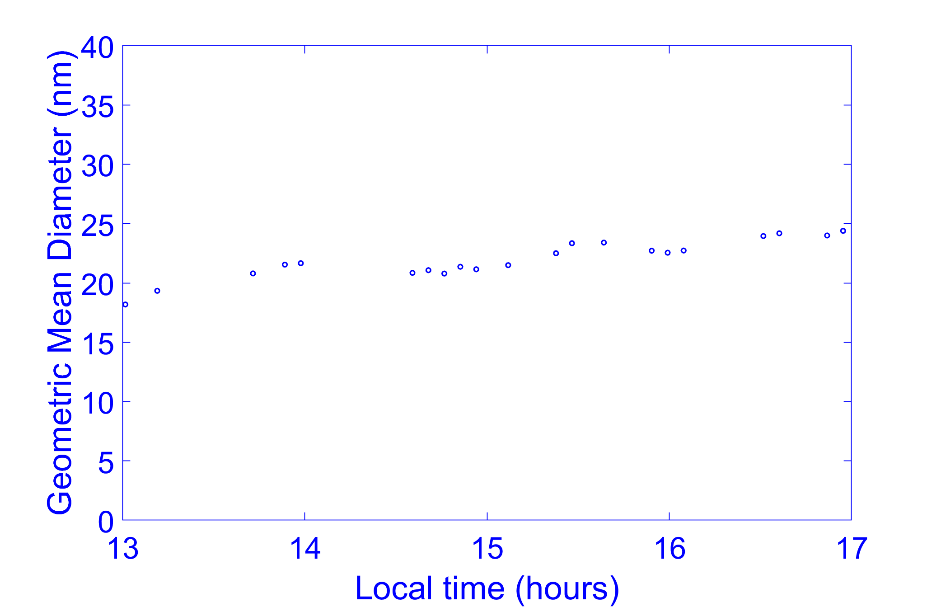


C

Supporting Information Figure 7. A) Contour plot of particle number size distribution, B) nucleation mode particle number concentration, and C) the geometrical mean diameter of the nucleation mode particles for July 15, 2021. The black circle represents the appearance of nucleation mode particles and their increase in concentration, while the red circle represents the NPF event, formation, and growth of freshly formed particles.

From Supporting Information Figure 7 B, it is evident that the nucleation mode particles appear and increase in concentration around 12 h (black circle). After the formation or appearance of the nucleation mode particles, they underwent growth (Supporting Information Figure 7 C, and the red circle in Supporting Information Figure 7A). Such behavior fits into the definition of NPF by the Supporting Information Figure 3 classification scheme. However, it is important to note that the duration and strength of this process also exhibit characteristics of a more localized event.

*July 17, 2021*


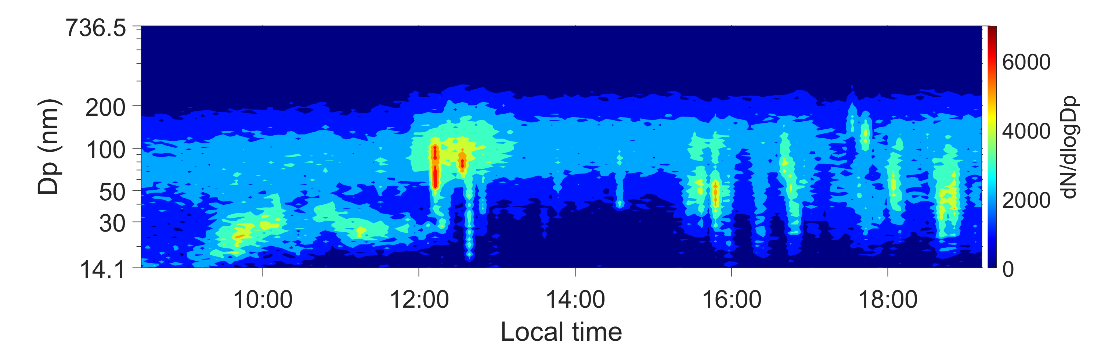


A


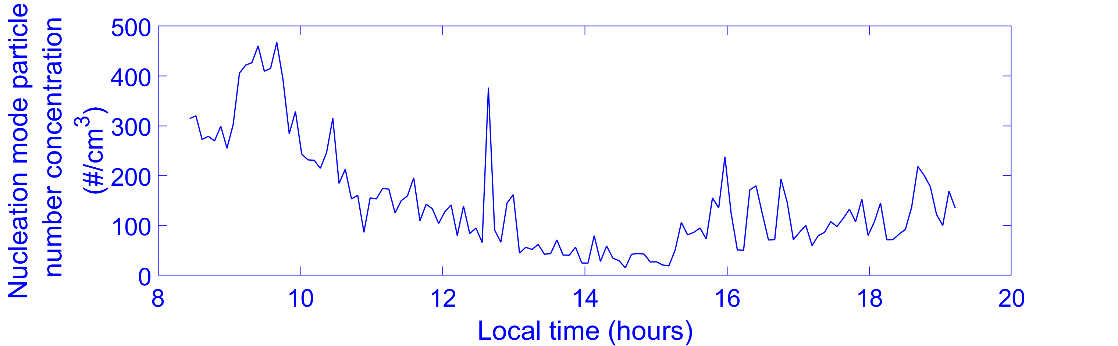


B


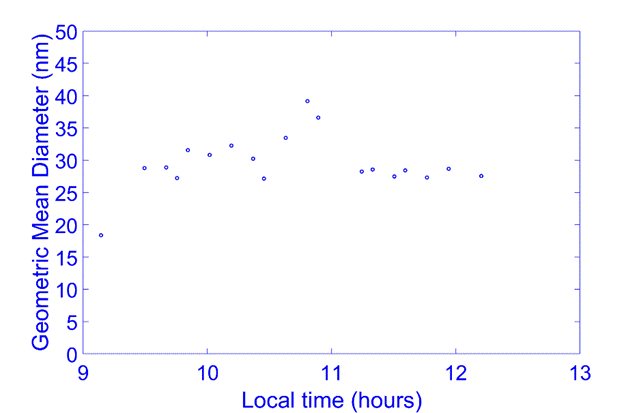


C

Supporting Information Figure 8. A) Contour plot of particle number size distribution, B) nucleation mode particle number concentration, and C) the geometrical mean diameter of the nucleation mode particles for July 17, 2021. The black circle represents the increase of nucleation mode particle number concentration while the red circle shows the NPF event, formation, and growth of freshly-formed particles.

Supporting Information Figure 8 depicts the increase in the number concentration of nucleation mode particles (black circle) and their subsequent growth (red circle). Additionally, Supporting Information Figure 8C shows that during the July 17, 2023 event, the GMD of the nucleation mode particles evolved over time. By the Supporting Information Figure 3 classification scheme, this event can be classified as an NPF event. Furthermore, because the event is short, it is probably more of a local character.

**Failed events**

1. **Quasi events**

*July 7, 2021*

The July 7, 2021, event demonstrated the appearance of nucleation mode particles (black circle in Supporting Information Figure 9 B). However, the substantial variation in the GMD of these particles, partly attributed to primary sources (as indicated by the spikes in Supporting Information Figure 9A – white circles), introduces ambiguity in assessing any actual growth. Due to the absence of clear and quantifiable signs of growth in the mode diameter, it is reasonable to categorize this event as an 'undefined' day.

*
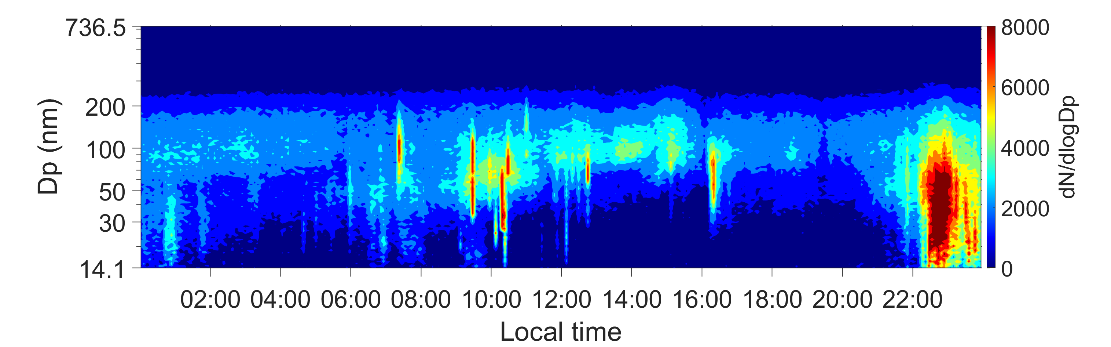
*

A

*
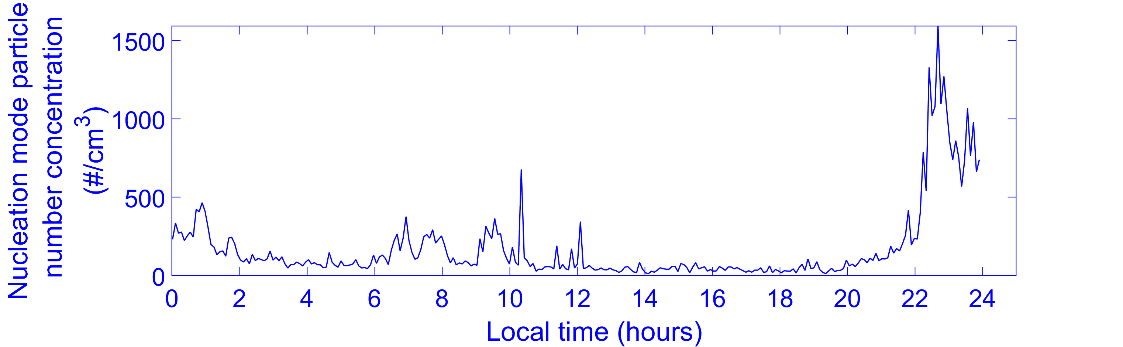
*

B

Supporting Information Figure 9. A) Contour plot of particle number size distribution and B) nucleation mode particle number concentration for July 7, 2021. The black circle represents the increase in nucleation mode particle number concentration, while the white circles represent the contribution of primary sources.

However, referring to the classification proposed by Buenrostro et al. (2009) [[5](#_ENREF_5)], which introduces an alternative classification for undefined days, we find that July 7, 2021, falls under the classification of a quasi-event. According to Buenrostro et al. (2009) [[5](#_ENREF_5)], a quasi-event involves the emergence of a new nucleation mode (Supporting information Figure 9 B- black circle) particles that do not exhibit distinct growth, and this mode should persist for less than one hour or meet both criteria simultaneously.

*July 12, 2021*

Supporting Information Figure 10 presents the size distribution, nucleation mode number concentration, and BC concentration for July 12, 2021. The nucleation mode particles emerge between 10 and 12 hours (black circle) without showing growth. This pattern is consistent with the criteria for a quasi-event as defined earlier.

*
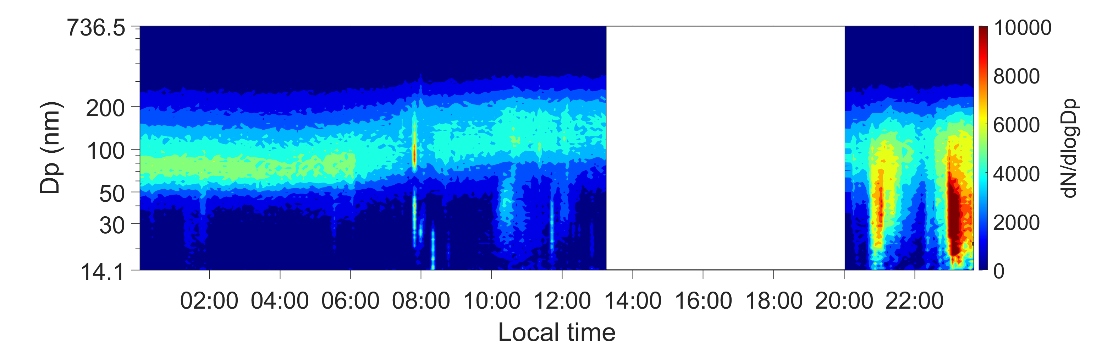
*

A

*
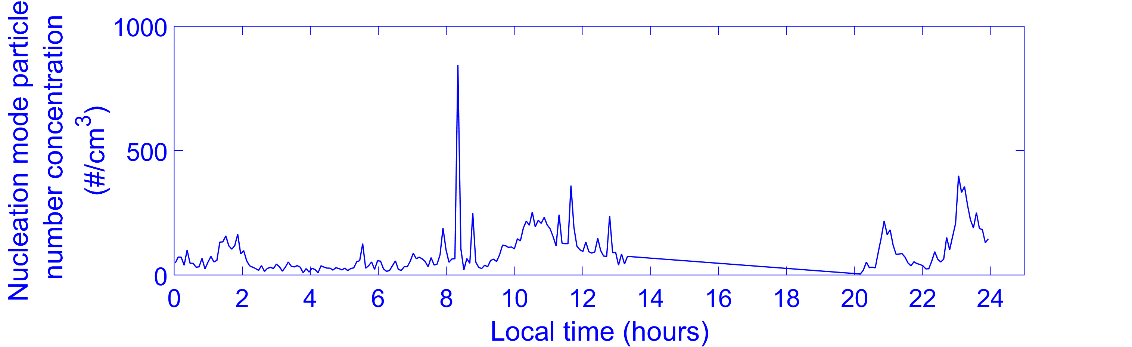
*

B

*
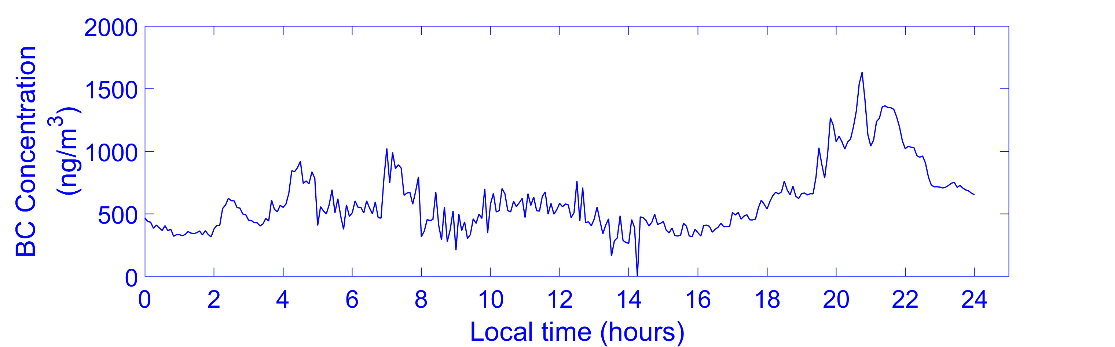
*

C

Supporting Information Figure 10. A) Contour plot of particle number size distribution , B) nucleation mode particle number concentration, and C) black carbon concertation for for July 12, 2021. The black circle represents the nucleation mode particles' appearance, while the blue circle shows the black carbon concentration at the same time interval as nucleation mode particles appear.

While the nucleation mode particles may be associated with BC emissions, the prolonged persistence of these particles for more than 1 hour, without prior observation and with no signs of growth, resembles a quasi-event, as defined by Buenrostro et al. (2009).

*July 13, 2021*

Regarding July 13, 2021, the evidence presented below (Supporting Information Figure 11 A, B, and C) allows us to confidently assert that this event can be classified as a quasi-event. Supporting Information Figure 11 A and B demonstrate that the nucleation mode particles appeared between 10 and 14 hours (black circle), and the mode did not exhibit growth characteristics. Furthermore, during the appearance of nucleation mode particles, the BC concentration did not increase (blue circle). However, by once again employing the classification scheme proposed by Buenrostro et al. (2009) and considering the results presented in Supporting Information Figure 11, it becomes evident that the event on July 13 aligns with the criteria for quasi-events.


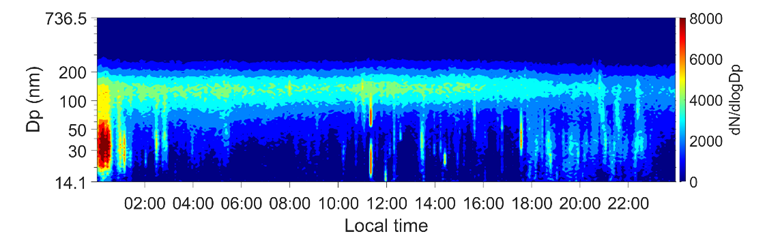


A


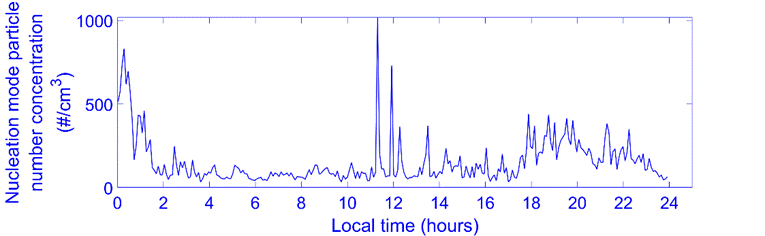


B


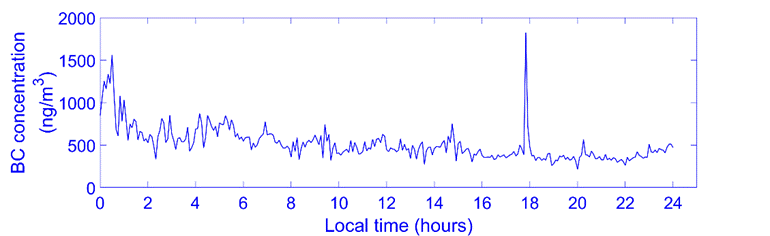


C

Supporting Information Figure 11. A) Contour plot of particle number size distribution, B) nucleation mode particle number concentration, and C) black carbon concentration for July 13, 2021.

1. **Tailed event**

*July 11, 2021*

*
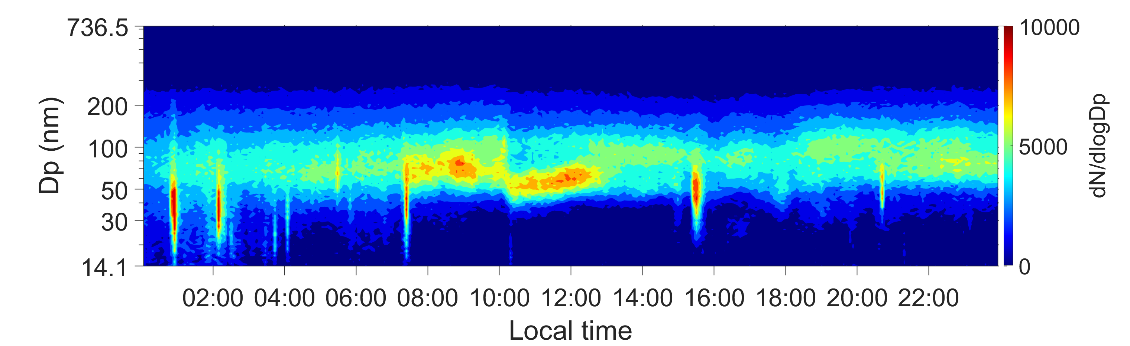
*

A

*
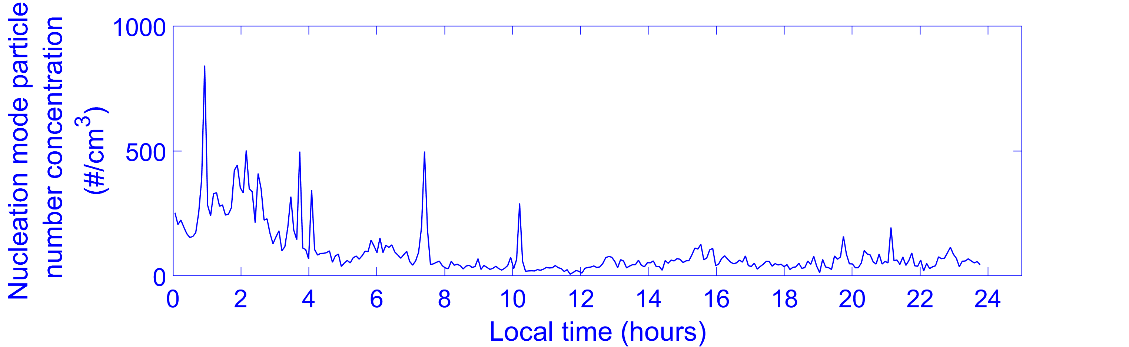
*

B

*
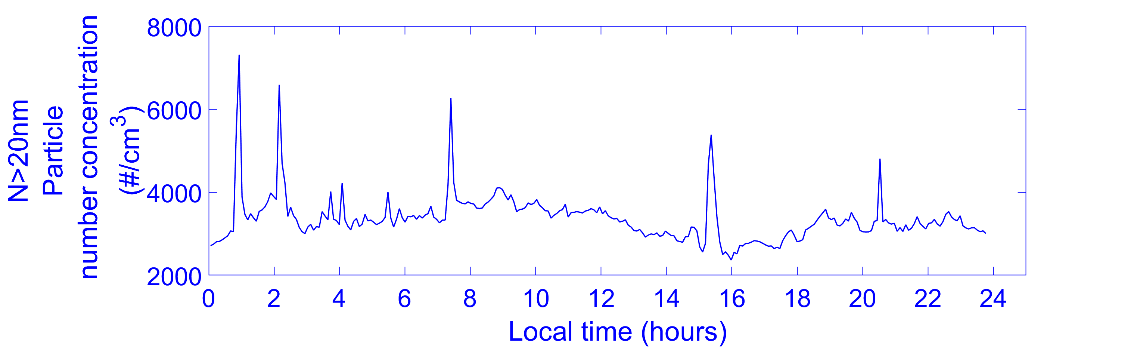
*

C

*
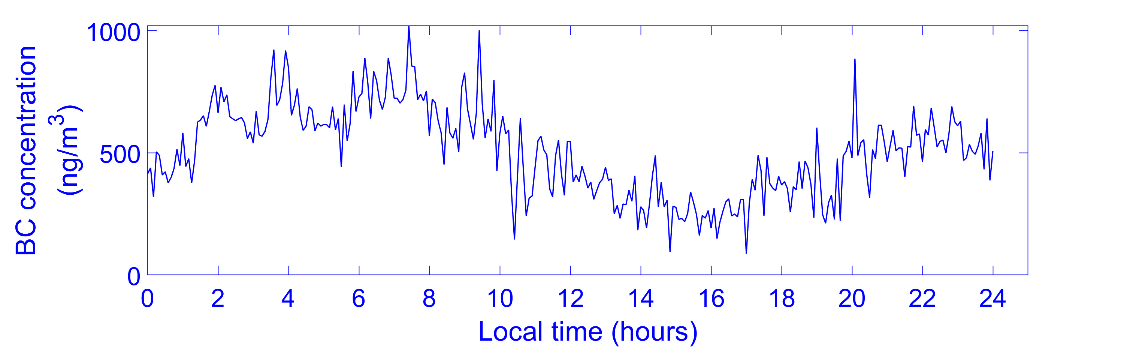
*

D

Supporting Information Figure 12. A) Contour plot of particle number size distributions B) nucleation mode particle number concentration, C) particle number concentration of particle greater than 20 nm (Aitken mode), and D) black carbon concentration for July 11, 2021. The red circle in Supporting Information Figure 12 A and C represents the appearance of a new mode of particle greater than 20 nm. The red circle shows the tail event.

Buenrostro et al. (2009) [[5](#_ENREF_5)] define a tail event as a day where the latter half exhibits the growth of presumably newly formed nucleation mode particles or the late-evening growth of new particles. This phenomenon suggests that the new particles were formed at a different location and transported to the measurement site. The classification criteria for a tail event include the appearance of a new mode at particle diameters greater than 10 nm, followed by a growth that persists for several hours.

Supporting Information Figure 12 A and C shows that on July 11, 2021, a particle mode greater than 20 nm (Aitken mode) appeared between 10 and 13 h (red circle). Moreover, Supporting Information Figure 14 shows that the mode of particles is growing for several hours.

*
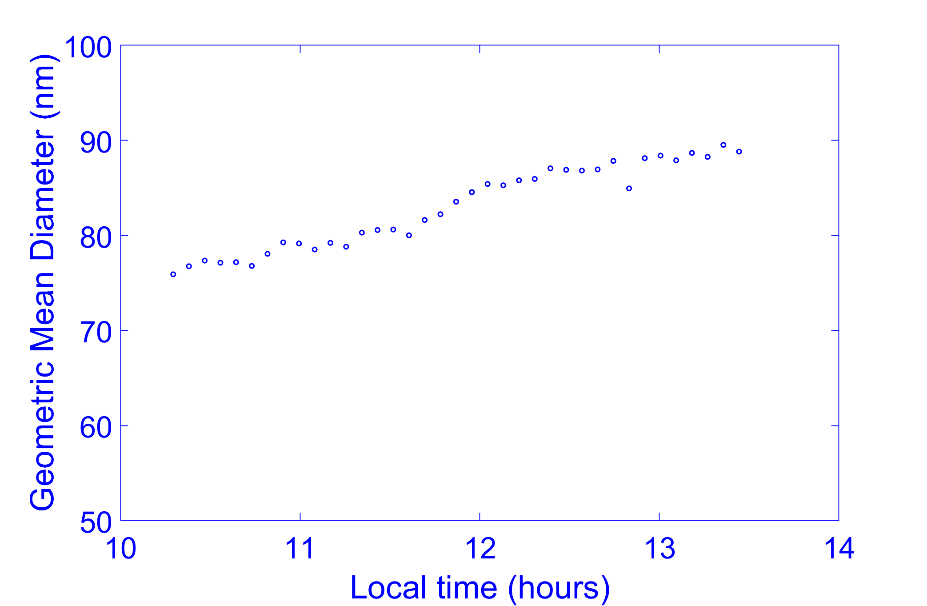
*

Supporting Information Figure 13. The geometric mean diameter of the particle mode is greater than 20 nm (Aitken mode) for July 11, 2021.

Supporting Information Figure 2A shows that the tailed event became evident at approximately 10:00 a.m., coinciding with a notable shift in wind direction and speed (Supporting Information Figure 14). Around 8:00 a.m., the wind direction transitioned from an easterly direction to the northwest (NW). The highest wind speed was recorded when the wind originated from the west (W) around 10:00 a.m. Subsequently, the tailed event was observed after the wind direction changed to the W (Adriatic Sea area). Considering that the tailed event is predicated on the assumption that the particle formation process initiates elsewhere and that the growing particle population is transported to the measurement station, the observed changes in wind direction and speed, along with the tailed event, suggest that the Adriatic Sea can serve as a precursor source for NPF. However, we cannot dismiss the possibility of vertical particle transport, especially considering the well-documented variations in the mixing boundary layer during the summer.


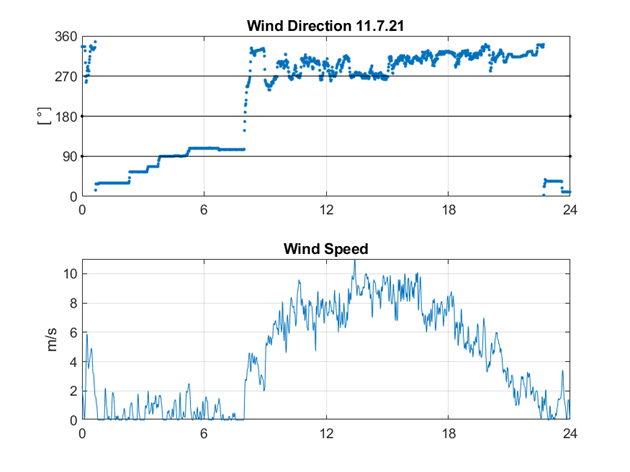


Supporting Information Figure 14. The Metrogram shows wind speed and direction at each time on July 11^th^.

Based on this definition and the findings in Supporting Information Figure 12 A, B, Supporting Information Figure 13, and Supporting Information Figure 14, it is evident that the event on July 11, 2021, conforms to the definition of a tail event.

It should be emphasized that on July 6, the particles were probably also formed at another location and transported to the measurement site. However, July 6 was classified as an NPF event day, not as a tailed event. The main distinction between July 6 and July 11 is that on July 6, nucleation mode particles appeared and grew, whereas on July 11, no nucleation mode particles were observed; instead, Aitken mode particles grew. Consequently, July 11 is classified as a tailed event, while July 6 is categorized as an NPF event day, according to Buenrostro et al. (2009).

**Pollution-related concentration peaks**

*July 5, 2021*

Supporting Information Figure 15 A displays a contour plot of the particle size distribution obtained on July 5, 2021. Upon visual inspection, particles in the nucleation mode observed at 17:30 appeared to grow to 40 nm. Even when considering the geometric mean diameter, the diameter appears to increase over time (Supporting Information Figure 16), suggesting the possibility of an NPF event. On closer examination (Supporting Information Figure 15B), it becomes evident that the mode observed at 17:30 does not persist for more than 1 hour. Additionally, the 20 nm mode particles are likely unrelated to the 40 nm mode particles. The concentration peaks of BC align well with the particle number concentration (Supporting Information Figure 15B), indicating that these particles are probably of primary origin.

*
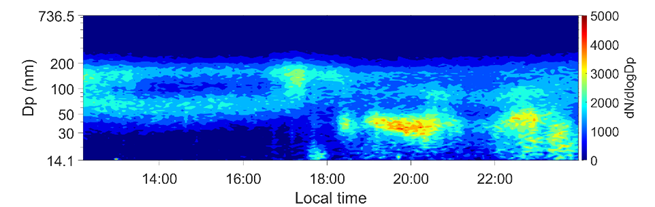
*

A

*
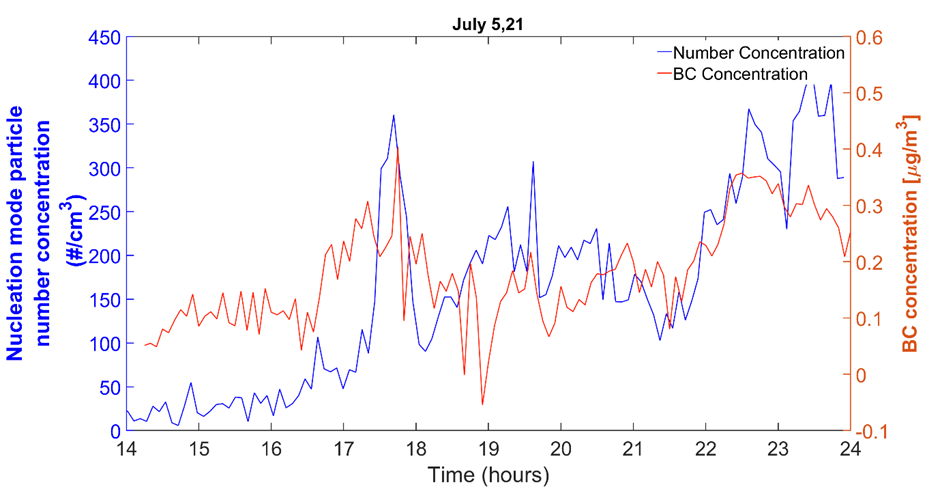
*

B

Supporting Information Figure 15. Contour plot of particle number size distribution and B) nucleation mode particle number concentration and the BC concentration for July 5, 2021.

Furthermore, the modeled backward trajectories indicate that after 18:00 h, the trajectories shifted from being predominantly influenced by the Adriatic Sea to being more influenced by land. Supporting Information Figure 17 illustrates that at 15:00 h, the modeled backward trajectories are situated 46% of the time over the Adriatic Sea. By 18:00 h, a noticeable change in trajectory direction is evident, with only 12% of the time spent over the Adriatic Sea. Finally, by 21:00 h, the backward trajectories show 0% presence over the Adriatic Sea. The presence of the 20 nm particle mode may be attributed to NPF as the air masses are more influenced by marine air at its appearance. However, as the trajectories change direction and become more influenced by continental air, the NPF process (especially the growth) is disrupted, and more land-origin particles influence the measurement.

Supporting Information Figure 16. The evolution geometric mean diameter of the nucleation mode particles for July 5, 2021.

Considering that the peaks in 40 nm particle number concentration coincide with the BC, such an event can be classified as a pollution-related concentration peak (as defined by Buenrostro et al., 2009 [[5](#_ENREF_5)])


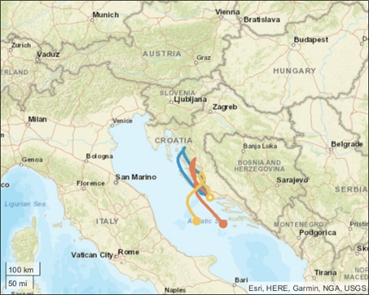

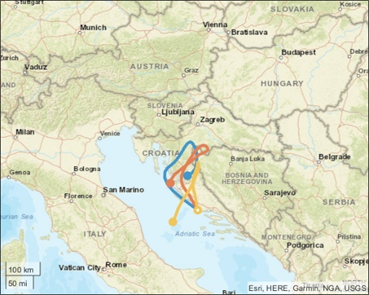


B

A


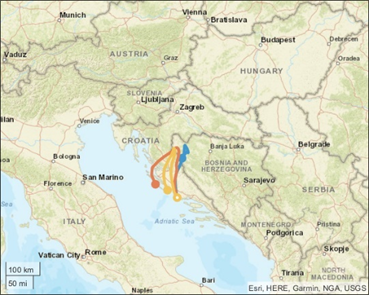


C

Supporting Information Figure 17. Air-mass backward trajectories during July 5, 2021, for A) 15:00 h, B) 18:00 h and C) 21:00.

**Non-events**

*July 8, 2021*

Supporting Information Figure 18 shows the size distribution, nucleation mode particle number concentration, and BC concentration for July 8, 2021. It can be noticed that during the whole day, there is no appearance of nucleation mode particles. Spikes in Supporting Information Figure 18A (indicated by white circles) represent primary sources (BC emissions), likely responsible for the sharp peaks observed in the nucleation mode particles. Furthermore, Supporting Information Figure 18A demonstrates the absence of distinct particle growth**.** Moreover, Supporting Information Figure 18B shows minimal variation in particle number concentration, except for the spikes originating from the primary BC source.

*
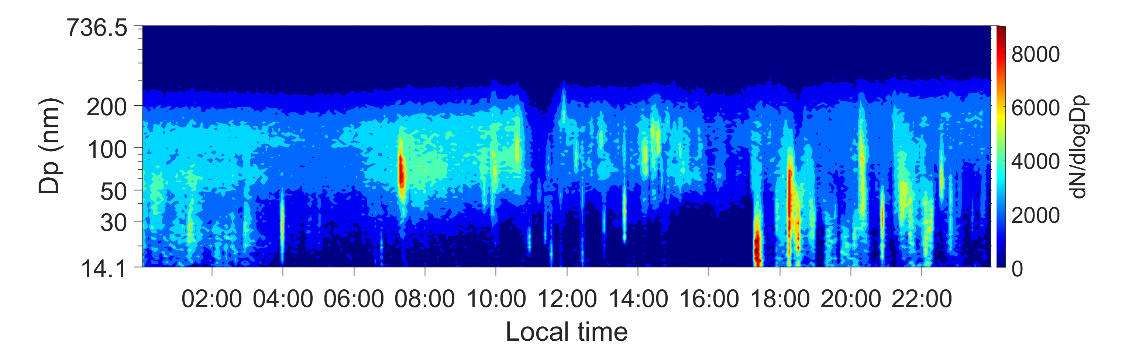
*

A

*
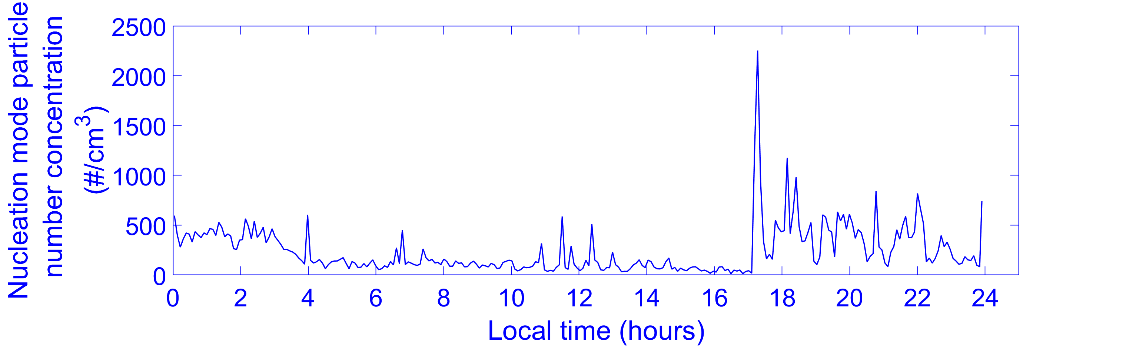
*

B

*
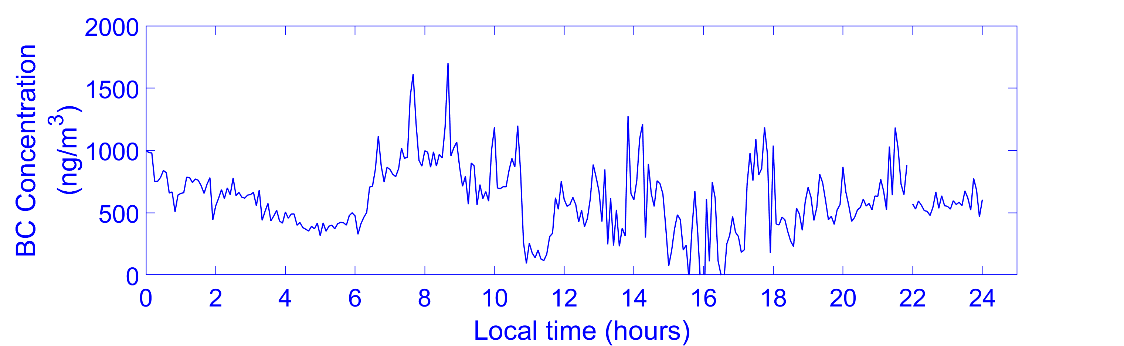
*

C

Supporting Information Figure 18. A) Contour plot of particle number size distributions, B) nucleation mode particle number concentration, and C) black carbon concentration for July 8, 2021. The black circle indicates the black carbon concentration increase. The white circles represent the spikes caused by black carbon emissions.

Considering all these observations and the adjusted classification scheme (illustrated in Supporting Information Figure 3B), it is evident that the event on July 8, 2021, should be classified as a non-event day.

*July 10, 2021*

While Supporting Information Figures 19 B and C reveal certain fluctuations in the modes (nucleation and Aitken), the absence of the data prevents us from confidently asserting a distinct pattern within these fluctuations. Furthermore, the BC concentration illustrated in Supporting Information Figure 19 D does not exhibit any noticeable alignment with the particle number concentration (nucleation or Aitken). Due to these limitations, we find it more reasonable to classify July 10, 2021, as a non-event day rather than an ultra-fine mode or pollution-related peak following the classification scheme proposed by Buenrostro et al. (2009) [[5](#_ENREF_5)].

**
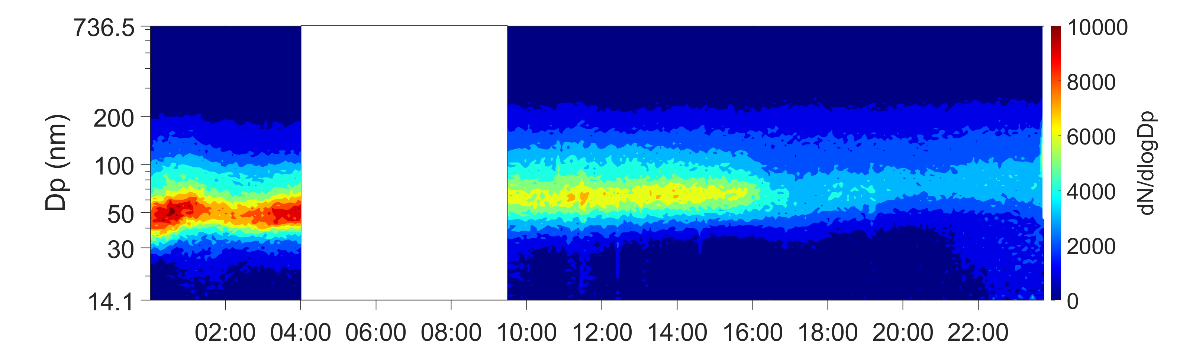
**

A

*
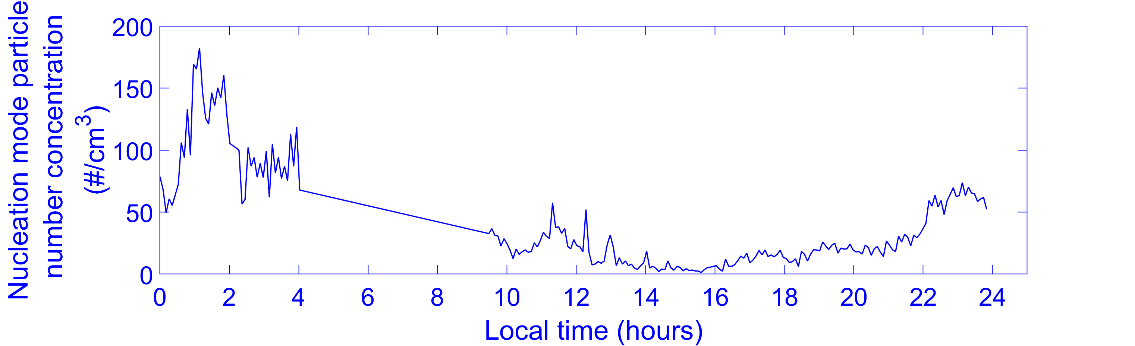
*

B

*
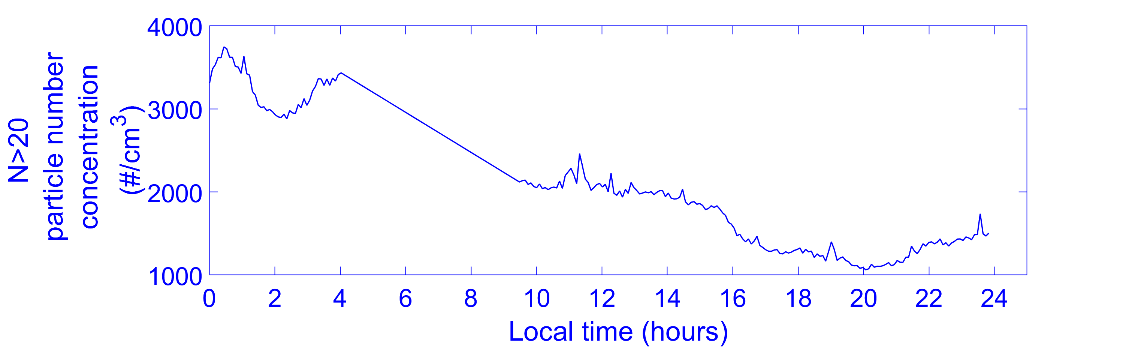
*

C

*
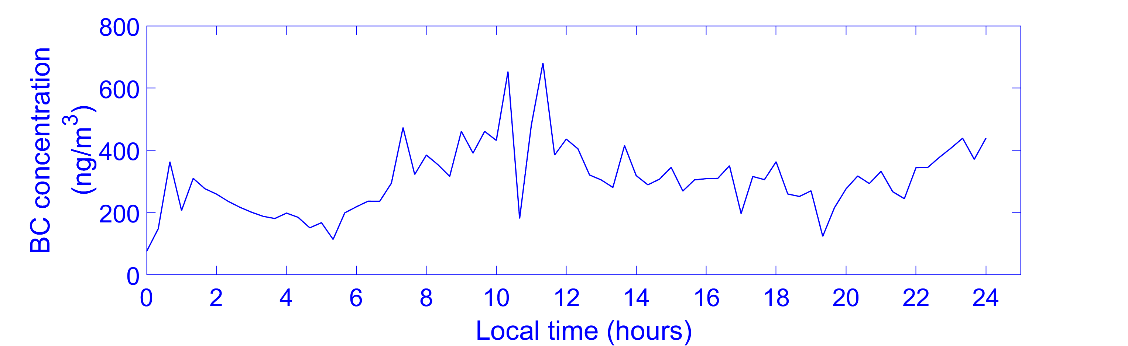
*

D

Supporting Information Figure 19. A) Contour plot of particle number size distributions, B) nucleation mode particle number concentration, C) particle number concentration of particle greater than 20 nm (Aitken mode), and D) black carbon concentration for July 10, 2021. The yellow circles represent the missing data from SMPS.

*July 16, 2021*

*
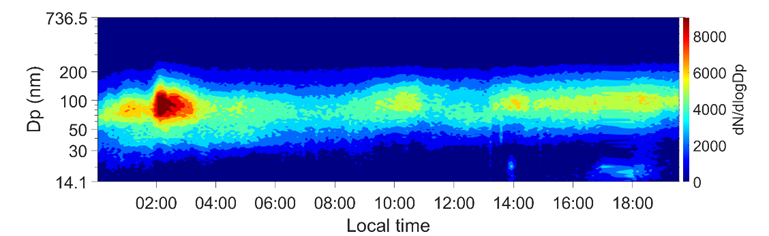
*

A

**
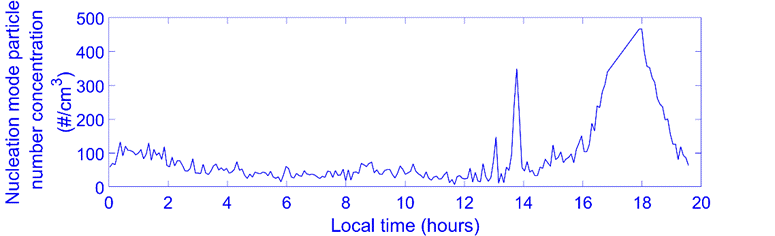
***
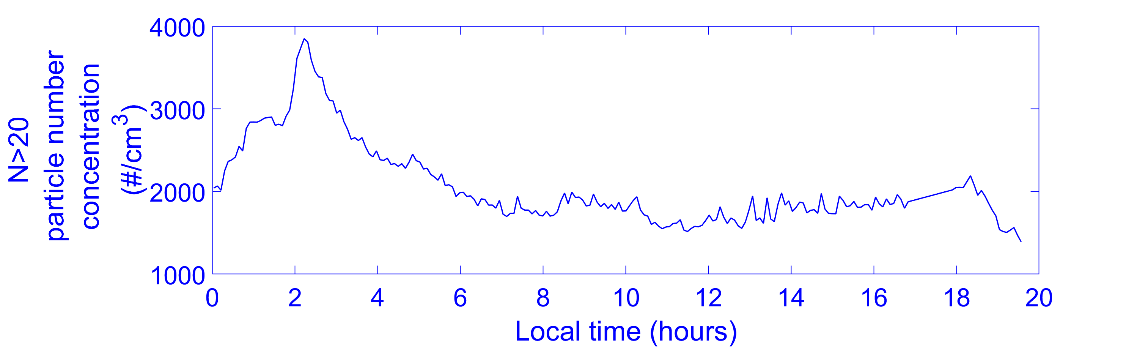
*

C

B

*
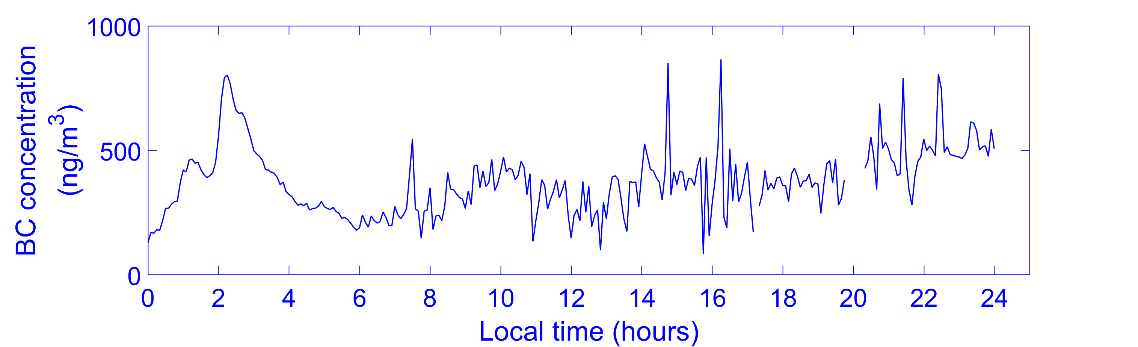
*

D

Supporting Information Figure 20. A) Contour plot of particle number size distributions, B) nucleation mode particle number concentration, C) particle number concentration of particle greater than 20 nm (Aitken mode), and D) black carbon concentration for July 16, 2021.

In Supporting Information Figure 20, it is evident that although Aitken mode particles exhibit a peak around 2 h (black circle), corresponding to an increase in BC concentration, the remaining portions of the day show minimal fluctuations in Aitken mode particle number concentration (Supporting Information Figure 20 C). Similar to the examples discussed earlier, the utilization of the classification scheme introduced by Buenrostro et al. (2009) [[5](#_ENREF_5)] leads us to classify this particular event on July 16 as a non-event day. Although the rise in Aitken mode particle number concentration and an increase in BC might initially suggest a pollution-related peak, we believe the elevation in BC levels is more likely attributed to a local temperature inversion phenomenon. This occurrence is commonly observed during summer nights.

**Apparent formation rate, growth rate, condensation sink and the vapor concentration calculation**

**Apparent formation rate.** The apparent formation rate (*J*_15_) of new particles of size *d*_p_ (15 nm) was calculated as follows:

$\frac{\Delta N_{D,D_{max}}}{\Delta t}\left| \right._{observed}=J_{D}-\frac{\Delta N_{D,D_{max}}}{\Delta t}\left| \right._{self-coag}-\frac{\Delta N_{D,D_{max}}}{\Delta t}\left| \right._{coag-scav}-\frac{\Delta N_{D,D_{max}}}{\Delta t}\left| \right._{transport}$ (S1)

In equation (S1), *N*_D_, _Dmax_ represents the total particle number concentration in the size range [*D*, *D*_max_], where *D*_max_ is the maximum size critical clusters can reach due to their growth during Δt. The first term on the right-hand side (*J*_D_) of the equation is the observed change in *N*_D,Dmax_ during Δt, which can be obtained from particle size distribution or number concentration measurements. The second and third terms represent the loss of particles in the size range [*D*, *D*_max_] due to self-coagulation and coagulation-scavenging to larger preexisting particles, respectively. The last term represents the influence of air mass transport on *N*_D,Dmax_, which may become necessary in fixed-site measurements exposed to inhomogeneous air masses. However, in areas such as Rogoznica, where the effect of transport is minor, equation (S1) can be simplified:

$J_{D}\approx\frac{\Delta N_{D,D_{max}}}{\Delta t}\left| \right._{observed}-CoagS_{dp}\cdot N_{dp}$ (S2)

In S2, the CoagS*_dp_* is the coagulation sink of the particles in the size range [D, D*_max_*], and *N_dp_* represents the particle number concentration in the size range [D, D*_max_*]. The overall term *CoagS_dp_ N_dp_* represents the coagulation losses due to larger particles (scavenging coagulation).

Equation (S2) is the most common formula for estimating an atmospheric particle's apparent formation rate. Kulmala et al. (2004, 2012) [[8](#_ENREF_9), [10](#_ENREF_11)] pointed out that equation (S2) is an excellent approximation to equation (S1), especially in relatively homogeneous air masses. It's crucial to emphasize that this approach allows for the measurement of only the apparent formation rate. Kerminen et al. (2001) [[11](#_ENREF_12)] demonstrated a positive correlation between the apparent formation rate and the actual nucleation rate, with consistently lower values observed for the apparent formation rate.

**Growth rate.** The particle growth rate during the identified events was calculated using the log-normal mode fitting method, as described by Kulmala et al. (2012) [[10](#_ENREF_11)]. In this method, each discrete size distribution during the event obtained by SMPS is fitted with 2 – 4 log-normal modes, as shown in equation (S3).

$\frac{dN}{d(logD_{p})}=\sum_{i=1}^{n=4} \frac{N_{tot,i}}{\sqrt{2\pi}log(\sigma_{g,i)}}exp\left[ \left. -\frac{(\log D_{p}-log(D_{pg,i})^{2}}{2log^{2}(\sigma_{g,i})} \right] \right.$ (S3)

In equation (S3), *D*_p_ represents the particle diameter, and the three parameters needed to identify individual modes are the total number concentration (*N*_tot_), the geometric variance (*σ*_g_^2^), and the geometric mean diameter (*D*_pg_). The maximum number of possible individual modes is represented by *n*. The least-squares-fitting method was used to obtain the best fit of the measured particle number size distribution to the multi-log-normal distribution function. The representative particle size for each time step was defined as the geometrical mean of each fitted mode. Additionally, the representative particle size was plotted as a function of time to calculate an average overall growth rate (equation S4).

$GR= \frac{dD_{p}}{dt}$ (S4)

To automatize the calculation of large sets of size distribution, we upgraded the method with an in-house algorithm developed in MATLAB. Usually, the data obtained by SMPS often consist of a discrete distribution with superimposed modes. To accurately define and follow the evolution of particle nucleation mode throughout time, we developed a custom peak deconvolution algorithm that considers the log-normal distribution of the data and noise. The first step involves pre-processing using a median filter (equation S5) followed by Gaussian smoothing (equation S6) to obtain the mode position.

$y\left( t \right)=median(x\left( \frac{t-T}{2} \right),x\left( t-T_{1}+1 \right),\ldots,x\left( t \right),\ldots,x\left( \frac{t+T}{2} \right))$ (S5)

$G\left( x \right)= \frac{1}{\sqrt{2\pi\sigma^{2}}}e^{-\frac{x^{2}}{2\sigma^{2}}}$ (S6)

In equation (S5), *x* represents the input signal, and *y* represents the filtered output signal. *T* is the length of the median filter, and *T*_1_ is a parameter that specifies the center of the median filter. The window size for each median filter is adjusted to account for the log-normal distribution data. To remove the possible noise, we implemented the Gaussian smoothing function (equation S6), a low-pass filter used to remove noise from a signal by convolving the signal with a Gaussian kernel. The Gaussian kernel is a bell-shaped curve centered around the origin with a standard deviation σ, which controls the curve's width. In equation (S6), *x* represents the distance from the center of the kernel, and *G(x)* represents the weight assigned to the value of the signal at that distance. The terms $\frac{1}{\sqrt{2\pi\sigma^{2}}}$ and $e^{-\frac{x^{2}}{2\sigma^{2}}}$ represent the normalization factor and shape of the Gaussian kernel, respectively. The peak position location (center) is an initial parameter for peak deconvolution on the original data. Finally, the deconvolution quality with one or more modes was carried out using Akaik and Bayesian information criteria. Such a hybrid approach allowed us to obtain overlapping particle distribution without any data manipulation that could misrepresent the true nature of the collected data.

**Instantaneous Growth rate (semi-empirical 1^st^ derivative method).** The obtained geometrical mean diameter for the nucleation mode was followed throughout the duration of the event (Supporting Information Figure 21). The observed data for all events were fitted with a third-order polynomial function, which provided a satisfactory determination factor. When taking the first derivative of this continuous function, it yields the rate of increase of the geometric mean diameter (GDM) at each instance of time. Such an approach allowed us to determine the instantaneous overall growth rate. The overall growth rate consists of self-coagulation, scavenging-coagulation, and condensation growth rates. Given that the individual coagulation growth rates can be determined for each time point using the principles of Brownian motion theory and the measured size distribution, the instantaneous condensation growth rate is then calculated by subtracting both coagulation growth rates from the overall growth rate at each time point.


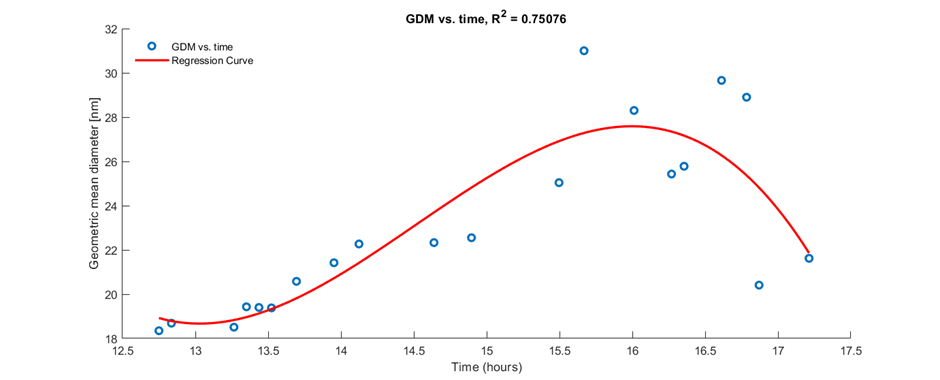


Supporting Information Figure 21. The calculated instantaneous growth rate for the observed particle size distribution in Rogoznica on July 14, 2021. Geometric mean diameter evolution during NPF event day and the third-order polynomic regression curve.

**Condensation sink (CS).** The CS represents the average rate at which non-volatile vapor-phase molecules will condense into the entirely aerosol particle population surfaces and was calculated from the size distribution data as follows:

$CS=2\pi D\sum_{dp} \beta_{m,dp}d_{p}N_{dp}$ (S7)

Here, *β*_m,dp_ represents the transition-regime correction factor for the condensation of a vapor onto a particle with a diameter of *dp*. The vapor diffusion coefficient (*D)* and the continuous particle number size distribution function (*N*_dp_) were used to calculate the condensation sink. The diffusion coefficient for each vapor was calculated using a semi-empirical prediction for the binary diffusion coefficient (equation S8).

$D_{AB}=\frac{{0.00143T}^{1.75}}{P\sqrt{M_{AB}}\cdot\left[ \sqrt[3]{(\sum v)_{A}}+\sqrt[3]{(\sum v)_{B}} \right]{}^{2}}$ (S8)

*D*_AB_ is the binary diffusion coefficient of species A and B, *T* is the temperature, *P* is the pressure, *M*_AB_ is the molecular weights of species A and B, and *Σ*_v_ is calculated for each component by summing its atomic diffusion volumes.

**Vapor concentration.** The vapor concentration was calculated from the differential form of the growth rate equation:

$\frac{{dd}_{p}}{dt}=\frac{4m_{v}\beta_{m}DC}{d_{p}\rho}$ (S9)

Here, *dp* is particle diameter, *m*_v_ is the molecular mass of condensable vapors, *D* is the diffusion coefficient, and *ρ* is the particle density. We used the previously calculated instantaneous condensation growth rate (dd_p_/dt) to determine the vapor concentration.

**Air mass back trajectories.** The National Oceanic and Atmospheric Administration (NOAA) HYSPLIT model was used to compute air mass backward trajectories at a temporal resolution of 1 hour for a 72-hour duration33. The model utilized meteorological data from the Global Forecast System (GFS) model, which has a spatial resolution of 0.25°. The backward trajectories were calculated for three heights, i.e., 100 m, 500 m, and 1000 m. We used the NOAA HYSPLIT model back trajectories and terrene height data to distinguish between marine and land-influenced air masses. Backward trajectories originating from the height of 0 meters were considered to represent marine air masses, while those originating from other heights were defined as land-influenced air masses. We used a custom MATLAB code to determine the time spent by each trajectory over the sea surface by counting the instances where the trajectory height was at 0 meters. Based on this information, we calculated the percentage of time spent over the sea surface for each trajectory.

To determine the duration of the trajectories' crossing of the Adriatic Sea, we created a mask for this region using the 'inpolygon' function (in MATLAB). This function tests if the coordinates of the trajectories are within the boundaries of the polygon of the Adriatic. This way, we can isolate the specific segments of the trajectories relevant to the Adriatic Sea and calculate the time spent over its surface.

**Meteorological data**. Meteorological parameters were collected from a weather station installed at ground level and located 400 m from the sampling site. Temperature, relative humidity, wind speed, wind direction, and solar radiation were used for further processing. All data were processed using Excel, Origin, and MATLAB.

**Tidal signal**. The tidal signal in Rogoznica was reconstructed using water level measurements recorded with Sensus Ultra Dive Data recorders (ReefNet). These measurements were filtered using a two-step process, which acted as a band-pass filter. First, a moving average filter with a cut-off frequency of one hour was applied to remove shorter-periodic oscillations (such as bay seiches) from the signal. Then, another moving average filter with a cut-off frequency of three days was used to extract longer oscillations caused by mesoscale disturbances. This filtered signal was subtracted from the entire series. The resulting water level signal was used for tidal harmonics analysis, which was performed using the t_tide package. The data from five extracted constituents were used in tidal signal reconstruction based on prior knowledge of the Adriatic Sea tidal regime. A signal-to-noise ratio criterion greater than two was used during the analysis.

**Physical Characterization of NPF Process in Rogoznica**

*Apparent formation rate (J_15_)*

We report the average *J*_15_ and growth rate for each event day in Table 1. The Adriatic Sea area has a considerably lower particle *J*_15_ than other coastal areas, such as Mace Head, which ranges from 10^5^ to 10^6^ cm^-3^ s^-1^. However, Mace Head is in the open ocean, while the Adriatic Sea is semi-closed. The *J*_15_ values observed were similar to those found in the boreal forests of southern Finland and the Antarctica peninsula [[7](#_ENREF_8), [12](#_ENREF_13)]. The low *J*_15_ in the Adriatic Sea during summer could be due to the dilution of emitted vapors caused by the increase in mixed layer height [[13](#_ENREF_14)], rather than cluster removal by coagulation [[7](#_ENREF_8)]. Although the highest *J*_15_ value was observed when the wind direction was from the southwest, no apparent correlation was found between meteorological and particle parameters and *J*_15_. Low *J*_15_ could also result from air mass inhomogeneity and other processes altering size distribution at lower measurable particle sizes.

*Growth rate (GR)*

Using the log-normal fitting mode method, we calculated the overall average GR (Table 1) for each observed event day [[10](#_ENREF_11)]. We upgraded this method's version to improve efficiency. We used the Brownian motion theory [[14](#_ENREF_15), [15](#_ENREF_16)] to calculate the coagulation GR. The condensation GR was obtained by subtracting the average coagulation GR from the overall GR. However, this approach only yields the average condensation GR. In this study, we introduce the semi-empirical first derivative method to calculate the instantaneous overall GR, which enables calculating the condensation GR at any given time (see Methods). This method makes it feasible to determine the GR for individual particle modes and identify any evaporation occurrence.

**Shrinkage effect**

Specific atmospheric conditions refer to changes in the mixing layer depth, which can occur when a polluted air mass mixes with cleaner air. This mixing can cause semi-volatile compounds to undergo re-partitioning, resulting in particle shrinkage. The shrinkage events of NPF observed in Rogoznica are less likely connected to the mixing of atmospheric layers. For example, evaporation was dominant during the July 14, 2021 event (as shown in Figure 2 and Figure 4). It was observed that mixing occurred before the NPF event (black dashed circle in Figure 2A), as the particle size distribution was unimodal until 9 a.m. After that, it remained unimodal but with a smaller particle mode diameter. This indicates that the background particles indeed underwent evaporation due to air dilution. At noon, a burst of new particles was observed, followed by a growth event that caused the particle size distribution to shift from unimodal to bimodal (Figure 2A-red dashed circle). The second mode (nucleation mode) grew in diameter with the bimodal distribution. This growth was primarily driven by condensation, which dominated the GR of the newly formed particles (see Figure 4). The nucleation mode continued growing in diameter until 4 p.m. when evaporation again occurred (Figure 4). According to recent studies, the particles that grow after nucleation typically contain organic, nitrate, sulfate, and ammonium ions as significant chemical species. Some of these compounds can be semi-volatile [[16](#_ENREF_17)]. However, another study by Kivekas et al. (2016) [[17](#_ENREF_19)] concluded that this explanation requires further investigation, as newly formed particles in their initial stage are expected to consist of species with low or extremely low vapor pressure (as noted by Ehn et al., 2014 [[18](#_ENREF_20)]). It is unclear why SVOCs condense onto particles in the first place. Our explanation, supported by the observation data, is that the presence of SAS facilitates the transfer of such compounds from the gaseous phase to the particle surface.

**Vapor concentration.**

The concentration of vapors is a crucial parameter that triggers the NPF process. To calculate the vapor concentrations required to achieve the observed GR, one can use the analytical tools developed by Kulmala et al. in 1988 [[19](#_ENREF_21)] and 2001 [[15](#_ENREF_16)]. Using the differential form of the equation (Equation S9) with the instantaneous GR allows for determining the concentration for each instance of the GR. Table S3 provides a general pattern of the vapor concentration required to achieve the observed GR.

*Interpretation of July 9, 2021 event*

The July 9, 2021, event is visually different and shows different physical and chemical characteristics compared to other events. Supporting Information Figure 2 shows that only the July 9, 2021 event does not correlate with the tidal effect. Moreover, the calculated backward trajectories indicate that trajectories originating from the lowest height (500 m) traverse over 80% of land areas (Figure 2C). Additionally, these trajectories pass through the Po Valley, which is recognized as a European pollution hotspot. In line with this, the higher measured BC concentration on this day suggests a stronger anthropogenic influence compared to other NPF event days. This specific event can also be distinguished by its duration and strength. Other recognized events have a more local character, small duration, and GR. On the other hand, this event can be categorized as a regional event by all the characteristics. Also, the nucleation mode particles' missing lower limit indicates particle formation started elsewhere. On July 9, 2021, the CS was higher than for other event and non-event days, and the occurrence of NPF under relatively high CS is a characteristic of highly polluted mega cities [[20](#_ENREF_22)]. In addition, chemical analysis reveals the absence of bromine (Br) during the July 9, 2021 event, while the concentration of SO_4_^2−^ is notably higher than for other events. SAS identified on that specific day exhibited a different surface activity nature than other events. All these observations strongly suggest that the nucleation and growth of particles on July 9, 2021, likely have a distinct origin and are governed by a different mechanism.

*Interpretation of July 6, 2021 event*

The July 6, 2021, event exhibits NPF characteristics with nucleation mode particles appearing and growing for several hours. However, interference from primary emitted particles (mainly BC) is noted, superimposing and affecting the appeared nucleation mode particles (see Supporting Information Figure 4). The interaction with primarily emitted particles can also be seen from the calculated coagulation sink and the coagulation growth rate. The July 6, 2021, event shows the highest coagulation sink and coagulation growth rate (see Table S2 and Figure 4), likely due to increased collision with primary emitted particles. Furthermore, on July 6, 2021, the event had the highest average total GR (see Table 1). The more substantial influence of the anthropogenic sources could explain the increase in total GR on this particular day compared to other days. Consistent with this, the modeled backward trajectories (at 500 and 1000 m) indicate a significant influence of land on the air masses (see Supporting Information Figure 25 A) during the July 6, 2021 event. It's important to note that the amount of SAS was highest during this event, and the nature of the presented SAS (measured by NSA parameter) differed from other events. Also, on July 6, 2021, despite the significant anthropogenic influence, the event had characteristics of a rather localized event due to its duration.

Furthermore, Br^-^ in ultrafine mode particles suggests the potential involvement of reactive Br species in oxidizing VOCs and forming LVOCs, which can be responsible for particle growth. The likely source of Br is a biological activity in the Adriatic Sea, as no other anthropogenic sources are anticipated. Moreover, growth is initiated during maximum sun radiation and low tide (see Supporting Information Figure 2), which coincides with the peak of biological activity. The OC content in ultrafine mode particles on July 6, 2021, increased compared to non-event days. This increase can be linked to the oxidation of VOC induced by reactive Br species, leading to the formation of LVOCs (or OOM), which may contribute to particle growth. The highest growth rate can be partially explained by the higher Br^-^ content in ultrafine mode particles, suggesting the presence of a larger quantity of reactive Br species. An essential factor to consider is the anthropogenic influence that results in higher NO_3_^-^ concentration, essential for activating Br species into reactive Br [[21](#_ENREF_23)]. This influence also results in various VOCs, which may differ from those emitted biologically, consequently affecting the growth rate and the nature of the LVOCs (or OOMs).

Surface activity measurements and the NSA parameter may indicate the existence of a distinct growth mechanism. On July 6, 2021, the measured surface activity in ultrafine mode particles was higher, while the hydrophobicity was lower compared to other NPF events, particularly those on July 14 and 15. Also, lower concentrations of carboxylic acids (only oxalate) were present on July 6, 2021. The relative hydrophobicity/hydrophilicity can be inspected from the NSA parameter. By comparing the NSA parameters of highly hydrophobic (Triton-X-100, logP = 4.61, NSA = 1.72) and hydrophilic (fulvic acid, logP = 0.3, NSA = 0.168) compounds with the NSA of the extracted WSOC, we can determine their relative hydrophobicity/hydrophilicity [[22](#_ENREF_24)].


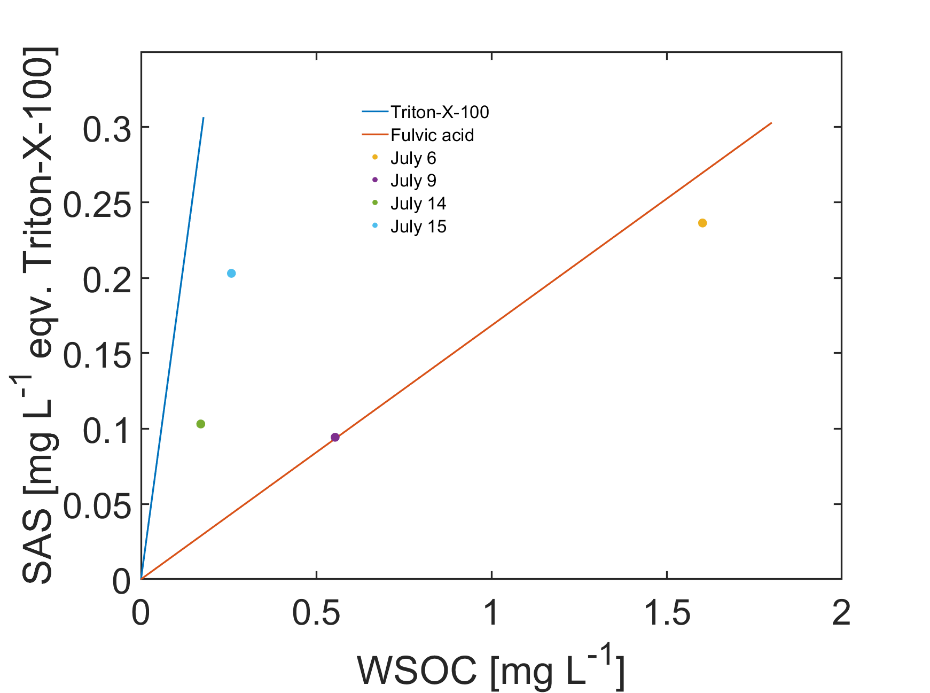


Supporting Information Figure 22. Surface activity and WSOC values obtained from ultrafine mode particles collected during the NPF event days. The points on the graph represent the SAS/WSOC (NSA) for each NPF event day. In contrast, the line represents the corresponding slopes of normalized surface activity (NSA) for the hydrophobic (Triton-X-100) and hydrophilic (fulvic acid) compounds.

Supporting Information Figure 22 reveals ultrafine mode particles from July 6 exhibit greater hydrophilicity than Triton-X-100 and ultrafine mode particles from July 14 and July 15. It has been demonstrated that higher hydrophobicity leads to particle growth up to 30 nm (on July 14 and July 15), whereas lower hydrophobicity results in particle growth up to 100 nm (on July 6 and July 9). This implies that the particle growth mechanism can vary depending on the hydrophobicity or hydrophilicity of the growing particle population. Therefore, different growth mechanisms can be anticipated depending on the nature of the LVOCs (OC or OOMs) involved in the growth. The event on July 9 showed a characteristic of a regional event and more anthropogenic influence, which resulted in lower hydrophobicity of the ultrafine mode particles and the growth up to 100 nm. However, on July 6, the NPF event day was also influenced by anthropogenic sources, which could be responsible for the lower hydrophobicity of the particles.

Lower hydrophobicity indicates that a more significant portion of LVOCs (or OOM) participating in the growth are not surface-active. We cannot delve into the exact mechanism in this context, as it requires more detailed laboratory studies. However, we can speculate that the growth of particles up to 100 nm in the presence of more hydrophilic LVOCs may be attributed to the absence of critical normalized surface activity. As the normalized surface activity increases (as seen for July 14 and 15), the gas-to-particle transfer of not completely oxidized molecules increases [[23](#_ENREF_25)]. The presence of partially oxidized molecules hinders further growth, as they can act as a barrier to the subsequent condensation of LVOCs [[24](#_ENREF_27)]. Conversely, when the normalized surface activity is below the critical values, there is a smaller barrier for condensation to occur, and particles can grow to larger diameters.

Table S1. Average Particle surface area, PM_2.5_, BC, number concentration, temperature, relative humidity, and wind speed data for observational data at Rogoznica.

| **NPF event days** | Particle surface area [µm^2^ cm^-3^] | PM_2.5_  [µg m^-3^] | BC  [µg m^-3^] | Total Particle number concentration (Average nucleation mode number concentration)  [cm^-3^] | Temperature  [°C] | Relative Humidity  [%] | Wind speed  [m/s] |
| --- | --- | --- | --- | --- | --- | --- | --- |
| July 6 | 90.52 | 16.94 | 0.344 | 1917 (276) | 25.87 | 72.44 | 3.61 |
| July 9 | 123.68 | 16.61 | 0.429 | 2923 (374) | 27.74 | 61.37 | 6.75 |
| July 14 | 96.17 | 16.16 | 0.385 | 3177 (675) | 26.84 | 64.29 | 4.91 |
| July 15 | 69.16 | 15.47 | 0.201 | 3230 (634) | 24.60 | 67.14 | 5.84 |
| July 17 | 95.80 | 17.12 | 0.358 | 2436 (426) | 23.96 | 73.87 | 4.85 |
| **Failed events**  *i) Quasi events* |  |  |  |  |  |  |  |
| July 7 | 121.20 | 16.88 | 0.520 | 2816(423) | 26.52 | 70.22 | 2.16 |
| July 12 | 140.55 | 20.34 | 0.690 | 3404 (269) | 25.44 | 74.80 | 2.38 |
| July 13 | 135.10 | 21.03 | 0.509 | 2809 (398) | 29.07 | 53.13 | 2.98 |
| *ii)Tailed events* |  |  |  |  |  |  |  |
| July 11 | 144.90 | 18.59 | 0.508 | 3469 (262) | 25.27 | 78.10 | 4.30 |
| **Pollution-related concentration peaks** |  |  |  |  |  |  |  |
| July 5 | 64.57 | 18.76 | 0.178 | 1776 (354) | - | - | - |
| **Non-event days** |  |  |  |  |  |  |  |
| July 8 | 143.70 | 20.02 | 0.608 | 3142 (551) | 28.86 | 56.77 | 2.43 |
| July 10 | 105.05 | 27.45 | 0.314 | 2880 (142) | 25.99 | 71.73 | 6.18 |
| July 16 | 113.49 | 17.12 | 0.354 | 3152 (237) | 24.32 | 73.94 | 6.95 |
|  |  |  |  |  |  |  |  |

Table S2. Calculated average CS and Coagulation sink for the NPF event days, the failed events, pollution-related concentration peaks, and non-event days.

| **NPF event days** | Duration of NPF event [h] | CS _H_2_SO_4_ [s^-1^] | CS _OOM [s^-1^] | CS _BrO  [s^-1^] | CS _CHBr_3_  [s^-1^] | Coagulation sink [s^-1^] |
| --- | --- | --- | --- | --- | --- | --- |
| July 6 | 8:00-12:00 | 6.36·10^-3^ | 2.71·10^-3^ | 6.19·10^-3^ | 3.77·10^-3^ | 3.60·10^-6^ |
| July 9 | 14:00-23:59 | 8.79·10^-3^ | 3.75·10^-3^ | 9.30·10^-3^ | 5.66·10^-3^ | 1.55·10^-6^ |
| July 14 | 11:40-19:00 | 7.33·10^-3^ | 3.13·10^-3^ | 7.50·10^-3^ | 4.57·10^-3^ | 2.47·10^-6^ |
| July 15 | 13:00-19:00 | 5.61·10^-3^ | 2.39·10^-3^ | 6.07·10^-3^ | 3.70·10^-3^ | 1.29·10^-6^ |
| July 17 | 8:00-12:00 | 7.61·10^-3^ | 3.24·10^-3^ | 3.45·10^-3^ | 2.10·10^-3^ | 3.09·10^-6^ |
| **Failed events**  *i)Quasi events* |  |  |  |  |  |  |
| July 7 | - | 8.68·10^-3^ | 3.70·10^-3^ | 1.09·10^-2^ | 6.63·10^-3^ | - |
| July 12 | - | 1.27·10^-2^ | 5.41·10^-3^ | 1.59·10^-2^ | 9.69·10^-3^ | - |
| July 13 | - | 9.74·10^-3^ | 4.15·10^-3^ | 1.22·10^-2^ | 7.44·10^-3^ | - |
| *ii)Tailed events* |  |  |  |  |  |  |
| *July 11* | - | 1.05·10^-2^ | 4.47·10^-3^ | 1.31·10^-2^ | 8.00·10^-3^ | - |
| **Pollution-related concentration peaks** |  |  |  |  |  |  |
| July 5 | - | 4.78·10^-3^ | 2.04·10^-3^ | 5.99·10^-3^ | 3.65·10^-3^ | - |
| **Non-event days** |  |  |  |  |  |  |
| July 8 | - | 9.98·10^-3^ | 4.26·10^-3^ | 1.25·10^-2^ | 7.62·10^-3^ | - |
| July 10 | - | 8.16·10^-3^ | 3.48·10^-3^ | 1.02·10^-2^ | 6.23·10^-3^ | - |
| July 16 | - | 8.75·10^-3^ | 3.73·10^-3^ | 1.10·10^-2^ | 6.68·10^-3^ | - |

Table S3. The calculated average concentration of different vapors necessary to obtain the measured growth rates.

| **NPF event days** | H_2_SO_4_ [cm^-3^] | OOM [cm^-3^] | BrO [cm^-3^] | CHBr_3_ [cm^-3^] |
| --- | --- | --- | --- | --- |
| July 6 | 2.18·10^8^ | 1.54·10^8^ | 1.77·10^8^ | 1.10·10^8^ |
| July 9 | 6.23·10^7^ | 4.39·10^7^ | 5.07·10^7^ | 3.16·10^7^ |
| July 14 | 3.15·10^7^ | 2.23·10^7^ | 2.57·10^7^ | 1.60·10^7^ |
| July 15 | 1.46·10^7^ | 1.03·10^7^ | 1.19·10^7^ | 7.40·10^6^ |
| July 17 | 7.67·10^7^ | 5.41·10^7^ | 6.25·10^7^ | 3.89·10^7^ |

Table S4. Elemental analysis of ultrafine mode particles from Rogoznica during NPF event and non-event days (average concentration).

|  | Na  [µg m^-3^] | Mg  [µg m^-3^] | K  [µg m^-3^] | Ga  [µg m^-3^] | Br  [µg m^-3^] |
| --- | --- | --- | --- | --- | --- |
| NPF event day | 4.80 | 4.10 | 0.051 | 0.0079 | 0.132 |
| Non-event day | 1.168 | 0.13 | 0.25 | 0.025 | 0.037 |

Elevated concentrations of elements of natural origin were detected during the event days (Na, Mg, and Br), whereas, during the non-event days, higher concentrations of anthropogenic elements were observed (Ga and K).

Table S5. The organic carbon analysis of the ultrafine mode particles (30-100nm) collected in Rogoznica during the NPF event days, failed events, and non-event days.

| **NPF event day** | OC [%] |
| --- | --- |
| July 6, 2021 | 76.09 |
| July 9, 2021 | 77.23 |
| July 14, 2021 | 79.23 |
| July 15, 2021 | 64.66 |
| **Non-event days** |  |
| July 8, 2021 | 45.96 |
| **Tailed event** |  |
| July 11, 2021 | 28.97 |

**Correlation Analysis**

Supporting Information Figure 23 depicts the relationship between the apparent formation rate (*J*_15_) and the CS for various candidate vapors. It is observed that the correlation between *J*_15_ and OOM (*r_Spearman_* = -0.6, *p =0.35*) is negative, indicating an inverse relationship between CS and *J*_15_; as CS increases, *J*_15_ will decrease and vice versa. It should be noted that the strength of the correlation is not significant. The lack of significance in the correlation might be due to the restricted number of observed days. However, it is imperative to highlight the negative correlation trend, indicating the potential involvement of condensable OOMs in forming and growing particles. The lower CS for OOMs implies a slower consumption in the condensation process, theoretically enabling greater utilization of OOMs in particle formation and increasing the apparent formation rate.

**
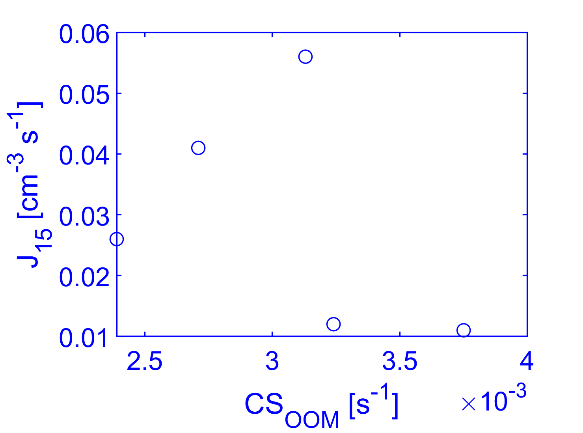

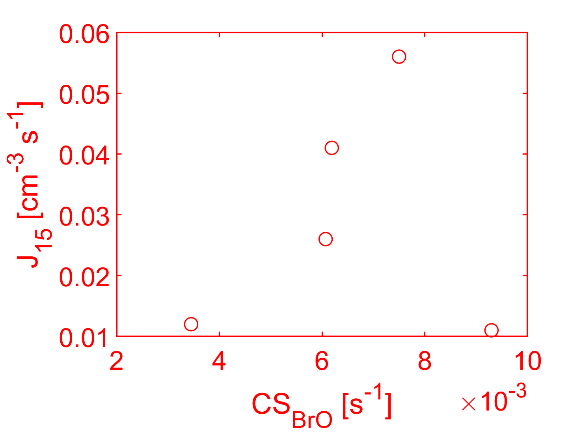
**

A

B

**
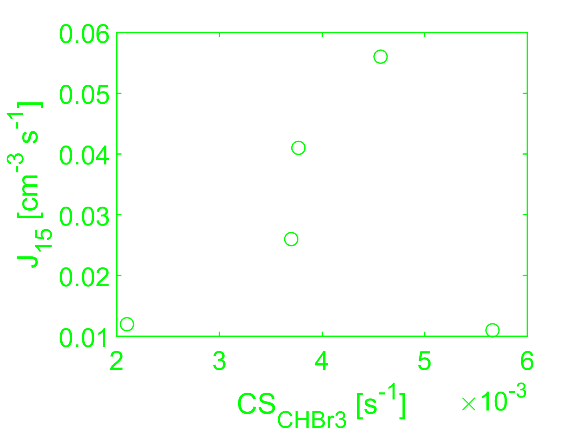
**

C

Supporting Information Figure 23. Scatter plot of *J*_15_ vs CS for different vapor candidates. A) *J*_15_ vs. CS for OOM, B) *J*_15_ vs. CS for BrO^-^ and C) *J*_15_ vs. CHBr_3_

Contrary to this, the correlations between CS for BrO^-^ and CHBr_3_ with *J*_15_ (*r_Spearman_* = 0.156, *p = 0.8* and *r_Spearman_* = 0.158, *p = 0.7* respectively) are positive. However, these correlations are not statistically significant, likely due to the limited number of observed days. Nonetheless, the positive correlation trend suggests higher CS values correspond to higher *J*_15_ values. A negative trend would have been expected if BrO^-^ or CHBr_3_ were involved in particle formation. Therefore, based on the CS and *J*_15_ calculation and the chemical analysis of ultrafine mode particles, we can infer that OOMs would have a more significant role as nucleation agents in particle formation than the Br species in Rogoznica area.

As recent literature [[21](#_ENREF_23), [25](#_ENREF_28)] has highlighted, reactive Br species can participate in the oxidation of VOCs, either as direct oxidizing agents or by enhancing the concentrations of conventional atmospheric oxidants (OH, NO_3_, and O_3_). Based on this, we hypothesize that CHBr_3_ and BrO^-^ could serve as precursors for reactive Br species, potentially contributing to the formation of OOMs. Furthermore, from the results, it can be seen that for higher Br^-^ concertation in the ultrafine mode particles, a lower CS for OOMs is observed (*r* = -0.036), and higher *J*_15_ is also observed for the higher Br^-^ concentration (*r* = 0.04). Suppose we assume that Br^-^ is formed by reducing reactive Br species during the oxidation reaction with VOCs. In that case, it suggests that these reactive Br species significantly influence particle formation and growth by potentially enhancing the availability of OOMs to participate in the particle formation and growth process.

Observations, alongside the calculated CS and *J*_15_ values, indicate that CHBr_3_ and BrO^-^ potentially contribute to particle formation by generating reactive Br species. This process enhances the formation of OOMs, which, in turn, could play a substantial role in particle formation and growth (refer to Table S5). However, CHBr_3_ is water-soluble, and BrO is condensable. These properties suggest their potential contribution to the growth of formed particles. We utilized the semi-empirical first derivative method to calculate the instantaneous growth rate, which, alongside the CS values, offers a comprehensive understanding of CHBr_3_ and BrO^-^'s role in the growth process. If CHBr_3_ and BrO are associated with particle growth, we would expect a positive correlation between the particles' growth rate and the CS values of CHBr_3_ and BrO^-^. The Supporting Information Figure 24 demonstrates a positive correlation between the CS of the Br species and the observed growth rate (*r*BrO = 0.06, *p* = 0.54, *r*ChBr3 = 0.06, *p* = 0.54). However, despite indicating a positive correlation by Spearman's correlation coefficient, the findings suggest that this correlation is not statistically significant. The reason for this is probably a small amount of observation. Based on these observations and the identified positive correlation, it is suggested that CHBr_3_ and BrO^-^ may influence the growth rate. Nevertheless, such a conclusion warrants further investigation in future research endeavors. Moreover, a positive correlation was observed between the growth rate and the CS for OOMs (r = 0.03, p = 0.74). This positive correlation suggests that condensable OOMs may also contribute to particle growth.


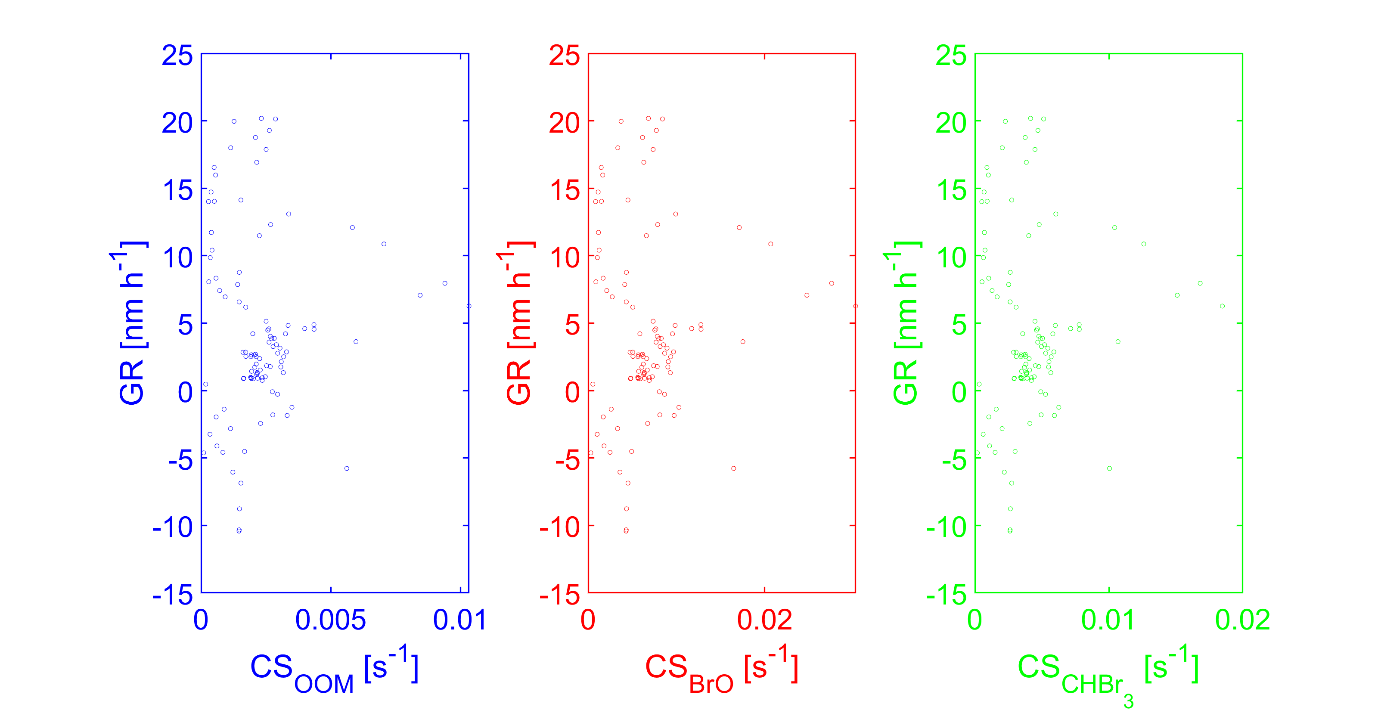


C

B

A

Supporting Information Figure 24. Scatter plot the instantaneous GR for all NPF days vs. the CS for different vapor candidates. A) GR vs. CS_OOM_, B) GR vs. CS_BrO_ and C) GR vs. CS_CHBr3_.

**Modeled backward trajectories and wind rose diagrams for the observed days in Rogoznica**

B


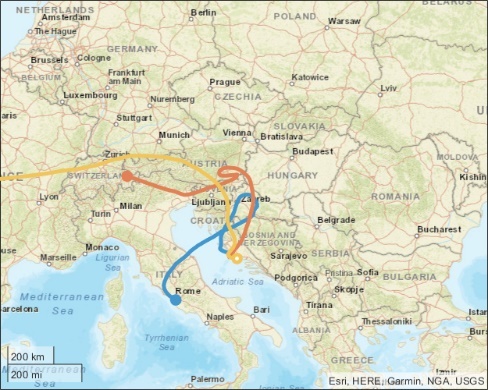

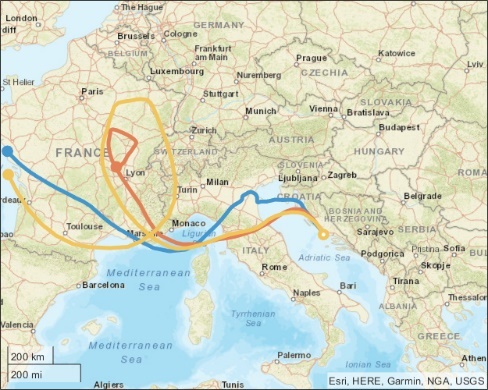


C

A


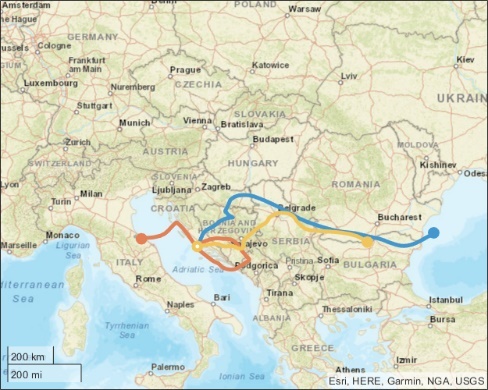


Supporting Information Figure 25. Air-mass backward trajectories during the NPF event days in Rogoznica, as modeled by NOAA HYSPLIT for three different altitudes: blue-100, orange-500, yellow-1000 m, and 72 h duration, modeled backward trajectories for A) July 6, 2021; B) July 15, 2021; and C) July 17, 2021.


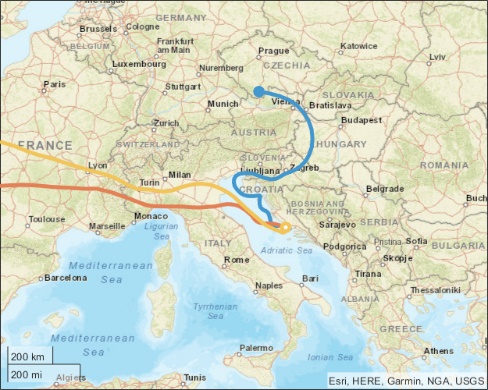

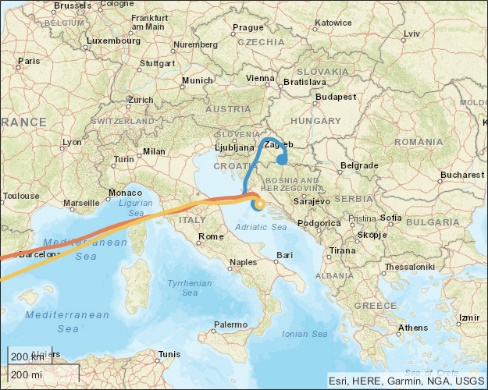


B

A


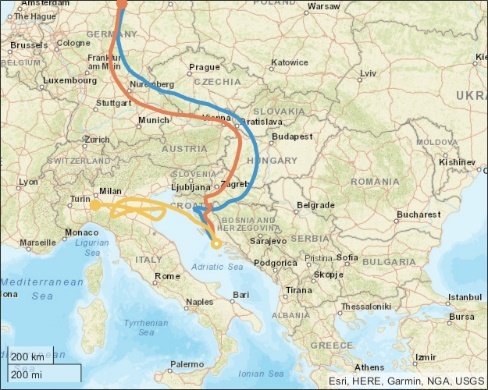

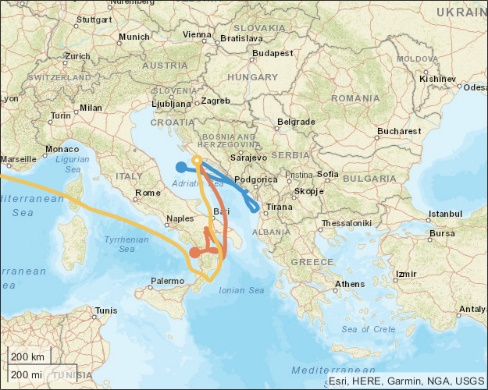


D

D

C


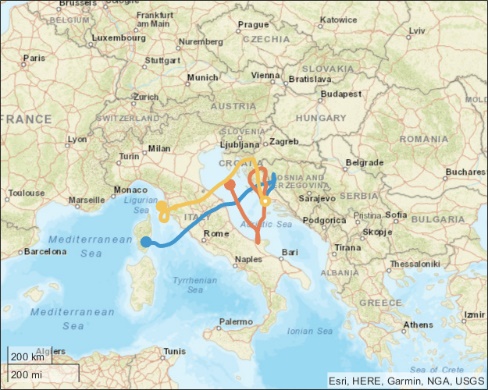


Supporting Information Figure 26. Air-mass backward trajectories during events for which the formation and growth rate were impossible to calculate (failed events and the pollution-related concentration peaks), as modeled by NOAA HYSPLIT for three different altitudes: blue-100, orange-500, yellow-1000 m, and 72 h duration. Quasi events; modeled backward trajectories for A) July 7, 2021, B) July 12, 2021 and C) July 13, 2021. The tailed event; D) modeled backward trajectories for July 11, 2021 and the pollution-related concentration event; E) modeled backward trajectories for July 5, 2021.


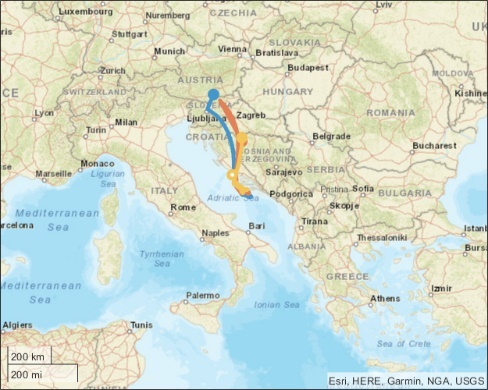

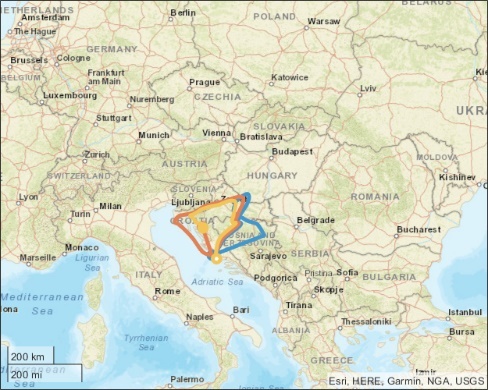


B

A


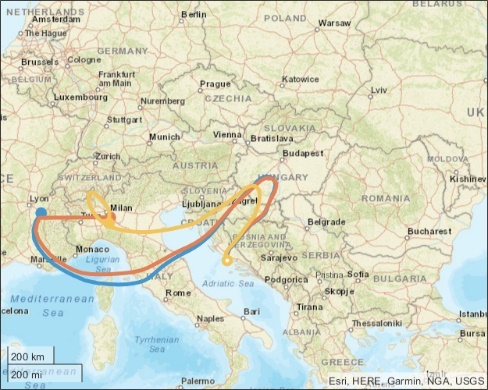


C

Supporting Information Figure 27. Air-mass backward trajectories during non-events for, as modeled by NOAA HYSPLIT for three different altitudes: blue-100, orange-500, yellow-1000 m, and 72 h duration. Non-events; modeled backward trajectories for A) July 8, 2021, B) July 10, 2021 and C) July 16, 2021.


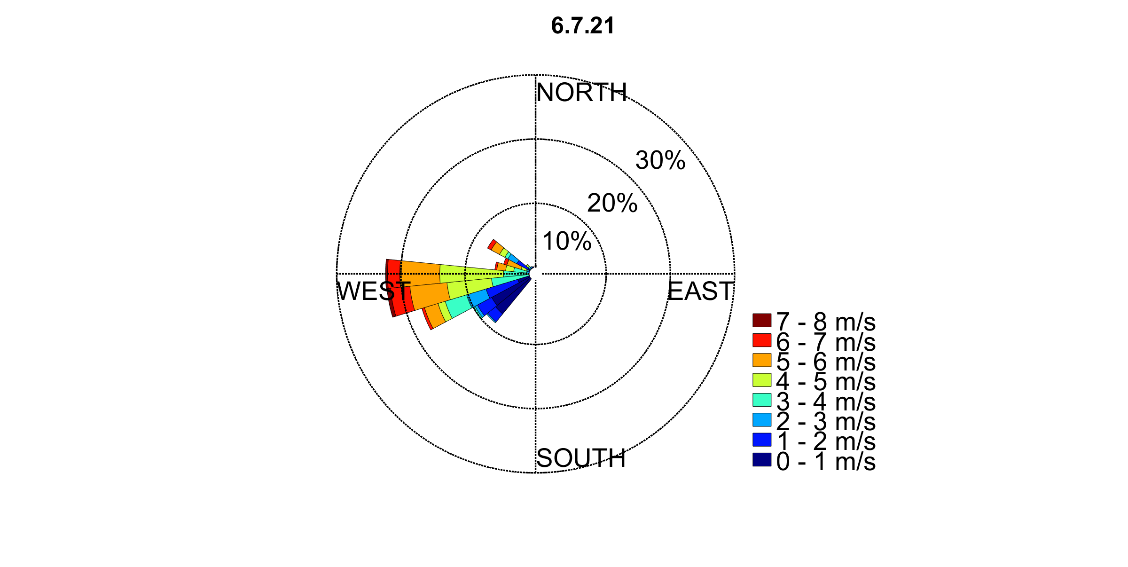

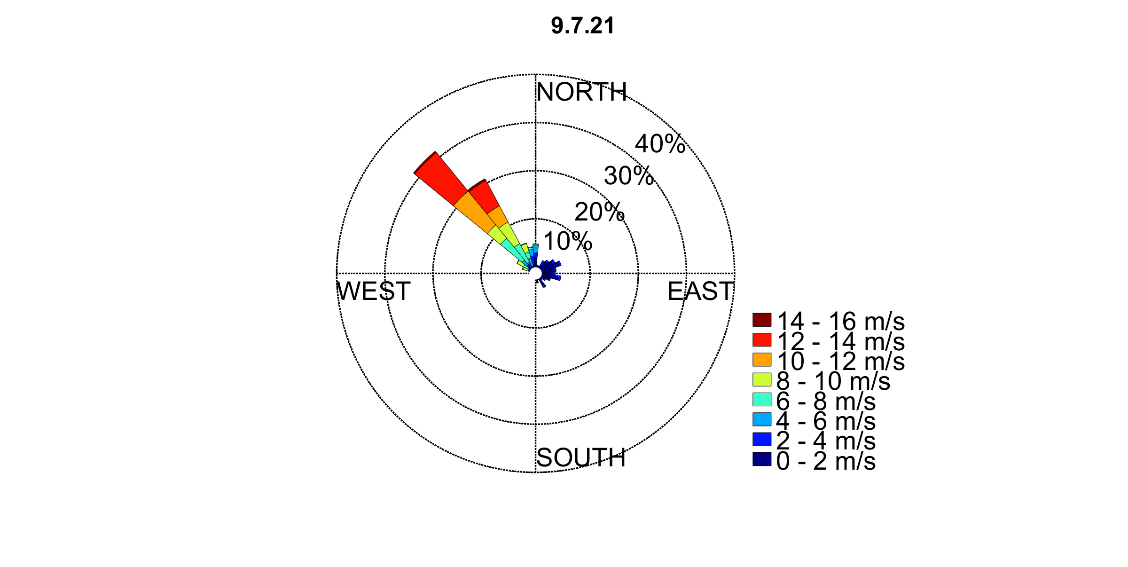


B

A


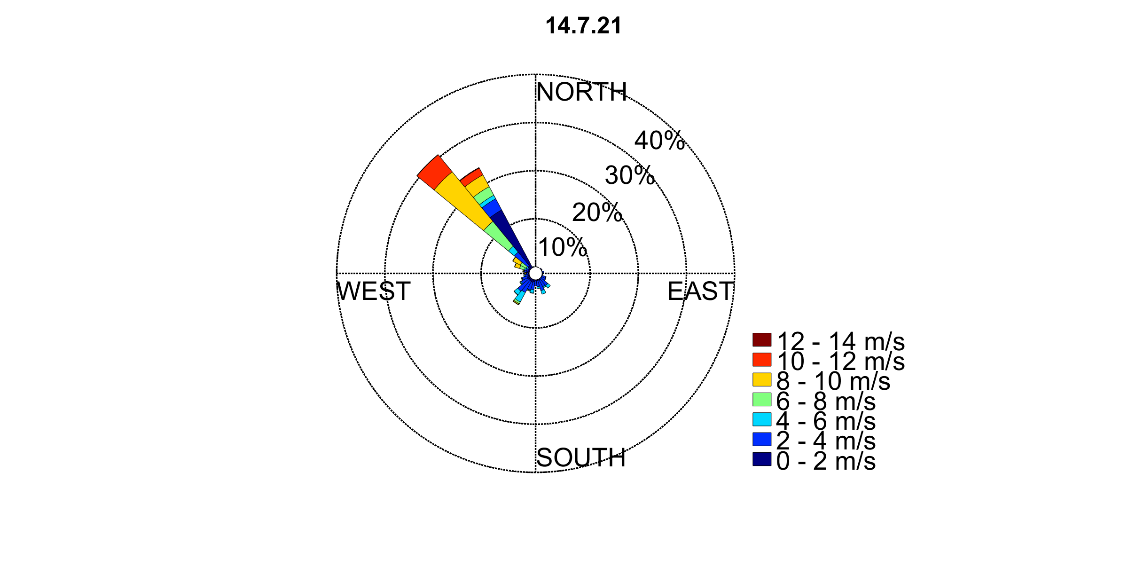

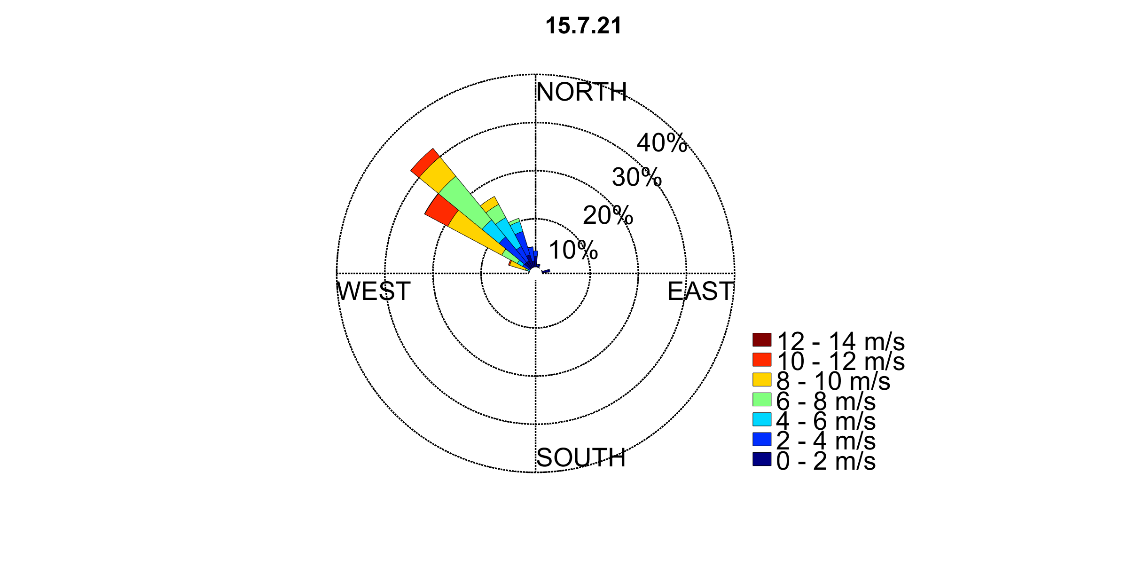


D

C


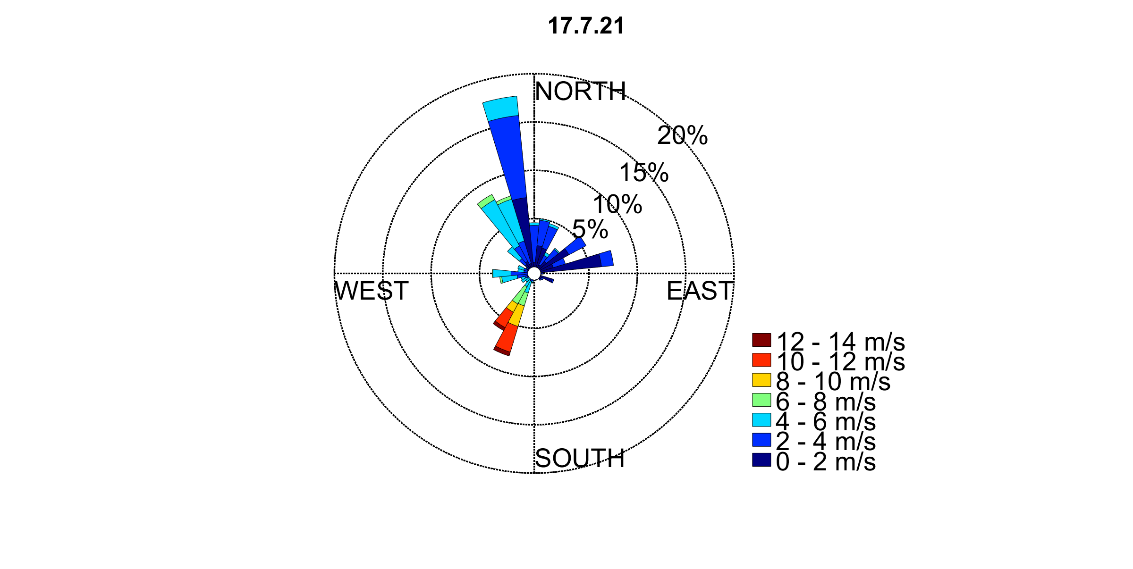


E

Supporting Information Figure 28. Wind Rose Diagram for the NPF event days observed in Rogoznica showing directions of the wind origin at the measuring station. The wind rose diagram observed on A) July 6, 2021, B) July 9, 2021, C) July 14, 2021, D) July 15, 2021, and E) July 17, 2021.

**The Condensation growth rate property**


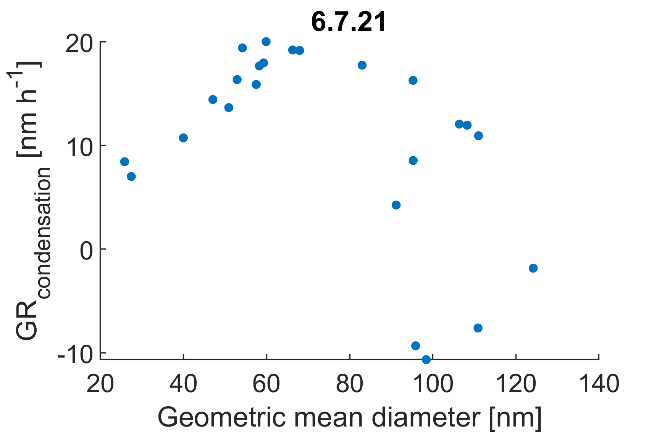

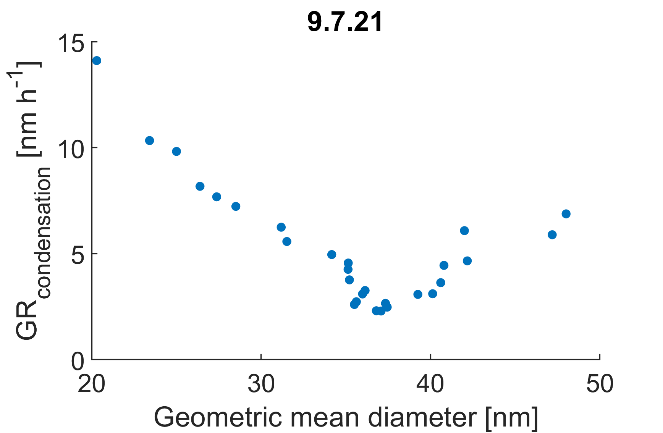


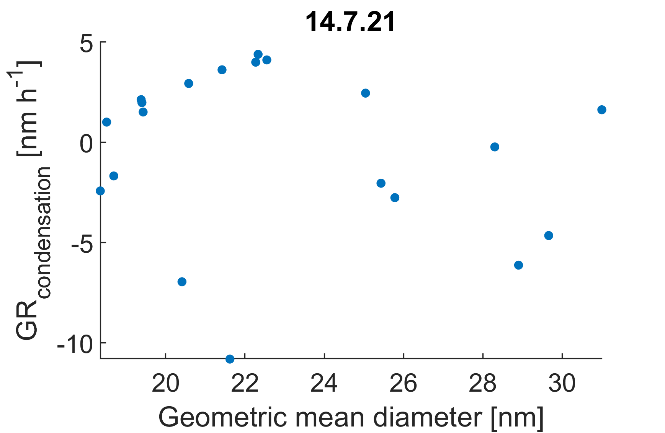

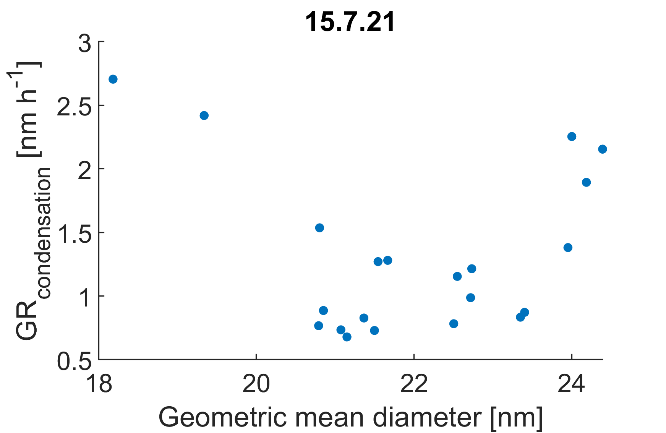


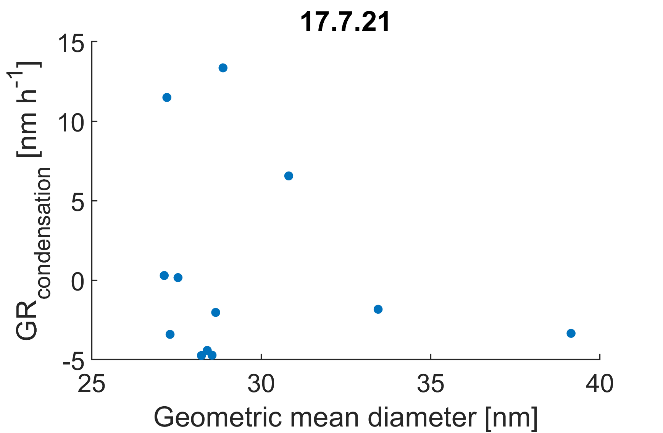


Supporting Information Figure 29. Condensation growth rate (GR condensation) versus geometrical mean diameter (GMD) of the particle population for NPF event days observed in Rogoznica. A) GR condensation versus GMD calculated for the observed particle size distribution on July 6, 2021, B) July 9, 2021, C) July 14, 2021, D) July 15, 2021, and E) July 17, 2021.

**Temperature influence on the condensation growth rate**


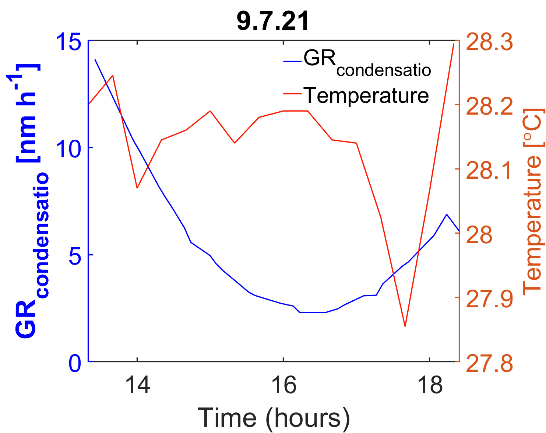

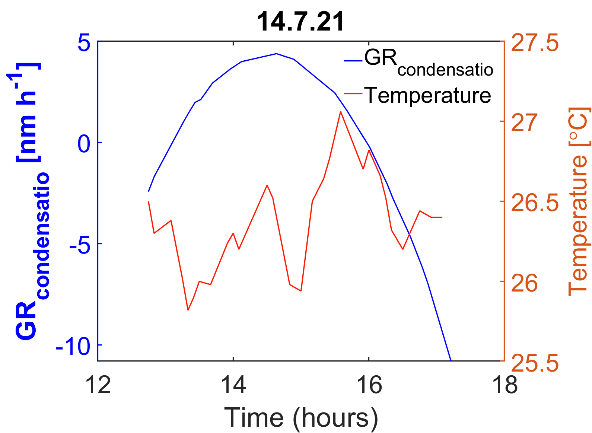


Supporting Information Figure 30. Ambient temperature influence on the condensation growth rate (GR condensation) observed for NPF event days in Rogoznica. A) Temperature influence on the GR condensation for Class I event where the particle grew up to 100 nm on July 9, 2021, and B) Temperature influence on the GR condensation for the Class I event where the particle grew up to 30 nm on July 14, 2021.

**References**

[1] Kačan, B. *POKREĆE SE POSTUPAK ZAŠTITE Rogozničko jezero bit će proglašeno spomenikom prirode*. 2023 [cited 2023 6.7.2023]; Available from: <https://www.morski.hr/pokrece-se-postupak-zastite-rogoznicko-jezero-bit-ce-proglaseno-spomenikom-prirode/>.

[2] O'Dowd, C.D., et al., *A dedicated study of New Particle Formation and Fate in the Coastal Environment (PARFORCE): Overview of objectives and achievements.* Journal of Geophysical Research: Atmospheres, 2002. **107**(D19): p. PAR 1-1-PAR 1-16.

[3] Kerminen, V.-M., et al., *Atmospheric new particle formation and growth: review of field observations.* Environmental Research Letters, 2018. **13**(10): p. 103003.

[4] Maso, M.D., et al., *Formation and growth of fresh atmospheric aerosols: eight years of aerosol size distribution data from SMEAR II, Hyytiälä, Finland.* Boreal Environment Research, 2005. **10**: p. 323-336.

[5] Buenrostro Mazon, S., et al., *Classifying previously undefined days from eleven years of aerosol-particle-size distribution data from the SMEAR II station, Hyytiälä, Finland.* Atmos. Chem. Phys., 2009. **9**(2): p. 667-676.

[6] Sarangi, B., S.G. Aggarwal, and P.K. Gupta, *A Simplified Approach to Calculate Particle Growth Rate Due to Self-Coagulation, Scavenging and Condensation Using SMPS Measurements during a Particle Growth Event in New Delhi.* Aerosol and Air Quality Research, 2015. **15**(1): p. 166-179; Nieminen, T., et al., *Global analysis of continental boundary layer new particle formation based on long-term measurements.* Atmos. Chem. Phys., 2018. **18**(19): p. 14737-14756.

[7] Mäkelä, J.M., et al., *Characteristics of the atmospheric particle formation events observed at a borel forest site in southern Finland.* Boreal Environment Research, 2000. **5**(4): p. 299-313.

[8] Kulmala, M., et al., *Formation and growth rates of ultrafne atmospheric particles: a review of observations. Aerosol Science 35, 143–176*. 2004.

[9] Jeong, C.H., et al., *Particle formation and growth at five rural and urban sites.* Atmos. Chem. Phys., 2010. **10**(16): p. 7979-7995.

[10] Kulmala, M., et al., *Measurement of the nucleation of atmospheric aerosol particles.* Nature Protocols, 2012. **7**(9): p. 1651-1667.

[11] Kerminen, V.M., L. Pirjola, and M. Kulmala, *How significantly does coagulational scavenging limit atmospheric particle production?* Journal of Geophysical Research: Atmospheres, 2001. **106**(D20): p. 24119-24125.

[12] Brean, J., et al., *Open ocean and coastal new particle formation from sulfuric acid and amines around the Antarctic Peninsula.* Nature Geoscience, 2021. **14**(6): p. 383-388.

[13] Wang, Z., et al., *Modeling study of regional severe hazes over mid-eastern China in January 2013 and its implications on pollution prevention and control.* Science China Earth Sciences, 2014. **57**(1): p. 3-13.

[14] Seinfeld, J.H. and S.N. Pandis, *Atmospheric chemistry and physics: from air pollution to climate change*. 2016: John Wiley & Sons.

[15] Kulmala, M., et al., *On the formation, growth and composition of nucleation mode particles.* Tellus B: Chemical and Physical Meteorology, 2001. **53**(4): p. 479-490.

[16] Smith, J.N., et al., *Chemical composition of atmospheric nanoparticles formed from nucleation in Tecamac, Mexico: Evidence for an important role for organic species in nanoparticle growth.* Geophysical Research Letters, 2008. **35**(4); Bzdek, B.R., et al., *Quantitative Assessment of the Sulfuric Acid Contribution to New Particle Growth.* Environmental Science & Technology, 2012. **46**(8): p. 4365-4373.

[17] Kivekäs, N., et al., *Coupling an aerosol box model with one-dimensional flow: a tool for understanding observations of new particle formation events.* Tellus B: Chemical and Physical Meteorology, 2016. **68**(1): p. 29706.

[18] Ehn, M., et al., *A large source of low-volatility secondary organic aerosol.* Nature, 2014. **506**(7489): p. 476-479.

[19] Kulmala, M., *Nucleation as an aerosol physical problem. University of Helsinki, Department of Physics*. 1988, Ph. D. thesis.

[20] Du, W., et al., *Influence of Aerosol Chemical Composition on Condensation Sink Efficiency and New Particle Formation in Beijing.* Environmental Science & Technology Letters, 2022. **9**(5): p. 375-382.

[21] Xia, M., et al., *Pollution-Derived Br2 Boosts Oxidation Power of the Coastal Atmosphere.* Environmental Science & Technology, 2022. **56**(17): p. 12055-12065.

[22] Ciglenečki, I., et al., *The possibilities of voltammetry in the study reactivity of dissolved organic carbon (DOC) in natural waters.* Journal of Solid State Electrochemistry, 2023.

[23] Rice, O.K., *The Surface Tension and the Structure of the Surface of Aqueous Ammonia Solutions.* The Journal of Physical Chemistry, 1928. **32**(4): p. 583-592; Donaldson, D.J. and K.T. Valsaraj, *Adsorption and Reaction of Trace Gas-Phase Organic Compounds on Atmospheric Water Film Surfaces: A Critical Review.* Environmental Science & Technology, 2010. **44**(3): p. 865-873.

[24] Schofield, R.K., E.K. Rideal, and W.B. Hardy, *The kinetic theory of surface films.—Part II. Gaseous, expanded and condensed films.* Proceedings of the Royal Society of London. Series A, Containing Papers of a Mathematical and Physical Character, 1926. **110**(753): p. 167-177.

[25] Li, Q., et al., *Halogens Enhance Haze Pollution in China.* Environmental Science & Technology, 2021. **55**(20): p. 13625-13637.
